# Functional analysis of long intergenic non-coding RNAs in phosphate-starved rice using competing endogenous RNA network

**DOI:** 10.1038/srep20715

**Published:** 2016-02-10

**Authors:** Xi-Wen Xu, Xiong-Hui Zhou, Rui-Ru Wang, Wen-Lei Peng, Yue An, Ling-Ling Chen

**Affiliations:** 1 National Key Laboratory of Crop Genetic Improvement, Huazhong Agricultural University, Wuhan 430070, P.R. China; 2 College of Informatics, Agricultural Bioinformatics Key Laboratory of Hubei Province, Huazhong Agricultural University, Wuhan 430070, P.R. China

## Abstract

Long intergenic non-coding RNAs (lincRNAs) may play widespread roles in gene regulation and other biological processes, however, a systematic examination of the functions of lincRNAs in the biological responses of rice to phosphate (Pi) starvation has not been performed. Here, we used a computational method to predict the functions of lincRNAs in Pi-starved rice. Overall, 3,170 lincRNA loci were identified using RNA sequencing data from the roots and shoots of control and Pi-starved rice. A competing endogenous RNA (ceRNA) network was constructed for each tissue by considering the competing relationships between lincRNAs and genes, and the correlations between the expression levels of RNAs in ceRNA pairs. Enrichment analyses showed that most of the communities in the networks were related to the biological processes of Pi starvation. The lincRNAs in the two tissues were individually functionally annotated based on the ceRNA networks, and the differentially expressed lincRNAs were biologically meaningful. For example, XLOC_026030 was upregulated from 3 days after Pi starvation, and its functional annotation was ‘cellular response to Pi starvation’. In conclusion, we systematically annotated lincRNAs in rice and identified those involved in the biological response to Pi starvation.

Inorganic phosphate (Pi) is essential for the growth and productivity of plants; however, those in agricultural environments can be exposed to Pi starvation^1^. Understanding the biological responses of plants to Pi starvation is vital for improving the efficiency of Pi use and maintaining an acceptable yield^2^. A number of studies have attempted to investigate the complex mechanisms regulating Pi homeostasis in rice, and have reported regulation at the transcript level^3^,^4^,^5^,^6^. Long integrate non-coding RNAs (lincRNAs) exist in both mammalian and plants and may play widespread roles in gene regulation and other biological processes^7^,^8^,^9^, however, the function of lincRNAs that response to Pi starvation are poorly understood.

The competing endogenous RNA (ceRNA) theory has been proved and is now acknowledged widely^10^,^11^. This theory states that ceRNAs, including mRNA, lincRNAs, pseudogenes, and other microRNAs (miRNA) sponges, share common miRNA binding sites and can act as molecular sponges because the amount of a given miRNAs is limited^11^. LincRNAs compete with other miRNA sponges to play important roles in both plants and animals^9^,^12^,^13^,^14^,^15^. In addition, ceRNA networks are useful for studying cancer biology and other biological problems^16^,^17^,^18^,^19^. However, to our knowledge, ceRNA networks have not yet been used to study the functions of lincRNAs in plants such as *Arabidopsis* and rice.

Based on the hypothesis that lincRNAs compete with genes to play important roles in rice undergoing Pi starvation, we used ceRNA networks to study the functions of these lincRNAs. First, we identified lincRNAs in rice by using RNA sequencing (RNA-seq) data from a previous time-series experiment in which plants were exposed to Pi-starved or Pi-sufficient conditions^6^. Second, based on predictions of miRNA-gene and miRNA-lincRNA target pairs, we used a hypergeometric cumulative distribution function test to select ceRNA pairs with common miRNA regulators and to identify those that constitute a ceRNA network. Third, based on the hypothesis that the function of a given lincRNA may be the same as those of genes in the same community or those of genes it directly connected to, we predicted the functions of the lincRNAs in the ceRNA networks. Finally, to determine whether they play important roles in the adaption of rice to Pi starvation, we examined the differentially expressed lincRNAs that had the highest numbers of neighbors in the network.

## Results

### Genome-wide identification of lincRNAs in rice

**Figure 1:**
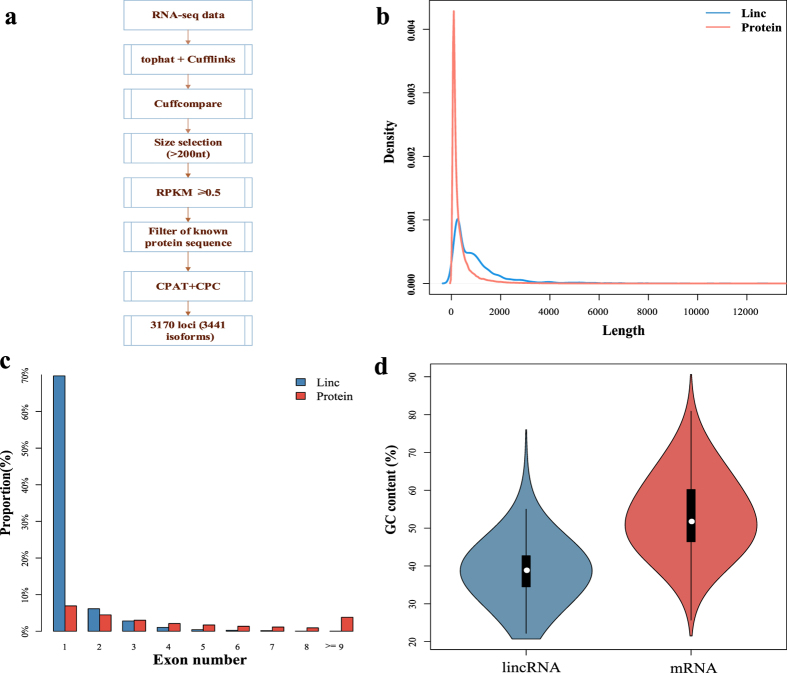
The basic characteristics of lincRNAs in rice. **(a)** A flow chart of the method used to identify the lincRNAs. **(b)** The distributions of exons’ lengths in lincRNAs and protein-coding transcripts. **(c)** The proportions of exons’ number per transcript for lincRNAs and protein-coding transcripts. **(d)** The GC content of the lincRNAs and protein-coding transcripts.

The pipeline shown in Fig. 1a was used to identify lincRNAs from the RNA-seq data of rice undergoing Pi starvation^6^. In brief, if a longer-than-200 nt transcript with no coding capability is located in the intergenic regions and is not similar to known protein-coding genes, it is identified as a candidate lincRNA. The details of the pipeline are shown as follow.

First the next generation sequencing (NGS) quality control (QC) toolkit^20^ was used to filter out low quality reads. Subsequently, the tophat tool^21^ was used to map the filtered reads to the rice reference genome (Oryza_sativa.IRGSP-1.0.21; Ensembl Plants). Samtools^22^ was used to merge three biological replicates. We used gtf file to guide RABT assembly with cufflinks, and merged all assemblies into a final transcript using cuffmerge^23^. Finally, cuffcompare was used to select transcripts in the intergenic region^23^. In addition, small transcripts (shorter than 200 nucleotides) and infrequently expressed transcripts with RPKM <0.5 in all samples were filtered out. Among the retained transcripts, those similar to known protein-coding genes (coverage >50% and e-value <10^−5^) in the UniProt TrEMBL database^24^ were removed. Furthermore, the transcripts with potential coding capabilities, which were identified using the Coding Potential Assessment Tool (CPAT)^25^ and the Coding Potential Calculator^26^, were removed from the retained transcripts. Subsequently, the remaining large transcripts that were expressed frequently and did not overlap with known genes were identified as lincRNAs in rice. A total of 3,170 loci (3,441 isoforms) were obtained from the RNA-seq data.

Next, we compared the genomic features of the identified lincRNAs with those of protein-coding genes in rice. The mean exon length of the lincRNA was larger than that of the mRNA (Fig. 1b), while more than 70% of the lincRNAs, but less than 10% of the mRNAs, contained only one exon (Fig. 1c). In the meanwhile, lincRNAs in rice have fewer, but longer, exons than mRNAs^9^. The GC content of the lincRNAs was also lower than that of the mRNAs (Fig. 1d).

### Topological analysis of the ceRNA networks

**Figure 2:**
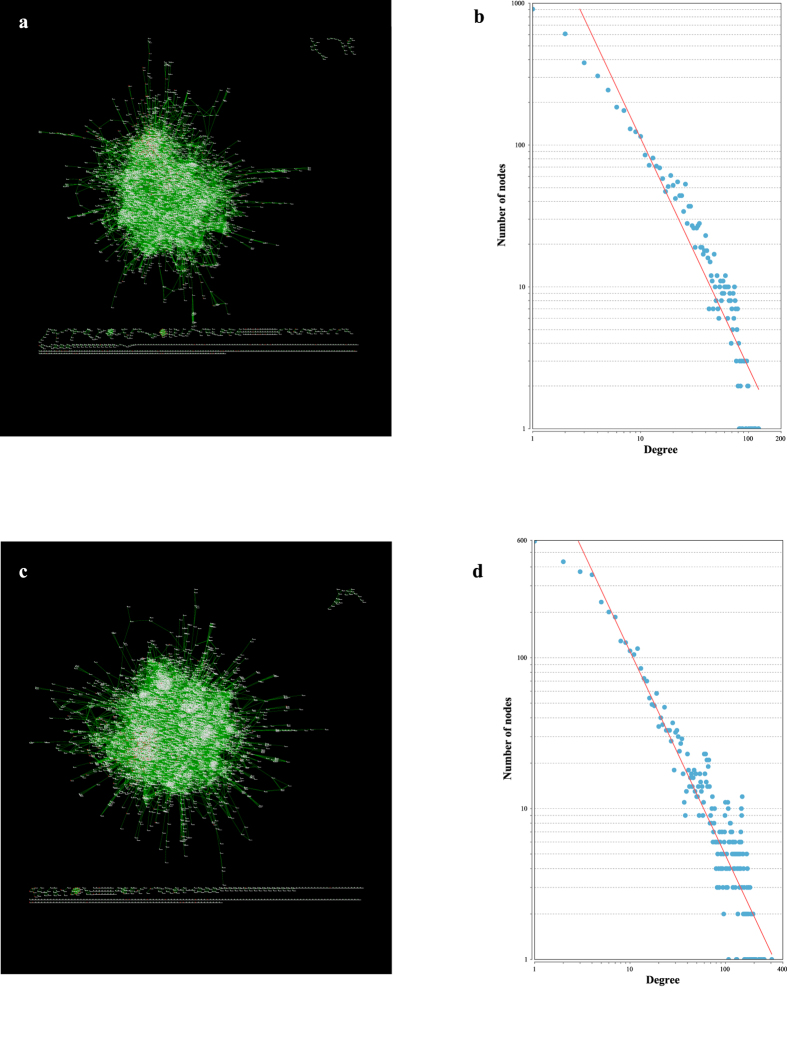
The ceRNA network of the rice root and shoot. **(a)** An overview of the ceRNA network for the rice root. **(b)** The power law fit of the nodes’ degrees for the rice root. **(c)** An overview of the ceRNA network for the rice shoot. **(d)** The power law fit of the nodes’ degrees for the rice shoot.

Based on the miRNA-gene and miRNA-lincRNA relationships, a hypergeometric cumulative distribution function test was used to identify RNA pairs that may compete with each other for binding to the limited number of miRNA. For the rice root samples, Spearman correlation analysis was used to select ceRNA pairs in 27 Pi-starved samples; the resulting ceRNA network contained 31,794 ceRNA pairs. The network comprised 4,847 nodes (511 lincRNAs) with an average degree of 13.12, indicating that the network was very dense (Fig. 2a). The denseness of the network indicated the ceRNA phenomenon may be common in rice roots undergoing Pi starvation. In addition, the degrees of the nodes fit the power law distribution well, with a correlation of 0.91 and an R-squared value of 0.88 (Fig. 2b).

A ceRNA network of the shoot was also generated from the RNA-seq data; this network comprised 4,979 nodes (376 lincRNAs) and 63,660 edges (Fig. 2c). The average degree of the nodes was 25.57, indicating that the ceRNA network of the shoot is denser than that of the root. As observed for the root, the degrees of the nodes in the shoot network fit the power law distribution well, with a correlation of 0.87 and an R-squared value of 0.87 (Fig. 2d).

Taken together, these results indicate that the ceRNA networks for the two tissues were scale-free and had similar topologies; therefore, we were able to use the topological components, such as the communities and hubs, to investigate the biological significance of the networks.

### Functional annotation of the dense sub-networks

The elements in dense sub-networks of a ceRNA network may compete with each other to act as a functional unit; hence, we investigated the functions of the sub-networks of the root ceRNA network to determine their relationships to Pi starvation. Enrichment analysis was applied to each community and the most significant GO term was set as the functional annotation of the community.

**Figure 3:**
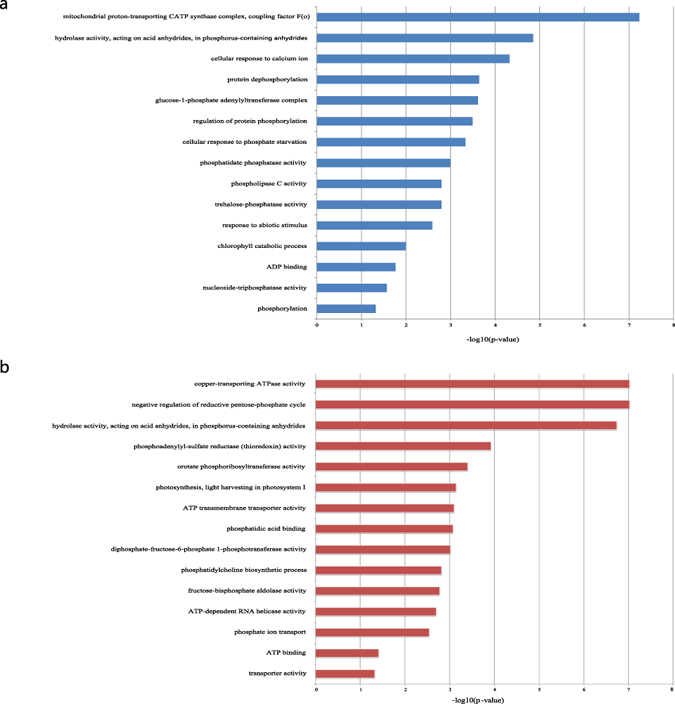
Annotations of the communities in the ceRNA networks of the root (**a**) and shoot (**b**).

A total of 225 dense sub-networks were detected within the ceRNA network of the root. Among them, 200 communities were enriched by GO terms with *P*-values less than 0.05 (Table S1). Some functional annotations of these clusters are shown in Fig. 3a. Most of the annotations appear to be biologically meaningful; for example, two clusters were significantly enriched with ‘nucleoside-triphosphatase activity’ (GO: 0017111) or ‘phospholipase C activity’ terms (GO: 0004629). Pi is a key component of ATP, nucleic acids, and phospholipids^27^. In addition, increased secretion of phosphatase is an adaptive response of plants to Pi starvation^2^,^28^, and ‘phosphatidate phosphatase activity’ (GO: 0008195) was identified as a functional annotation of a cluster in the network. Furthermore, when incorporated into ATP, Pi is the essential substrate of energy metabolism^2^, and ‘mitochondrial proton-transporting ATP synthase complex coupling factor F (o)’ (GO: 0000276), ADP binding (GO: 0043531) were identified as the functional annotation of two clusters. The clusters in the root ceRNA network were also enriched by GO terms related to cellular response to external stimuli, including ‘cellular response to calcium ion’ (GO: 0005509), ‘response to abiotic stimulus’ (GO: 0009628), and ‘cellular response to Pi starvation’ (GO: 0016036), the latter of which validated our analysis method. A potential relationship between calcium accumulation and the adaption of tomato to Pi starvation has been described previously^27^,^28^, which may explain the observed functional annotation of ‘cellular response to calcium ion’. Finally, the clusters in the ceRNA network of the root were also enriched by the GO terms ‘protein dephosphorylation’ (GO: 0006470) and ‘phosphorylation’ (GO: 0016310).

The ceRNA network of the shoot contained 305 dense communities, 273 of which were significantly enriched by GO terms (Table S2). Some of the annotations are shown in Fig. 3b. Similar to the annotations of the clusters in the root ceRNA network, some shoot network clusters were annotated by ATP-related GO terms (‘copper-transporting ATPase activity’, ‘ATP transmembrane transporter activity’, ‘ATP-dependent RNA helicase activity’, and ‘ATP binding’), and some were enriched by phospholipids-related GO terms (‘phosphatidic acid binding’ and ‘phosphatidylcholine biosynthetic process’). Unlike the root sub-networks, the dense sub-networks in the shoot were annotated by photosynthesis (‘photosynthesis, light harvesting in photosystem I’) and transport-related (‘Pi ion transport’, ‘transporter activity’) GO terms. Similarly, Pi starvation-responsive genes in *Arabidopsis* are reported to be involved in photosynthesis and transporter facilitation^27^,^29^.

Based on these findings that the dense sub-networks of the ceRNA networks in the rice root and shoot were annotated by Pi starvation-related GO terms, we concluded that the ceRNA networks identified here can reveal the biological mechanisms operating in rice adapting to Pi starvation.

### Functional annotation of lincRNAs involved in Pi starvation

**Figure 4:**
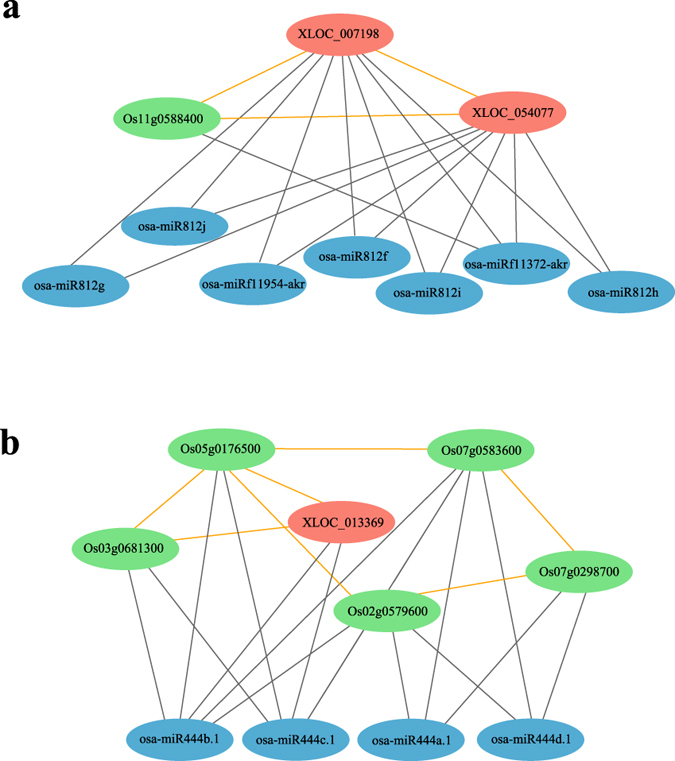
Examples of two communities in the ceRNA networks. The orange-red, green, and blue nodes denote lincRNAs, genes, and miRNAs, respectively. Each sub-network containing nodes with orange edges represents a community in the ceRNA network. Each gray edge denotes a target relationship between a miRNA and a gene or lincRNA, which is hidden in the ceRNA network. **(a)** The ‘ADP binding’ cluster in the ceRNA network of the root. **(b)** The ‘mitochondrial proton-transporting ATP synthase complex assembly’ cluster in the ceRNA network of the shoot.

Investigating the function of the community to which a specific lincRNA belongs, or those of its direct neighbors in a ceRNA network, can be used to predict the function of the lincRNA. Of the 511 lincRNAs in the root network, 121 were successfully annotated with GO terms (Table S3). Among these lincRNAs, some were directly annotated to Pi starvation-related GO terms; for example, seven (XLOC_009323, XLOC_010233, XLOC_026030, XLOC_026206, XLOC_036449, XLOC_051315, XLOC_054628) were annotated as ‘cellular response to phosphate starvation’. In addition, the function of XLOC_066660 was identified as ‘phospholipase C activity’, and the function of two lincRNAs (XLOC_024108 and XLOC_040357) was identified as ‘phosphatidylinositol binding’. Furthermore, two lincRNAs (XLOC_036169 and XLOC_040618) were annotated as ‘phosphorylation’, and two (XLOC_007198, XLOC_054077) were annotated as ‘ADP binding’. The sub-network (cluster 140) to which XLOC_007198 and XLOC_054077 belonged (Fig. 4a; the nodes with orange edges indicate a cluster) was annotated as ‘ADP binding’ and contained the gene ‘Os11g0588400’, suggesting that this gene and these two lincRNAs may compete with each other for binding to their target miRNAs (osa-miRf11372-akr, etc.) during regulation of the ADP binding process.

Among the 376 lincRNAs in the shoot ceRNA network, 164 were annotated with GO terms (Table S4). The functions of XLOC_027908 and XLOC_025912 were predicted as ‘ATP-dependent helicase activity’ (GO: 0008026) and ‘ATP-dependent RNA helicase activity’ (GO: 0004004), respectively. XLOC_037969, XLOC_045026 and XLOC_056566 were annotated as ‘phosphatidylcholine biosynthetic process’ (GO: 0006656), and XLOC_013369 was annotated as ‘mitochondrial proton-transporting ATP synthase complex assembly’ (GO: 0033615). Figure 4b shows the sub-network containing XLOC_013369; in this network, the cluster constructed by the nodes with orange edges was community 188, the function of which was ‘mitochondrial proton-transporting ATP synthase complex assembly’, suggesting that XLOC_013369 may compete with its neighbors during this cellular process.

Similar to the dense sub-networks in the ceRNA network, the functions of a large number of the lincRNAs were related to ATP reactions or compounds. As mentioned earlier, Pi in the form of ATP is an important substrate of energy metabolism^2^ and Pi starvation will influence the ATP content of plants^30^,^31^. In addition, Pi is an important element of phosphatide^27^, and phosphatide or phosphatide metabolism-related GO terms were identified as another common function of the lincRNAs. The most interesting annotation of the lincRNAs was ‘cellular response to Pi starvation’, which may indicate that some of the lincRNAs play a role in the metabolic adaptations of rice undergoing Pi starvation.

### Differentially expressed hubs play essential roles during Pi starvation

**Figure 5:**
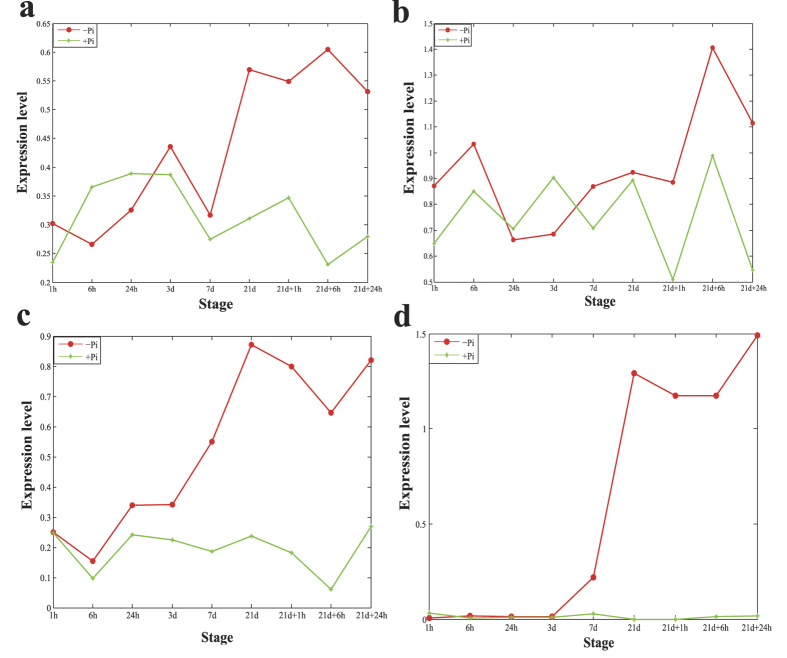
The expression levels of four lincRNAs across all stages of Pi starvation. **(a)** The expression levels of XLOC_026030 in the root across all time points. (**b**) The expression levels of XLOC_054628 in the root across all time points. (**c**) The expression levels of XLOC_037969 in the shoot across all time points. (**d**) The expression levels of XLOC_058915 in the shoot across all time points.

**Figure 6:**
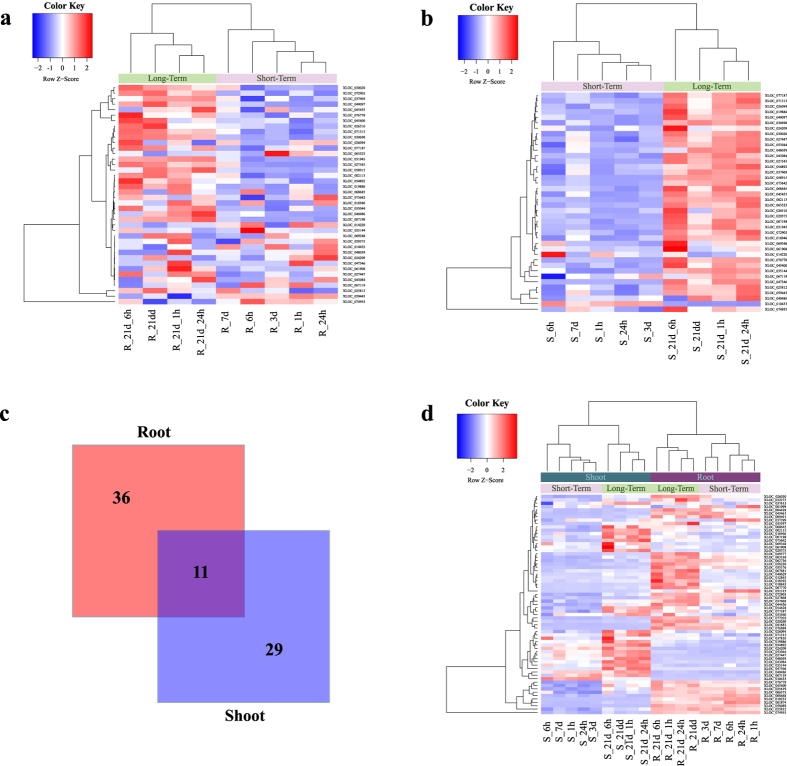
Hierarchical clustering using the key lincRNAs as features. **(a,b)** Clustering of the samples using the key lincRNAs in the root **(a)** or shoot **(b)**. **(c)** Overlapping key lincRNAs in the rice root and shoot. **(d)** Clustering of the samples in both tissues using the non-overlapping lincRNAs.

Based on the hypothesis that competition between lincRNAs and mRNAs may affect the biological responses to Pi starvation, we selected key lincRNAs as those that were hubs in the ceRNA network and were differentially expressed at any stage of Pi starvation. In the root ceRNA network, lincRNAs with degrees higher than 17 were selected as hubs; 47 of these lincRNAs were differentially expressed in the Pi-starved samples compared with the control samples (Table 1), and most of them were annotated. This phenomenon is reasonable because the hubs are closely connected by other mRNAs, which have a higher chance of being annotated. Among the key lincRNAs in the root network, four (XLOC_026030, XLOC_051315, XLOC_010233 and XLOC_054628) were annotated as being involved in the ‘cellular response to Pi starvation’, indicating that they may play important roles in the adaptation of rice to Pi starvation. XLOC_026030 had a degree of 60 and was upregulated in the Pi-starved samples compared to controls till 3 days, especially after 21 days, it is significantly upregulated (Fig. 5a). Similar to XLOC_026030, XLOC_054628 had 28 neighbors in the ceRNA network, which was also significantly upregulated after 21 days (Fig. 5b). The key lincRNAs were then used as features for hierarchical clustering^32^ of the Pi-starved root samples (Fig. 6a). In line with a previous report^6^, the samples were clustered into two groups: those in the early stage (before 7 days), and the late stage (after 7 days) of Pi starvation. In addition, the expression levels of most of the key lincRNAs were higher in the late stage, which may indicate that those lincRNAs are upregulated when the time of Pi starvation is longer than a period to adapt to Pi starvation.

In the shoot ceRNA network, 40 differentially expressed lincRNAs with degrees higher than 18 were identified as key lincRNAs (Table 2). Among them, 37 lincRNAs were successfully annotated. XLOC_037969, which was annotated as ‘phosphatidylcholine biosynthetic process’, was connected with 22 neighbors in the shoot ceRNA network. Analysis of the expression levels of XLOC_037969 across all of the samples and time points revealed that it was upregulated significantly in the Pi-starved samples compared with the control samples until the 7 day time point (Fig. 5c). In addition, with the exception of the 1 h time point, the expression level of this lincRNA was higher in the Pi-starved samples than the control samples at all other time points examined. Pi is an important element of phosphatide^27^. Some plant organs are able to replace phospholipids with non-phosphorous lipids when Pi is scarce^33^,^34^, and some of the key lincRNAs in the rice shoot were related to ‘lipid biosynthetic processes’ (XLOC_010433) or ‘lipid transport’ (XLOC_058915, XLOC_030698, XLOC_024209, XLOC_077187 and XLOC_026516). We also investigated the express level of XLOC_058915, which was a hub in the ceRNA network of shoot. We found that it was upregulated till 7 days, however, it was not expressed during all time points in the control samples (Fig. 5d). Similar to the analysis of root samples, using the key lincRNAs in the shoot to cluster the Pi starvation samples resulted in the formation of two groups (Fig. 6b): the early stages and late stages of Pi starvation.

Next, the differences between the key lincRNAs in the two tissues were investigated. Only 11 common lincRNAs were identified, indicating that most of the key lincRNAs were unique to each tissue (Fig. 6c). This result is reasonable because lincRNAs display tissue-specific expression patterns^35^,^36^. The non-common lincRNAs from the two tissues were then used as features to cluster the Pi starvation samples (Fig. 6d). Using these lincRNAs, the samples were divided according to the tissue type, and the samples within each tissue were clustered into two groups corresponding to those in the early (before 7 days) and late (after 7 days) stages of Pi starvation.

Based on the results described above, we concluded that the key lincRNAs in both tissues were able to divide the samples into the early and late stages of Pi starvation. Analyses of the expression levels of these lincRNAs revealed that most were differentially expressed after exposure of the rice plants to Pi starvation for more than 7 days, which is in line with a previous report^6^. These findings indicate that the differentially expressed hubs (lincRNAs) in the two tissues may play important roles in the biological responses of rice to Pi starvation.

## Discussions

Genome-wide screening and functional analysis of lincRNAs can advance current knowledge of the biological mechanisms involved in the responses of plants to Pi starvation. We hypothesized that lincRNAs compete with other miRNA sponges (including genes) to play important roles in the adaption of rice to Pi starvation. To investigate this hypothesis, we used ceRNA networks to predict the functions of lincRNAs by analyzing those of their neighbors in the network and the communities to which they belong. A total of 3,170 lincRNAs were identified using RNA-seq data from rice undergoing Pi starvation. The ceRNA networks for the two tissues (root and shoot) were then constructed based on miRNA-target data and the expression level of RNAs in the two tissues. Topological analysis of the two ceRNA networks showed that they were typically biological network and the ceRNA phenomenon is common in rice tissues; thus, it was reasonable to perform a functional analysis of the lincRNAs.

Mining the dense clusters in the two networks identified 225 and 305 clusters in the root and shoot, respectively. Most of these clusters were successfully annotated to GO terms using enrichment analysis. Some of the clusters in the root ceRNA networks were annotated to ATP metabolism-related and phosphatide-related GO terms. Notably, the functional annotation of a cluster was ‘cellular response to Pi starvation’. Similar to those in the root network, some of the clusters in the shoot ceRNA network were annotated to ATP metabolism-related and phosphatide-related GO terms; however, unlike those in the root network, there were also some clusters in the shoot network annotated to photosynthesis and transport-related GO terms. Annotation of the clusters showed that the ceRNA networks could be used to understand the biological responses of rice to Pi starvation; therefore, we determined the function of a given lincRNA as that of the cluster to which it belonged. If a lincRNA was not involved in a cluster, its function was annotated by enrichment analysis of its direct neighbors. As a result, 121 lincRNAs in the root and 164 lincRNAs in the shoot were successfully annotated, and some of these lincRNAs were also related to Pi starvation GO terms.

The differentially expressed lincRNAs that were hubs in the ceRNA networks were identified as key lincRNAs. Investigation of these key lincRNAs showed that they may play important roles in the biological response to Pi starvation. For example, the key lincRNAs were able to distinguish between samples at different stages of Pi starvation. In addition, analyses of the key lincRNAs revealed that some may be involved in the biological processes that allow adaptation to Pi starvation. For example, XLOC_026030, a hub in the root ceRNA network that was annotated as ‘cellular response to Pi starvation’, was upregulated after 3 days exposure to Pi starvation, and was up-regulated further after 7 days exposure to this condition, indicating that this lincRNA may play an adaptive role in rice undergoing Pi starvation.

Overall, this study describes a computational framework to analyze the functions of lincRNAs based on RNA-seq data. The method described here could be used to examine numerous biological processes in plants and animals.

## Methods

### Data sets

To understand the function of lincRNAs in Pi homeostasis in rice, we obtained root and shoot RNA-seq data from a previous study in which rice were exposed to Pi-sufficient (0.32 mM Pi) or Pi-starved (0 mM Pi) conditions for nine different times: 1 h, 6 h, 24 h, 3 days, 7 days, 21 days, 21 days + 1 h, 21 days + 6 h and 21 day + 24 h. Each time point included three Pi-sufficient and three Pi-starved samples^6^. All the RNA-seq data used in this study was downloaded from NCBI SRA (SRA097415). The GO term data for rice, rice genome and annotation file (Oryza_sativa.IRGSP-1.0.21) were downloaded from Ensembl Plants. The miRNA-gene and miRNA-lincRNA relationships were obtained from psRNATarget^37^ with default parameters.

### Data processing

TopHat v2.0.14^21^ with default parameters was used to align each RNA-seq data to rice genome and samtools^22^ was used to merge three biological replicates. We used gtf file to guide RABT assembly with Cufflinks and merged all assemblies into final transcripts using Cuffmerge^23^. Cuffcompare^23^ was used to select transcripts in the intergenic region according to the rice annotation file. Transcripts smaller than 200 nt were removed. Putative lincRNAs are non-coding transcripts longer than 200 nt and transcripts from intergenic region. And then we excluded transcripts similar to known protein-coding genes (coverage >50% and e-value <10 − 5) in the UniProt TrEMBL database^24^. We used CPAT^25^ and CPC^26^ to remove transcripts which have protein-coding potential. Htseq-count was used to count reads. EdgeR^48^ was applied to obtain the normalization of gene expression (RPKM) and differentially expressed genes (FDR <=0.05) between Pi-starved samples and controls. The threshold of RPKM was set as 0.5. For each tissue, the genes and lincRNAs with RPKM values less than the threshold in all of the samples were filtered out.

### Pipeline to construct the ceRNA networks

The ceRNA networks were constructed using a similar strategy to that used in our previous study^19^. First, all of the miRNA regulators of a given RNA (mRNA or lincRNA) were selected using psRNATarget^37^. Second, for a given RNA pair (A and B), the miRNA regulators of the components were named set C and set D, respectively. The significance of the overlap between set C and set D was determined using a hypergeometric cumulative distribution function test (Equation 1), where *n* was the number of common miRNA regulators of the two RNAs, *U* was the size of the miRNAs’ universal set, and *M* and *N* were the sizes of miRNA sets C and D, respectively.


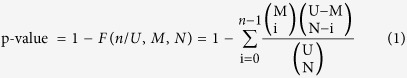


If the P-value of a RNA pair is less than 0.05, indicating that the miRNA sponges common to the two RNAs were significant, the two RNAs were identified as a candidate ceRNA pair. The expression levels’ tendencies of the RNAs in a ceRNA pair are expected to be similar^11^, therefore, the Spearman correlation coefficient was determined based on the RNA-seq data from all of the samples. Positively correlated pairs with *P*-values less than 0.05 were selected as the final ceRNA pairs and were incorporated into the ceRNA network.

### Network visualization and community detection

Cytoscape 3.2.0^38^ was used to analyze and visualize the ceRNA network. The communities were mined using the MCODE plugin^39^ for Cytoscape, with default parameters.

### Functional annotation of lincRNAs in rice undergoing Pi starvation

As described previously, ceRNA pairs competing for common miRNA sponges may have similar biological functions^11^,^40^; therefore, we used the ceRNA network to predict the functions of rice lincRNAs. Based on the hypothesis that RNAs with similar miRNA regulators can influence each other by competing for the limited number of miRNAs, a computational method described as above was applied to construct the ceRNA network.

In the networks, a community was defined as a cluster in which the nodes interacted with each other densely, whereas the interactions between its nodes and those outside of the community were sparse^41^,^42^. Communities within a biological network may work together as a functional unit^43^; therefore, a lincRNA in a dense cluster of the ceRNA network may have a similar function to that of the genes in the same cluster. The communities of the networks were identified using the MCODE tool^39^, and functional annotation of the communities was performed by enrichment analysis. If a lincRNA was not involved in a community, we defined its function as that of its direct neighbors, which was determined by enrichment analysis. If its neighbors were not enriched significantly, the function of the lincRNA was set as the GO term to which most of its neighbors belonged.

### Enrichment analysis

A hypergeometric cumulative distribution function test was used to identify the enriched GO terms for each community. Equation 1 was used; in this case, *n* represented the size of the intersection set between the community and the GO term, *U* represented the number of genes in the universal set, and *M* and *N* were the numbers of genes in the community and GO term, respectively. The GO terms with *P*-values less than 0.05 were selected as the enriched gene set for the community.

### Selection of hubs

In biological networks, nodes with higher degrees are more essential^44^,^45^,^46^,^47^; therefore, the top 20% of nodes with the highest degrees were selected as hubs.

### Identification of differentially expressed lincRNAs

EdgeR^48^ was used to identify lincRNAs that were differentially expressed between the Pi-starved and control samples at each time point. The lincRNAs with a false discovery rate less than 0.05 were selected as differentially expressed ones.

## Additional Information

**How to cite this article**: Xu, X.-W. *et al*. Functional analysis of long intergenic non-coding RNAs in phosphate-starved rice using competing endogenous RNA network. *Sci. Rep.* **6**, 20715; doi: 10.1038/srep20715 (2016).

## Supplementary Material

Supplementary Information

## Figures and Tables

**Table 1:** The Key LincRNAs in Root.

LincRNAs	Degree	GO ID	GO name
XLOC_026030	60	GO: 0016036	cellular response to phosphate starvation
XLOC_051315	51	GO: 0016036	cellular response to phosphate starvation
XLOC_010233	48	GO: 0016036	cellular response to phosphate starvation
XLOC_054628	28	GO: 0016036	cellular response to phosphate starvation
XLOC_049577	114	GO: 0009250	glucan biosynthetic process
XLOC_077203	100	GO: 0009250	glucan biosynthetic process
XLOC_055397	60	GO: 0009250	glucan biosynthetic process
XLOC_031943	59	GO: 0009250	glucan biosynthetic process
XLOC_062736	56	GO: 0009250	glucan biosynthetic process
XLOC_058915	52	GO: 0009250	glucan biosynthetic process
XLOC_027868	45	GO: 0009250	glucan biosynthetic process
XLOC_044456	33	GO: 0009250	glucan biosynthetic process
XLOC_003338	90	GO: 0047940	glucuronokinase activity
XLOC_018392	63	GO: 0047940	glucuronokinase activity
XLOC_058089	58	GO: 0047940	glucuronokinase activity
XLOC_027543	37	GO: 0047940	glucuronokinase activity
XLOC_037399	28	GO: 0047940	glucuronokinase activity
XLOC_014220	27	GO: 0047940	glucuronokinase activity
XLOC_041683	22	GO: 0047940	glucuronokinase activity
XLOC_018843	18	GO: 0070652	HAUS complex
XLOC_067881	18	GO: 0070652	HAUS complex
XLOC_020260	78	GO: 0006848	pyruvate transport
XLOC_055176	68	GO: 0006848	pyruvate transport
XLOC_030698	56	GO: 0006848	pyruvate transport
XLOC_032273	55	GO: 0006848	pyruvate transport
XLOC_067770	47	GO: 0006848	pyruvate transport
XLOC_026516	40	GO: 0006848	pyruvate transport
XLOC_040629	39	GO: 0006848	pyruvate transport
XLOC_025619	31	GO: 0090322	regulation of superoxide metabolic process
XLOC_059443	40	GO: 0002237	response to molecule of bacterial origin
XLOC_008468	26	GO: 0002237	response to molecule of bacterial origin
XLOC_001099	19	GO: 0002237	response to molecule of bacterial origin
XLOC_030020	40	GO: 0080150	S-adenosyl-L-methionine: benzoic acid carboxyl methyl transferase activity
XLOC_056226	33	GO: 0080150	S-adenosyl-L-methionine: benzoic acid carboxyl methyl transferase activity
XLOC_049097	28	GO: 0000124	SAGA complex
XLOC_001874	52	GO: 0005774	vacuolar membrane
XLOC_004428	45	–	–
XLOC_012843	31	–	–
XLOC_037613	27	–	–
XLOC_045461	26	–	–
XLOC_053440	26	–	–
XLOC_037810	25	–	–
XLOC_006373	21	–	–
XLOC_009491	20	–	–
XLOC_076889	20	–	–
XLOC_063523	19	–	–
XLOC_045455	18	–	–

**Table 2:** The Key LincRNAs in Shoot.

LincRNAs	Degree	GO ID	GO name
XLOC_029375	19	GO: 0000304	response to singlet oxygen
XLOC_071313	57	GO: 0080027	response to herbivore
XLOC_035144	43	GO: 0080027	response to herbivore
XLOC_073642	124	GO: 0009991	response to extracellular stimulus
XLOC_030020	83	GO: 0009991	response to extracellular stimulus
XLOC_059443	125	GO: 0009787	regulation of abscisic acid-activated signaling pathway
XLOC_027543	94	GO: 0009787	regulation of abscisic acid-activated signaling pathway
XLOC_045455	76	GO: 0009787	regulation of abscisic acid-activated signaling pathway
XLOC_043084	75	GO: 0009787	regulation of abscisic acid-activated signaling pathway
XLOC_074955	48	GO: 0009787	regulation of abscisic acid-activated signaling pathway
XLOC_037969	22	GO: 0006656	phosphatidylcholine biosynthetic process
XLOC_045400	32	GO: 0080148	negative regulation of response to water deprivation
XLOC_019886	241	GO: 0006378	mRNA polyadenylation
XLOC_053044	198	GO: 0006378	mRNA polyadenylation
XLOC_002113	173	GO: 0006378	mRNA polyadenylation
XLOC_068645	160	GO: 0006378	mRNA polyadenylation
XLOC_047346	143	GO: 0006378	mRNA polyadenylation
XLOC_031943	126	GO: 0006378	mRNA polyadenylation
XLOC_016946	110	GO: 0006378	mRNA polyadenylation
XLOC_069348	88	GO: 0006378	mRNA polyadenylation
XLOC_049097	65	GO: 0006378	mRNA polyadenylation
XLOC_058915	120	GO: 0006869	lipid transport
XLOC_030698	102	GO: 0006869	lipid transport
XLOC_024209	80	GO: 0006869	lipid transport
XLOC_077187	75	GO: 0006869	lipid transport
XLOC_026516	60	GO: 0006869	lipid transport
XLOC_010433	22	GO: 0008610	lipid biosynthetic process
XLOC_025812	60	GO: 0070652	HAUS complex
XLOC_054892	41	GO: 0070652	HAUS complex
XLOC_027447	27	GO: 0070652	HAUS complex
XLOC_076778	26	GO: 0070652	HAUS complex
XLOC_014220	34	GO: 0047940	glucuronokinase activity
XLOC_061908	23	GO: 0006680	glucosylceramide catabolic process
XLOC_048059	40	GO: 0017004	cytochrome complex assembly
XLOC_063523	144	GO: 0071368	cellular response to cytokinin stimulus
XLOC_040486	66	GO: 0071368	cellular response to cytokinin stimulus
XLOC_007198	48	–	–
XLOC_067119	26	–	–
XLOC_026094	25	–	–
XLOC_072903	19	–	–
