# Supplementary material for: Functional analysis of long intergenic non-coding RNAs in phosphate-starved rice using competing endogenous RNA network
Source: Sci Rep. 2016 Feb 10;6:20715. doi: 10.1038/srep20715 (PMC4748279; doi:10.1038/srep20715)
Supplement: Supplementary Information [file srep20715-s1.pdf]

Functional analysis of long intergenic non-coding RNAs in phosphate-starved rice using competing endogenous RNA network

Xi-Wen Xu[1, 2, *], Xiong-Hui Zhou[2, *], Rui-Ru Wang[1, 2], Wen-Lei Peng[1, 2], Yue An[1, 2] and Ling-Ling Chen[1, 2]

[1] National Key Laboratory of Crop Genetic Improvement, Huazhong Agricultural University, Wuhan 430070, P. R. China

[2] College of Informatics, Agricultural Bioinformatics Key Laboratory of Hubei Province, Huazhong Agricultural University, Wuhan 430070, P. R. China

[*]These authors contributed equally to this work.

Correspondence and requests for materials should be addressed to: L.-L. C. (email: llchen@mail.hzau.edu.cn)

Supplementary Information

Table S1. Annotations of the Clusters in the Rice Root CeRNA Network.

Table S2. Annotations of the Clusters in the Rice Shoot CeRNA Network.

Table S3. Annotations of the LincRNAs in Rice Root.

Table S4. Annotations of the LincRNAs in Rice Shoot.

Table S1. Annotations of the Clusters in the Rice Root CeRNA Network

Cluster ID	Nodes in the cluster	GO ID	GO name	GO description	***P*-value**
104	Os01g0842400,Os01g0842500,Os05g0458300,Os05g0458600,	GO:0052716	hydroquinone:oxygen oxidoreductase activity	Catalysis of the reaction: 4 hydroquinone + O2 = 4 benzosemiquinone + 4 H2O. [EC:1.10.3.2]	9.44467E-13
35	Os01g0832900,Os03g0610900,Os05g0489900,Os06g0606000,Os07g0194100,Os09g0514200,Os10g0100500,Os12g0586100,	GO:0004674	protein serine/threonine kinase activity	Catalysis of the reactions: ATP + protein serine = ADP + protein serine phosphate, and ATP + protein threonine = ADP + protein threonine phosphate. [GOC:bf]	4.98049E-11
17	Os01g0149400,Os01g0149600,Os01g0153300,Os01g0839500,Os02g0820400,Os05g0574300,Os08g0490900,Os11g0209100,Os12g0270900,Os12g0549700,	GO:0046982	protein heterodimerization activity	Interacting selectively and non-covalently with a nonidentical protein to form a heterodimer. [GOC:ai]	7.37029E-10
64	Os02g0776400,Os03g0174900,Os03g0647600,Os03g0696300,Os07g0608200,	GO:0016602	CCAAT-binding factor complex	A heteromeric transcription factor complex that binds to the CCAAT-box upstream of promoters; in Saccharomyces it activates the transcription of genes in response to growth in a nonfermentable carbon source; consists of four known subunits: HAP2, HAP3, HAP4 and HAP5. [PMID:7828851]	3.1965E-09
209	Os01g0600000,Os05g0533800,Os07g0203300,	GO:0000276	mitochondrial proton-transporting ATP synthase complex, coupling factor F(o)	All non-F1 subunits of the mitochondrial hydrogen-transporting ATP synthase, including integral and peripheral mitochondrial inner membrane proteins. [GOC:mtg_sensu, PMID:10838056]	5.84E-08
32	Os02g0254700,Os02g0532500,Os03g0274700,Os08g0528601,Os09g0378300,Os09g0503400,XLOC_015851,	GO:0004823	leucine-tRNA ligase activity	Catalysis of the reaction: L-leucine + ATP + tRNA(Leu) = AMP + diphosphate + 2 H(+) + Leu-tRNA(Leu). [EC:6.1.1.4, RHEA:11691]	1.45992E-07
187	Os01g0639100,Os03g0566800,Os08g0323266,	GO:0035145	exon-exon junction complex	A multi-subunit complex deposited by the spliceosome upstream of messenger RNA exon-exon junctions. The exon-exon junction complex provides a binding platform for factors involved in mRNA export and nonsense-mediated mRNA decay. [PMID:11532962, PMID:11743026]	1.45992E-07
203	Os05g0496400,Os05g0496500,Os08g0109800,	GO:0004197	cysteine-type endopeptidase activity	Catalysis of the hydrolysis of internal, alpha-peptide bonds in a polypeptide chain by a mechanism in which the sulfhydryl group of a cysteine residue at the active center acts as a nucleophile. [GOC:mah, http://merops.sanger.ac.uk/about/glossary.htm#CATTYPE, http://merops.sanger.ac.uk/about/glossary.htm#ENDOPEPTIDASE]	2.24731E-06
43	Os01g0670100,Os03g0764800,Os04g0486500,Os06g0291600,Os07g0678600,Os11g0168100,Os12g0527700,	GO:0004674	protein serine/threonine kinase activity	Catalysis of the reactions: ATP + protein serine = ADP + protein serine phosphate, and ATP + protein threonine = ADP + protein threonine phosphate. [GOC:bf]	7.03664E-06
75	Os01g0355600,Os03g0729500,Os06g0130200,Os07g0681500,Os12g0484900,	GO:0016818	hydrolase activity, acting on acid anhydrides, in phosphorus-containing anhydrides	Catalysis of the hydrolysis of any acid anhydride which contains phosphorus. [GOC:jl]	1.40823E-05
57	Os02g0788400,Os05g0474700,Os09g0245500,Os09g0551300,Os11g0580000,	GO:0022618	ribonucleoprotein complex assembly	The aggregation, arrangement and bonding together of proteins and RNA molecules to form a ribonucleoprotein complex. [GOC:jl]	2.92023E-05
3	Os01g0194200,Os01g0220300,Os01g0516600,Os01g0919800,Os02g0174000,Os02g0440000,Os02g0541325,Os02g0557800,Os02g0564700,Os03g0127500,Os03g0129900,Os03g0203800,Os03g0324900,Os03g0633800,Os03g0756400,Os03g0805200,Os03g0810500,Os03g0858800,Os04g0381700,Os04g0450300,Os05g0150733,Os05g0163300,Os05g0223000,Os05g0428400,Os05g0532600,Os06g0316300,Os06g0506000,Os06g0543200,Os06g0678100,Os06g0680900,Os06g0724100,Os06g0724200,Os07g0198300,Os07g0202100,Os07g0230500,Os07g0406800,Os07g0464600,Os07g0641200,Os08g0170700,Os08g0559000,Os09g0488800,Os10g0442532,Os10g0577900,Os11g0148200,Os11g0149300,Os11g0534300,Os11g0644800,Os12g0115500,Os12g0169700,Os12g0569900,Os12g0583500,	GO:0031966	mitochondrial membrane	Either of the lipid bilayers that surround the mitochondrion and form the mitochondrial envelope. [GOC:mah]	3.18777E-05
11	Os01g0147250,Os01g0264400,Os01g0316100,Os01g0757600,Os01g0888300,Os01g0909400,Os01g0972800,Os02g0146700,Os02g0198400,Os02g0224200,Os02g0587800,Os02g0672600,Os02g0755000,Os03g0169900,Os03g0238300,Os03g0314100,Os03g0697200,Os04g0405500,Os04g0533000,Os04g0602900,Os04g0606000,Os05g0304200,Os05g0429900,Os05g0589600,Os06g0193200,Os06g0220200,Os06g0233200,Os06g0677700,Os06g0715000,Os07g0231800,Os08g0112300,Os09g0482100,Os09g0482600,Os10g0390000,Os11g0127800,Os11g0546900,Os11g0600700,Os12g0165900,	GO:0071277	cellular response to calcium ion	Any process that results in a change in state or activity of a cell (in terms of movement, secretion, enzyme production, gene expression, etc.) as a result of a calcium ion stimulus. [GOC:mah]	4.76693E-05
45	Os01g0839900,Os02g0580500,Os07g0164500,Os07g0197500,Os09g0571100,Os12g0574700,	GO:0042545	cell wall modification	The series of events leading to chemical and structural alterations of an existing cell wall that can result in loosening, increased extensibility or disassembly. [GOC:jl]	7.99677E-05

165	Os04g0682900,Os07g0521600,XLOC_032300,	GO:0000404	loop DNA binding	Interacting selectively and non-covalently with DNA containing a loop. A loop occurs when DNA contains a large insertion or deletion that causes a region of unpaired single-stranded DNA to loop out, while the rest of the DNA is in a paired double-stranded configuration. [GOC:elh, PMID:16781730]	8.05542E-05
175	Os01g0814800,Os02g0511901,XLOC_025619,	GO:0090322	regulation of superoxide metabolic process	Any process that modulates the rate, frequency, or extent of superoxide metabolism, the chemical reactions and pathways involving superoxide, the superoxide anion O2- (superoxide free radical), or any compound containing this species. [GOC:tb]	8.05542E-05
2	Os01g0104800,Os01g0896800,Os02g0102400,Os02g0184100,Os02g0231600,Os02g0512400,Os02g0576700,Os02g0699500,Os03g0312600,Os03g0333100,Os03g0336300,Os03g0572250,Os03g0598200,Os03g0668900,Os03g0694900,Os03g0825600,Os03g0832200,Os03g0843300,Os04g0337300,Os04g0459700,Os05g0232700,Os05g0255600,Os05g0301600,Os05g0373700,Os05g0539700,Os05g0597100,Os06g0134300,Os07g0124500,Os07g0201100,Os07g0246200,Os07g0270900,Os07g0475800,Os07g0603200,Os07g0611600,Os07g0614400,Os07g0623300,Os07g0639400,Os07g0640000,Os08g0439900,Os08g0494000,Os08g0500700,Os09g0103400,Os09g0241100,Os10g0447600,Os11g0119900,Os11g0130300,Os11g0644700,Os12g0197500,Os12g0498800,Os12g0576300,XLOC_059805,	GO:0005829	cytosol	The part of the cytoplasm that does not contain organelles but which does contain other particulate matter, such as protein complexes. [GOC:hgd, GOC:jl]	0.000105095
129	Os01g0187600,Os02g0566800,Os03g0144800,Os06g0105700,	GO:0006486	protein glycosylation	A protein modification process that results in the addition of a carbohydrate or carbohydrate derivative unit to a protein amino acid, e.g. the addition of glycan chains to proteins. [GOC:curators, GOC:pr]	0.000105707
8	Os01g0611100,Os02g0192700,Os02g0315400,Os02g0713400,Os03g0344650,Os03g0370500,Os03g0562200,Os05g0135000,Os05g0200340,Os06g0320500,Os06g0348800,Os07g0195350,Os07g0239400,Os07g0636000,Os11g0482000,Os12g0508266,Os12g0583300,	GO:0009982	pseudouridine synthase activity	Catalysis of the reaction: RNA uridine = RNA pseudouridine. Conversion of uridine in an RNA molecule to pseudouridine by rotation of the C1'-N-1 glycosidic bond of uridine in RNA to a C1'-C5. [EC:5.4.99.12, GOC:mah]	0.000110701
94	Os01g0747500,Os04g0550500,Os09g0454300,XLOC_040881,	GO:0004151	dihydroorotase activity	Catalysis of the reaction: (S)-dihydroorotate + H(2)O = N-carbamoyl-L-aspartate + H(+). [EC:3.5.2.3, RHEA:24299]	0.000120831
127	Os01g0182900,Os03g0743900,Os08g0450800,XLOC_026644,	GO:0070206	protein trimerization	The formation of a protein trimer, a macromolecular structure consisting of three noncovalently associated identical or nonidentical subunits. [GOC:hjd]	0.000120831
134	Os01g0155500,Os01g0170051,Os12g0443000,	GO:0048480	stigma development	The process whose specific outcome is the progression of the stigma over time, from its formation to the mature structure. The stigma is the pollen-receptive surface of a carpel or group of fused carpels, usually sticky. [GOC:jid, PO:0009073]	0.000120831
138	Os01g0947833,Os03g0591300,Os04g0484900,	GO:0010084	specification of organ axis polarity	The process in which the polarity of an organ axis is specified. [GOC:tb]	0.000120831
151	Os01g0519400,Os07g0509600,Os09g0515400,	GO:0046085	adenosine metabolic process	The chemical reactions and pathways involving adenosine, adenine riboside, a ribonucleoside found widely distributed in cells of every type as the free nucleoside and in combination in nucleic acids and various nucleoside coenzymes. [GOC:go_curators]	0.000120831
163	Os03g0659266,Os10g0456500,Os11g0275500,	GO:0043015	gamma-tubulin binding	Interacting selectively and non-covalently with the microtubule constituent protein gamma-tubulin. [GOC:jl]	0.000120831
179	Os02g0722800,Os03g0681300,Os04g0563801,	GO:0008158	hedgehog receptor activity	Combining with a member of the hedgehog protein family and transmitting the signal across the membrane to initiate a change in cell activity. [GOC:bf, GOC:go_curators, PMID:9278137]	0.000120831
219	Os01g0558500,Os02g0611800,Os11g0139900,	GO:0047205	quinate O-hydroxycinnamoyltransferase activity	Catalysis of the reaction: feruloyl-CoA + quinate = O-feruloylquinate + CoA. [EC:2.3.1.99, MetaCyc:2.3.1.99-RXN]	0.000120831
88	Os03g0111500,Os04g0556000,Os07g0600400,Os09g0476000,	GO:0010273	detoxification of copper ion	Any process that reduces or removes the toxicity of copper ion. These include transport of copper away from sensitive areas and to compartments or complexes whose purpose is sequestration of copper ion. [GOC:kmv, PMID:16367966]	0.000161108
108	Os04g0490700,Os06g0670000,Os12g0270200,Os12g0623500,	GO:0008265	Mo-molybdopterin cofactor sulfurase activity	Catalysis of the sulfurylation of the desulfo form of molybdenum cofactor (MoCo), a cofactor required for the activity of some enzymes, such as aldehyde oxidase. [GOC:mah, PMID:11549764]	0.000161108

59	Os03g0759700,Os03g0765200,Os07g0512200,Os09g0460000,Os11g0549700,	GO:0005776	autophagic vacuole	A double-membrane-bounded compartment in which endogenous cellular material is sequestered; known as autophagosome in yeast. [ISBN:0198547684, PMID:11099404]	0.000201386
69	Os01g0765000,Os05g0453900,Os07g0164100,Os08g0500900,Os11g0309000,	GO:0000811	GINS complex	A heterotetrameric protein complex that associates with replication origins, where it is required for the initiation of DNA replication, and with replication forks. [GOC:rb, GOC:rn, PMID:12730134, PMID:16990792, PMID:17467990]	0.000201386
73	Os01g0597800,Os06g0185500,Os08g0459700,Os09g0532900,Os11g0138400,	GO:0010294	abscisic acid glucosyltransferase activity	Catalysis of the reaction: (+)-abscisate + UDP-D-glucose = abscisic acid glucose ester + UDP. [DOI:10.1016/j.tetasy.2004.11.062]	0.000201386
80	Os01g0130900,Os02g0121100,Os03g0395801,Os07g0495100,Os12g0240000,	GO:0043457	regulation of cellular respiration	Any process that modulates the frequency, rate or extent of cellular respiration, the enzymatic release of energy from organic compounds. [GOC:jl]	0.000201386
93	Os01g0222500,Os01g0927000,Os03g0702700,Os04g0413600,Os04g0658700,	GO:0046974	histone methyltransferase activity (H3-K9 specific)	Catalysis of the reaction: S-adenosyl-L-methionine + histone H3 L-lysine (position 9) = S-adenosyl-L-homocysteine + histone H3 N6-methyl-L-lysine (position 9). This reaction is the addition of a methyl group onto lysine at position 9 of the histone H3 protein. [GOC:ai]	0.000201386
113	Os02g0711400,Os04g0429450,Os04g0506800,Os07g0176500,Os07g0249700,	GO:0010210	IAA-Phe conjugate hydrolase activity	Catalysis of the reaction: indole-3-acetyl-phenylalanine + H2O = indole-3-acetate + phenylalanine. [GOC:syr]	0.000201386
160	Os02g0679200,Os03g0279500,Os07g0686500,Os09g0544900,Os12g0609200,XLOC_022758,XLOC_043736,	GO:0033542	fatty acid beta-oxidation, unsaturated, even number	A fatty acid beta-oxidation pathway by which fatty acids having cis-double bonds on even-numbered carbons are degraded. Fatty acid beta-oxidation begins with the addition of coenzyme A to a fatty acid, and ends when only two or three carbons remain (as acetyl-CoA or propionyl-CoA respectively). [GOC:mah, MetaCyc:PWY-5138]	0.000201386
70	Os02g0315600,Os06g0625500,Os08g0156800,Os09g0413500,Os11g0595100,	GO:0022625	cytosolic large ribosomal subunit	The large subunit of a ribosome located in the cytosol. [GOC:mtg_sensu]	0.000207107
116	Os01g0583100,Os01g0859400,Os02g0453300,Os05g0119000,Os11g0685500,	GO:0006470	protein dephosphorylation	The process of removing one or more phosphoric residues from a protein. [GOC:hb]	0.000229471
131	Os01g0279700,Os01g0752200,Os01g0851100,XLOC_016325,	GO:0003860	3-hydroxyisobutyryl-CoA hydrolase activity	Catalysis of the reaction: 3-hydroxy-2-methylpropanoyl-CoA + H2O = CoA + 3-hydroxy-2-methylpropanoate. [EC:3.1.2.4]	0.000241653
136	Os05g0319100,Os11g0455500,Os11g0525200,	GO:0004013	adenosylhomocysteinase activity	Catalysis of the reaction: S-adenosyl-L-homocysteine + H2O = adenosine + L-homocysteine. [EC:3.3.1.1]	0.000241653
159	Os01g0543400,Os02g0511100,Os05g0580000,	GO:0010170	glucose-1-phosphate adenylyltransferase complex	Complex that catalyzes the synthesis of ADP-glucose and pyrophosphate from glucose-1-phosphate and ATP. In plants, the complex is a heterotetramer composed of two types of subunits (small and large). In bacteria, the enzyme complex is composed of four identical subunits. [GOC:tb, PMID:12748181]	0.000241653
164	Os04g0398800,Os06g0183100,Os08g0562100,	GO:0046554	malate dehydrogenase (NADP+) activity	Catalysis of the reaction: (S)-malate + NADP+ = oxaloacetate + NADPH + H+. [EC:1.1.1.82]	0.000241653
168	Os01g0795400,Os01g0899500,Os08g0544400,	GO:0071366	cellular response to indolebutyric acid stimulus	Any process that results in a change in state or activity of a cell (in terms of movement, secretion, enzyme production, gene expression, etc.) as a result of an indolebutyric acid stimulus. [GOC:mah]	0.000241653
171	Os03g0273800,Os04g0662900,Os07g0679300,	GO:0016480	negative regulation of transcription from RNA polymerase III promoter	Any process that stops, prevents, or reduces the frequency, rate or extent of transcription from an RNA polymerase III promoter. [GOC:go_curators]	0.000241653

189	Os01g0741900,Os04g0416100,Os05g0129000,	GO:0004357	glutamate-cysteine ligase activity	Catalysis of the reaction: L-cysteine + L-glutamate + ATP = L-gamma-glutamyl-L-cysteine + ADP + 2 H(+) + phosphate. [EC:6.3.2.2, RHEA:13288]	0.000241653
194	Os05g0496000,Os07g0637300,Os08g0540400,	GO:0004740	pyruvate dehydrogenase (acetyl-transferring) kinase activity	Catalysis of the reaction: ATP + pyruvate dehydrogenase (acetyl-transferring) = ADP + pyruvate dehydrogenase (acetyl-transferring) phosphate. [EC:2.7.11.2]	0.000241653
217	Os01g0827300,Os07g0569800,Os11g0158400,	GO:0046480	galactolipid galactosyltransferase activity	Catalysis of the reaction: 2 mono-beta-D-galactosyldiacylglycerol = alpha-D-galactosyl-beta-D-galactosyldiacylglycerol + 1,2-diacylglycerol. [EC:2.4.1.184]	0.000241653
47	Os01g0207200,Os02g0643500,Os02g0657600,Os07g0636900,Os08g0342300,Os12g0186600,	GO:0034618	arginine binding	Interacting selectively and non-covalently with 2-amino-5-(carbamimidamido)pentanoic acid. [CHEBI:29016, GOC:BHF, GOC:rl]	0.000241663
49	Os02g0717400,Os03g0243300,Os03g0385900,Os06g0667500,Os08g0386300,Os09g0440300,	GO:0005080	protein kinase C binding	Interacting selectively and non-covalently with protein kinase C. [GOC:jl]	0.000241663
55	Os03g0170800,Os03g0301600,Os03g0645000,Os04g0276600,Os04g0428950,Os06g0671800,	GO:0080142	regulation of salicylic acid biosynthetic process	Any process that modulates the frequency, rate or extent of the chemical reactions and pathways resulting in the formation of salicylic acid. [GOC:dhl]	0.000241663
66	Os01g0355500,Os02g0817700,Os04g0661100,Os06g0633100,Os07g0180800,Os07g0529600,	GO:0003988	acetyl-CoA C-acyltransferase activity	Catalysis of the reaction: acyl-CoA + acetyl-CoA = CoA + 3-oxoacyl-CoA. [EC:2.3.1.16]	0.000241663
22	Os04g0337201,Os06g0146800,Os10g0210500,Os10g0496900,Os11g0202600,Os11g0545000,Os12g0486800,XLOC_023020,	GO:0032973	amino acid export	The directed movement of amino acids out of a cell or organelle. [GOC:mah]	0.00028194
36	Os01g0686100,Os01g0694100,Os02g0252600,Os03g0850200,Os07g0185700,Os10g0485300,Os12g0210200,XLOC_018843,XLOC_033922,XLOC_060416,XLOC_067881,	GO:0070652	HAUS complex	A protein complex that localizes to interphase centrosomes and to mitotic spindle tubules and regulates mitotic spindle assembly and centrosome integrity; in human, the complex consists of eight subunits, some of which are homologous to subunits of the Drosophila Augmin complex. [PMID:19427217]	0.00028194
37	Os01g0376600,Os05g0594900,Os07g0611400,Os08g0133600,Os10g0467600,Os11g0621400,Os12g0194600,	GO:0000340	RNA 7-methylguanosine cap binding	Interacting selectively and non-covalently with the 7-methylguanosine group added cotranscriptionally to the 5' end of RNA molecules transcribed by polymerase II. [GOC:krc]	0.00028194
106	Os06g0255700,Os07g0434500,Os09g0436500,Os12g0131000,	GO:0003676	nucleic acid binding	Interacting selectively and non-covalently with any nucleic acid. [GOC:jl]	0.000297169
119	Os03g0265400,Os03g0775500,Os06g0724500,Os09g0356100,	GO:0009547	plastid ribosome	A ribosome contained within a plastid. [GOC:tair_curators]	0.000322197
221	Os01g0952600,Os02g0121300,Os02g0503100,Os02g0638400,	GO:0001932	regulation of protein phosphorylation	Any process that modulates the frequency, rate or extent of addition of phosphate groups into an amino acid in a protein. [GOC:hjd]	0.000322197
21	Os01g0766966,Os02g0753300,Os03g0156500,Os03g0313000,Os03g0819900,Os04g0402700,Os07g0211601,Os08g0135400,	GO:0022904	respiratory electron transport chain	A process in which a series of electron carriers operate together to transfer electrons from donors such as NADH and FADH2 to any of several different terminal electron acceptors to generate a transmembrane electrochemical gradient. [GOC:mtg_electron_transport, ISBN:0716720094]	0.000322217
30	Os01g0107400,Os04g0430700,Os04g0555800,Os08g0158500,Os08g0243600,Os08g0455600,Os10g0418000,Os11g0608700,	GO:0006114	glycerol biosynthetic process	The chemical reactions and pathways resulting in the formation of glycerol, 1,2,3-propanetriol, a sweet, hygroscopic, viscous liquid, widely distributed in nature as a constituent of many lipids. [GOC:ai, ISBN:0198506732]	0.000322217

76	Os01g0625200,Os02g0280200,Os02g0743100,Os03g0330000,Os04g0625000,Os09g0323700,Os10g0454200,Os12g0548100,	GO:0000799	nuclear condensin complex	A multisubunit protein complex that plays a central role in the condensation of chromosomes that remain in the nucleus. [GOC:elh]	0.000322217
77	Os01g0125700,Os01g0314000,Os03g0214900,Os03g0853700,Os04g0483600,Os04g0613700,Os04g0635900,Os04g0636800,Os04g0691200,Os05g0151000,Os06g0602500,Os06g0694100,Os07g0168000,Os07g0583700,Os07g0617000,Os11g0557400,Os11g0660500,	GO:0090305	nucleic acid phosphodiester bond hydrolysis	The nucleic acid metabolic process in which the phosphodiester bonds between nucleotides are cleaved by hydrolysis. [GOC:dph, GOC:tb]	0.000356201
141	Os01g0103400,Os04g0398500,Os08g0518200,	GO:0016679	oxidoreductase activity, acting on diphenols and related substances as donors	Catalysis of an oxidation-reduction (redox) reaction in which a diphenol or related substance acts as a hydrogen or electron donor and reduces a hydrogen or electron acceptor. [GOC:ai]	0.000362465
181	Os01g0945001,Os02g0747400,Os08g0441400,	GO:0000002	mitochondrial genome maintenance	The maintenance of the structure and integrity of the mitochondrial genome; includes replication and segregation of the mitochondrial chromosome. [GOC:ai, GOC:vw]	0.000362465
186	Os02g0226900,Os02g0545000,Os04g0512900,	GO:0017137	Rab GTPase binding	Interacting selectively and non-covalently with Rab protein, any member of the Rab subfamily of the Ras superfamily of monomeric GTPases. [GOC:mah]	0.000362465
190	Os03g0266900,Os03g0641300,Os05g0583075,	GO:0045471	response to ethanol	Any process that results in a change in state or activity of a cell or an organism (in terms of movement, secretion, enzyme production, gene expression, etc.) as a result of an ethanol stimulus. [GOC:go_curators]	0.000362465
197	Os01g0111500,Os06g0266400,Os07g0628600,	GO:0005664	nuclear origin of replication recognition complex	A multisubunit complex that is located at the replication origins of a chromosome in the nucleus. [GOC:elh]	0.000362465
198	Os04g0490500,Os07g0282300,Os12g0607000,	GO:0033528	S-methylmethionine cycle	A cyclic series of interconversions involving S-methyl-L-methionine, S-adenosyl-L-homocysteine, S-adenosyl-L-methionine, L-homocysteine, and L-methionine. Converts the methionine group of adenosylmethionine back to free methionine, and may serve regulate the cellular adenosylmethionine level. [GOC:mah, MetaCyc:PWY-5441]	0.000362465
33	Os01g0500900,Os01g0588100,Os01g0924300,Os01g0950200,Os02g0106300,Os02g0734300,Os08g0159800,Os08g0548200,Os11g0102600,	GO:0006437	tyrosyl-tRNA aminoacylation	The process of coupling tyrosine to tyrosyl-tRNA, catalyzed by tyrosyl-tRNA synthetase. In tRNA aminoacylation, the amino acid is first activated by linkage to AMP and then transferred to either the 2'- or the 3'-hydroxyl group of the 3'-adenosine residue of the tRNA. [GOC:mcc, ISBN:0716730510]	0.000362494
61	Os01g0905200,Os02g0747900,Os03g0150700,Os04g0585200,Os08g0430800,Os10g0464500,Os10g0517500,Os11g0207000,Os12g0456100,	GO:0018826	methionine gamma-lyase activity	Catalysis of the reaction: L-methionine = methanethiol + NH3 + 2-oxobutanoate. [EC:4.4.1.11]	0.000362494
110	Os01g0121200,Os01g0390600,Os02g0677800,Os03g0712900,Os04g0466600,Os06g0520600,Os07g0501800,Os09g0507100,Os11g0621300,	GO:0004668	protein-arginine deiminase activity	Catalysis of the reaction: protein L-arginine + H2O = protein L-citrulline + NH3. [EC:3.5.3.15]	0.000362494
60	Os01g0948400,Os02g0720700,Os03g0200700,Os07g0204000,Os12g0588800,	GO:0004735	pyrroline-5-carboxylate reductase activity	Catalysis of the reaction: L-proline + NADP+ = 1-pyrroline-5-carboxylate + NADPH + H+. [EC:1.5.1.2]	0.000402739
62	Os02g0180500,Os02g0802301,Os04g0469800,Os07g0116800,Os08g0182400,	GO:0010012	steroid 22-alpha hydroxylase activity	Catalysis of the reaction: 5-alpha-campestanaol + O2 = 6-deoxocathasterone + H2O. [GOC:tb]	0.000402739
68	Os02g0258250,Os03g0101500,Os03g0802900,Os06g0691200,Os12g0417100,	GO:0048446	petal morphogenesis	The process in which the anatomical structures of the petal are generated and organized. [GOC:go_curators]	0.000402739
20	Os01g0176500,Os01g0212900,Os01g0265200,Os01g0757500,Os02g0287000,Os02g0790600,Os02g0829900,Os03g0195100,Os03g0770900,Os11g0236932,	GO:0007163	establishment or maintenance of cell polarity	Any cellular process that results in the specification, formation or maintenance of anisotropic intracellular organization or cell growth patterns. [GOC:mah]	0.000402771

9	Os01g0107000,Os01g0135900,Os01g0645900,Os01g0695800,Os01g0710250,Os01g0752100,Os01g0825500,Os01g0846300,Os01g0852100,Os01g0949700,Os01g0975300,Os02g0119700,Os02g0122200,Os02g0202800,Os02g0293800,Os03g0145500,Os03g0244466,Os03g0348800,Os03g0644000,Os03g0683900,Os03g0734000,Os03g0837000,Os04g0401300,Os04g0418100,Os04g0580800,Os05g0126700,Os05g0161200,Os05g0172100,Os05g0512200,Os05g0522600,Os05g0568100,Os06g0160700,Os06g0271900,Os06g0602950,Os06g0690200,Os06g0691400,Os07g0412100,Os07g0647800,Os07g0668300,Os08g0451400,Os08g0480400,Os08g0537900,Os09g0250700,Os09g0498000,Os09g0506900,Os09g0533650,Os10g0135250,Os10g0442100,Os10g0470900,Os10g0516800,Os11g0141400,Os11g0151800,Os11g0158832,Os12g0107500,Os12g0137000,Os12g0138050,Os12g0151000,Os12g0197400,Os12g0570075,XLOC_008741,XLOC_019493,XLOC_024889,XLOC_027868,XLOC_031943,XLOC_033910,XLOC_044456,XLOC_049577,XLOC_053506,XLOC_055397,XLOC_058915,XLOC_061152,XLOC_062736,XLOC_077203,	GO:0009250	glucan biosynthetic process	The chemical reactions and pathways resulting in the formation of glucans, polysaccharides consisting only of glucose residues. [GOC:go_curators]	0.000425791
24	Os01g0127100,Os01g0127200,Os01g0248701,Os01g0358300,Os01g0629400,Os01g0786900,Os01g0897700,Os01g0920300,Os01g0946700,Os02g0136000,Os02g0170000,Os02g0176100,Os02g0182700,Os02g0224100,Os02g0625300,Os02g0649900,Os02g0786200,Os03g0115700,Os03g0327100,Os03g0339900,Os03g0701000,Os04g0175600,Os04g0432250,Os04g0521900,Os05g0137400,Os05g0406100,Os05g0466800,Os06g0147100,Os06g0210500,Os06g0661766,Os06g0710700,Os07g0178600,Os07g0581100,Os08g0460800,Os08g0558800,Os09g0244900,Os09g0514400,Os10g0151800,Os10g0160600,Os10g0365050,Os10g0390800,Os10g0477600,Os11g0123400,Os12g0121300,XLOC_009323,XLOC_010233,XLOC_026030,XLOC_026206,XLOC_036449,XLOC_051315,XLOC_054628,	GO:0016036	cellular response to phosphate starvation	Any process that results in a change in state or activity of a cell (in terms of movement, secretion, enzyme production, gene expression, etc.) as a result of deprivation of phosphate. [GOC:jl]	0.000463421
83	Os01g0546500,Os01g0810000,Os09g0504400,Os09g0553900,	GO:0016591	DNA-directed RNA polymerase II, holoenzyme	Large protein complex composed of the RNA polymerase core complex and a variety of other proteins including transcription factor complexes TFIIA, D, E, F, and H which are required for promoter recognition, and the Mediator subcomplex. Catalyzes the synthesis of eukaryotic pre-mRNA. [GOC:jl, PMID:15196470, Wikipedia:Rna_polymerase_ii]	0.000483267
89	Os01g0698000,Os03g0379300,Os04g0560400,Os12g0140200,	GO:0006516	glycoprotein catabolic process	The chemical reactions and pathways resulting in the breakdown of glycoproteins, any protein that contains covalently bound glycose (i.e. monosaccharide) residues; the glycose occurs most commonly as oligosaccharide or fairly small polysaccharide but occasionally as monosaccharide. [GOC:go_curators, ISBN:0198506732]	0.000483267
128	Os01g0866600,Os02g0195600,Os03g0750100,Os05g0182000,	GO:0051879	Hsp90 protein binding	Interacting selectively and non-covalently with Hsp90 proteins, any of a group of heat shock proteins around 90kDa in size. [GOC:ai]	0.000483267
167	Os01g0641100,Os07g0688800,Os09g0500600,	GO:0003917	DNA topoisomerase type I activity	Catalysis of a DNA topological transformation by transiently cleaving one DNA strand at a time to allow passage of another strand; changes the linking number by +1 per catalytic cycle. [PMID:8811192]	0.000483267
213	Os03g0210600,Os03g0218300,Os10g0565401,	GO:0090421	embryonic meristem initiation	Initiation of a region of tissue in a plant embryo that is composed of one or more undifferentiated cells capable of undergoing mitosis and differentiation. [GOC:tb]	0.000483267
215	Os01g0263000,Os03g0390400,Os06g0127000,	GO:0044375	regulation of peroxisome size	Any process that modulates the volume of a peroxisome, a small, membrane-bounded organelle that uses dioxygen (O2) to oxidize organic molecules. [GOC:jl]	0.000483267
54	Os01g0667200,Os02g0255500,Os03g0217900,Os05g0179800,Os05g0393200,Os11g0139600,	GO:0050313	sulfur dioxygenase activity	Catalysis of the reaction: sulfur + O2 + H2O = sulfite. [EC:1.13.11.18, MetaCyc:SULFUR-DIOXYGENASE-RXN]	0.000483277
225	Os02g0238500,Os03g0363600,Os04g0664800,Os05g0534500,Os07g0498400,Os07g0531400,Os07g0695400,Os09g0511900,Os09g0530750,Os09g0567201,Os11g0459600,Os11g0501500,Os12g0566050,XLOC_020078,XLOC_037259,XLOC_040499,	GO:0008544	epidermis development	The process whose specific outcome is the progression of the epidermis over time, from its formation to the mature structure. The epidermis is the outer epithelial layer of a plant or animal, it may be a single layer that produces an extracellular material (e.g. the cuticle of arthropods) or a complex stratified squamous epithelium, as in the case of many vertebrate species. [GOC:go_curators, UBERON:0001003]	0.000523602
51	Os06g0622500,Os07g0409100,Os07g0613300,Os08g0127300,Os11g0272800,Os11g0579400,Os12g0539333,	GO:0015932	nucleobase-containing compound transmembrane transporter activity	Catalysis of the transfer of nucleobases, nucleosides, nucleotides and nucleic acids from one side of a membrane to the other. [GOC:ai]	0.000563811
52	Os02g0267700,Os03g0389601,Os05g0426066,Os05g0433400,Os07g0211200,Os10g0536600,Os12g0603700,	GO:2000762	regulation of phenylpropanoid metabolic process	Any process that modulates the frequency, rate or extent of phenylpropanoid metabolic process. [GOC:obol]	0.000563811
53	Os01g0247700,Os01g0680200,Os02g0176000,Os03g0275100,Os03g0355700,Os03g0433200,Os05g0573800,Os06g0116800,Os06g0573600,Os06g0638500,Os07g0638500,Os07g0666300,Os09g0461500,Os11g0642400,	GO:0042349	guiding stereospecific synthesis activity	The orientation of free radical substrates in such a way that only a particular stereoisomer is synthesized by an enzyme. Best characterized as a function during lignan biosynthesis. [GOC:ma]	0.000563879

174	Os01g0813400,Os01g0930400,Os03g0323200,	GO:0016851	magnesium chelatase activity	Catalysis of the reaction: ATP + H(2)O + Mg(2+) + protoporphyrin IX = ADP + 2 H(+) + magnesium protoporphyrin IX + phosphate. [EC:6.6.1.1, RHEA:13964]	0.000604059
178	Os02g0598400,Os04g0591232,Os09g0419200,	GO:0016621	cinnamoyl-CoA reductase activity	Catalysis of the reaction: cinnamaldehyde + CoA + NADP+ = cinnamoyl-CoA + NADPH + H+. [EC:1.2.1.44]	0.000604059
207	Os04g0177300,Os07g0573700,Os10g0394700,	GO:0005338	nucleotide-sugar transmembrane transporter activity	Catalysis of the transfer of a nucleotide-sugar from one side of the membrane to the other. A nucleotide-sugar is any nucleotide in which the distal phosphoric residue of a nucleoside 5'-diphosphate is in glycosidic linkage with a monosaccharide or monosaccharide derivative. [GOC:ai, GOC:mtg_transport, ISBN:0815340729, PMID:15034926]	0.000604059
211	Os01g0796000,Os01g0834700,Os02g0608600,	GO:0003984	acetolactate synthase activity	Catalysis of the reaction: 2 pyruvate = 2-acetolactate + CO2. [EC:2.2.1.6]	0.000604059
212	Os02g0221600,Os03g0732100,Os12g0479400,	GO:0008285	negative regulation of cell proliferation	Any process that stops, prevents or reduces the rate or extent of cell proliferation. [GOC:go_curators]	0.000604059
114	Os01g0754800,Os07g0564600,Os07g0645300,Os09g0456700,Os09g0542700,	GO:0006898	receptor-mediated endocytosis	An endocytosis process in which cell surface receptors ensure specificity of transport. A specific receptor on the cell surface binds tightly to the extracellular macromolecule (the ligand) that it recognizes; the plasma-membrane region containing the receptor-ligand complex then undergoes endocytosis, forming a transport vesicle containing the receptor-ligand complex and excluding most other plasma-membrane proteins. Receptor-mediated endocytosis generally occurs via clathrin-coated pits and vesicles. [GOC:mah, ISBN:0716731363]	0.000604059
14	Os01g0171800,Os01g0185900,Os02g0173900,Os03g0733600,Os04g0473150,Os04g0680400,Os05g0457600,Os07g0154800,Os07g0613200,Os07g0628400,Os08g0397800,Os08g0531000,Os09g0413100,Os10g0457400,Os12g0290200,	GO:0004038	allantoinase activity	Catalysis of the reaction: allantoin + H2O = allantoate. [EC:3.5.2.5]	0.000604157
31	Os01g0688300,Os03g0690500,Os03g0765800,Os04g0444300,Os05g0187500,Os06g0194400,Os06g0662000,Os07g0162900,Os07g0642600,Os07g0647200,Os08g0356800,Os09g0280500,Os09g0441000,Os11g0275000,Os12g0563400,	GO:0071368	cellular response to cytokinin stimulus	Any process that results in a change in state or activity of a cell (in terms of movement, secretion, enzyme production, gene expression, etc.) as a result of a cytokinin stimulus. [GOC:mah]	0.000604157
85	Os03g0169000,Os04g0370300,Os07g0646000,Os11g0241700,	GO:0034399	nuclear periphery	The portion of the nuclear lumen proximal to the inner nuclear membrane. [GOC:krc, GOC:mah]	0.000644317
90	Os01g0844400,Os01g0937900,Os04g0685600,Os08g0546100,	GO:0070062	extracellular vesicular exosome	A membrane-bounded vesicle that is released into the extracellular region by fusion of the limiting endosomal membrane of a multivesicular body with the plasma membrane. [GOC:BHF, GOC:mah, PMID:15908444, PMID:17641064]	0.000644317
122	Os02g0780600,Os10g0556500,Os11g0610900,Os12g0285100,	GO:0004482	mRNA (guanine-N7-)-methyltransferase activity	Catalysis of the reaction: S-adenosyl-L-methionine + G(5')pppR-RNA = S-adenosyl-L-homocysteine + m7G(5')pppR-RNA. m7G(5')pppR-RNA is mRNA containing an N7-methylguanine cap; R may be guanosine or adenosine. [EC:2.1.1.56]	0.000644317
28	Os01g0101600,Os01g0665750,Os02g0155700,Os02g0258800,Os03g0277100,Os09g0539100,Os11g0123066,Os12g0534000,	GO:0003856	3-dehydroquinate synthase activity	Catalysis of the reaction: 7-phospho-2-dehydro-3-deoxy-D-arabino-heptonate = 3-dehydroquinate + phosphate. [EC:4.2.3.4, RHEA:21971]	0.000644343
50	Os01g0198500,Os01g0279000,Os01g0605300,Os01g0740350,Os01g0846700,Os01g0947000,Os02g0728700,Os02g0798200,Os02g0823100,Os03g0192400,Os03g0306800,Os03g0409100,Os03g0651000,Os03g0669200,Os03g0780900,Os03g0823900,Os04g0611600,Os04g0686200,Os05g0303000,Os05g0355500,Os05g0357100,Os05g0407100,Os05g0489600,Os05g0532500,Os06g0103800,Os06g0284500,Os07g0189800,Os07g0591700,Os07g0623000,Os07g0630800,Os07g0673100,Os08g0417000,Os08g0417100,Os08g0472600,Os09g0395400,Os09g0478400,Os09g0518332,Os10g0114400,Os10g0140200,Os10g0476000,Os10g0488100,Os11g0116550,Os11g0249300,Os11g0487700,Os11g0594200,Os12g0161500,Os12g0632900,XLOC_001099,XLOC_006498,XLOC_008468,XLOC_013623,XLOC_059443,XLOC_059896,	GO:0002237	response to molecule of bacterial origin	Any process that results in a change in state or activity of an organism (in terms of movement, secretion, enzyme production, gene expression, etc.) as a result of a stimulus by molecules of bacterial origin such as peptides derived from bacterial flagellin. [GOC:rl, GOC:sm]	0.00065208
65	Os01g0292900,Os01g0578700,Os02g0264700,Os02g0536400,Os02g0556800,Os02g0644700,Os02g0719600,Os03g0252100,Os03g0789800,Os04g0451200,Os04g0512300,Os05g0564000,Os06g0214300,Os06g0284200,Os06g0291500,Os08g0413850,Os09g0409000,XLOC_030020,XLOC_030239,XLOC_031403,XLOC_033881,XLOC_056226,XLOC_064796,	GO:0080150	S-adenosyl-L-methionine:benzoic acid carboxyl methyl transferase activity	Catalysis of the reaction: benzoate + S-adenosyl-L-methionine = methylbenzoate + S-adenosyl-L-homocysteine. [MetaCyc:RXN-6722, PMID:10852939]	0.000684711
204	Os01g0300400,XLOC_068568,XLOC_076924,	GO:0000723	telomere maintenance	Any process that contributes to the maintenance of proper telomeric length and structure by affecting and monitoring the activity of telomeric proteins and the length of telomeric DNA. These processes includes those that shorten and lengthen the telomeric DNA sequences. [GOC:elh, PMID:11092831]	0.000684711

156	Os03g0283100,Os06g0687500,Os09g0564800,	GO:0010731	protein glutathionylation	The protein modification process in which a glutathione molecule is added to a protein amino acid through a disulfide linkage. [GOC:BHF, GOC:dph, GOC:rl, GOC:tb]	0.000724842
102	Os01g0311500,Os03g0818200,Os07g0546700,Os08g0169700,	GO:0016621	cinnamoyl-CoA reductase activity	Catalysis of the reaction: cinnamaldehyde + CoA + NADP+ = cinnamoyl-CoA + NADPH + H+. [EC:1.2.1.44]	0.000805347
103	Os01g0326000,Os04g0119400,Os12g0230600,Os12g0433500,	GO:0009558	embryo sac cellularization	The process in which the eight-nucleate single celled female gametophyte develops into the seven-celled female gametophyte. This mature structure contains two synergid cells and an egg cell at the micropylar end, and three antipodal cells at the other end. A binucleate endosperm mother cell is formed at the center. An example of this process is found in Arabidopsis thaliana. [GOC:jid, GOC:mtg_plant, GOC:mtg_sensu, ISBN:047186840X]	0.000805347
120	Os03g0104000,Os07g0543000,Os11g0592000,XLOC_041251,	GO:0080027	response to herbivore	Any process that results in a change in state or activity of a cell or an organism (in terms of movement, secretion, enzyme production, gene expression, etc.) as a result of a stimulus from a herbivore. [PMID:18987211]	0.000845615
38	Os01g0872700,Os02g0510000,Os03g0395600,Os04g0125800,Os04g0471400,Os07g0227800,Os10g0534900,	GO:0004534	5'-3' exoribonuclease activity	Catalysis of the sequential cleavage of mononucleotides from a free 5' terminus of an RNA molecule. [GOC:mah, ISBN:0198547684]	0.000845615
44	Os01g0113350,Os01g0558200,Os02g0678200,Os04g0395100,Os06g0104900,Os10g0485500,Os12g0293000,	GO:0004459	L-lactate dehydrogenase activity	Catalysis of the reaction: (S)-lactate + NAD+ = pyruvate + NADH + H+. [EC:1.1.1.27, RHEA:23447]	0.000845615
84	Os02g0198600,Os09g0458900,Os10g0182000,Os10g0567900,	GO:0004813	alanine-tRNA ligase activity	Catalysis of the reaction: ATP + L-alanine + tRNA(Ala) = AMP + diphosphate + L-alanyl-tRNA(Ala). [EC:6.1.1.7]	0.000966359
107	Os03g0262500,Os07g0688500,Os10g0357850,XLOC_064848,	GO:0031123	RNA 3'-end processing	Any process involved in forming the mature 3' end of an RNA molecule. [GOC:mah]	0.000966378
199	Os02g0290900,Os03g0223200,Os07g0605350,	GO:0018456	aryl-alcohol dehydrogenase (NAD+) activity	Catalysis of the reaction: an aromatic alcohol + NAD+ = an aromatic aldehyde + NADH + H+. [EC:1.1.1.90]	0.000966378
214	Os01g0135000,Os03g0351500,Os03g0728800,	GO:0019430	removal of superoxide radicals	Any process involved in removing superoxide radicals (O2-) from a cell or organism, e.g. by conversion to dioxygen (O2) and hydrogen peroxide (H2O2). [CHEBI:18421, GOC:jl]	0.000966378
40	Os01g0151400,Os02g0529600,Os02g0567100,Os05g0319700,Os05g0597200,Os06g0306300,Os06g0331900,Os08g0503000,	GO:0003840	gamma-glutamyltransferase activity	Catalysis of the reaction: (5-L-glutamyl)-peptide + an amino acid = peptide + 5-L-glutamyl-amino acid. [EC:2.3.2.2]	0.000966378
133	Os01g0773800,Os02g0137800,Os02g0709300,Os04g0105200,Os05g0424800,Os06g0222400,Os06g0610800,Os07g0111700,Os10g0543800,Os11g0178800,Os12g0182200,Os12g0615400,	GO:0051741	2-methyl-6-phytyl-1,4-benzoquinone methyltransferase activity	Catalysis of the reaction: 2-methyl-6-phytyl-1,4-benzoquinone + S-adenosyl-methionine = 2,3-dimethyl-6-phytyl-1,4-benzoquinone + S-adenosyl-homocysteine. [MetaCyc:RXN-2542]	0.000966436
63	Os04g0221600,Os04g0298600,Os04g0517350,Os06g0361500,Os11g0615000,	GO:0008195	phosphatidate phosphatase activity	Catalysis of the reaction: a 1,2-diacylglycerol 3-phosphate + H2O = a 1,2-diacyl-sn-glycerol + phosphate. [EC:3.1.3.4, GOC:pr]	0.001006603
7	Os01g0200600,Os01g0234700,Os01g0616366,Os01g0715800,Os01g0785700,Os01g0811400,Os01g0895200,Os01g0907300,Os01g0921600,Os01g0935700,Os02g0181900,Os02g0555000,Os02g0700500,Os02g0775600,Os02g0816400,Os02g0818700,Os03g0263000,Os03g0271500,Os03g0621400,Os03g0792500,Os04g0395700,Os04g0419550,Os04g0496300,Os04g0597300,Os05g0165450,Os05g0272900,Os05g0414400,Os05g0419100,Os06g0234200,Os06g0574100,Os06g0694200,Os06g0713000,Os07g0229900,Os07g0490300,Os07g0622000,Os08g0263300,Os08g0281500,Os08g0326600,Os09g0132600,Os09g0314400,Os09g0364500,Os10g0190100,Os10g0411700,Os10g0421500,Os10g0512700,Os10g0562700,Os11g0297000,Os11g0433200,Os11g0544300,Os11g0565400,Os12g0115300,XLOC_001874,XLOC_010893,XLOC_015818,XLOC_067697,XLOC_073131,XLOC_074955,	GO:0005774	vacuolar membrane	The lipid bilayer surrounding the vacuole and separating its contents from the cytoplasm of the cell. [GOC:ai]	0.001016499

137	Os01g0928400,Os03g0284800,Os03g0741600,	GO:0009957	epidermal cell fate specification	The process in which a cell becomes capable of differentiating autonomously into an epidermal cell in an environment that is neutral with respect to the developmental pathway; upon specification, the cell fate can be reversed. [GOC:mtg_sensu, GOC:sm]	0.001087131
166	Os01g0702500,Os04g0422300,Os07g0467600,	GO:0009415	response to water	Any process that results in a change in state or activity of a cell or an organism (in terms of movement, secretion, enzyme production, gene expression, etc.) as a result of a stimulus reflecting the presence, absence, or concentration of water. [GOC:jl]	0.001087131
71	Os04g0672200,Os06g0315900,Os08g0465800,Os10g0316400,XLOC_058130,	GO:0004351	glutamate decarboxylase activity	Catalysis of the reaction: L-glutamate = 4-aminobutanoate + CO2. [EC:4.1.1.15]	0.00112735
23	Os01g0301300,Os01g0844500,Os01g0913000,Os02g0106800,Os02g0276400,Os02g0706400,Os03g0243950,Os03g0782400,Os05g0214232,Os08g0412700,Os08g0433600,Os08g0497900,Os09g0544000,Os12g0288400,XLOC_045400,	GO:0080148	negative regulation of response to water deprivation	Any process that stops, prevents, or reduces the frequency, rate or extent of a response to water deprivation. Response to water deprivation is a change in state or activity of a cell or an organism (in terms of movement, secretion, enzyme production, gene expression, etc.) as a result of a water deprivation stimulus, prolonged deprivation of water. [PMID:18835996]	0.001127464
111	Os01g0877600,Os02g0433600,Os04g0172400,Os05g0212400,Os05g0467300,Os05g0479900,XLOC_066757,	GO:0008430	selenium binding	Interacting selectively and non-covalently with selenium (Se). [GOC:ai]	0.001207827
19	Os01g0763600,Os02g0778500,Os03g0292100,Os04g0566100,Os04g0640600,Os05g0542150,Os06g0608700,Os08g0506350,Os11g0592700,Os12g0116200,	GO:0005504	fatty acid binding	Interacting selectively and non-covalently with fatty acids, aliphatic monocarboxylic acids liberated from naturally occurring fats and oils by hydrolysis. [ISBN:0198506732]	0.001207875
109	Os03g0381200,Os03g0669000,Os03g0745000,Os03g0752500,Os04g0272200,Os06g0712900,Os07g0116600,Os09g0447300,Os10g0553600,Os12g0231100,	GO:0008290	F-actin capping protein complex	A heterodimer consisting of alpha and beta subunits that binds to and caps the barbed ends of actin filaments, thereby regulating the polymerization of actin monomers but not severing actin filaments. [GOC:go_curators, ISBN:0198599560]	0.001207875
182	Os03g0805766,Os10g0477900,Os10g0532300,	GO:0003854	3-beta-hydroxy-delta5-steroid dehydrogenase activity	Catalysis of the reaction: a 3-beta-hydroxy-delta(5)-steroid + NAD+ = a 3-oxo-delta(5)-steroid + NADH + H(+). [EC:1.1.1.145]	0.001328609
87	Os07g0154400,Os08g0439100,XLOC_024108,XLOC_040357,	GO:0035091	phosphatidylinositol binding	Interacting selectively and non-covalently with any inositol-containing glycerophospholipid, i.e. phosphatidylinositol (PtdIns) and its phosphorylated derivatives. [GOC:bf, ISBN:0198506732, PMID:11395417]	0.00136898
112	Os01g0821700,Os01g0922600,Os02g0174100,Os02g0598350,Os02g0815400,Os06g0336150,Os08g0509600,Os09g0513100,Os12g0189300,XLOC_017209,XLOC_054029,	GO:0004607	phosphatidylcholine-sterol O-acyltransferase activity	Catalysis of the reaction: phosphatidylcholine + a sterol = a sterol ester + 1-acylglycerophosphocholine. [EC:2.3.1.43]	0.001449275
79	Os01g0321700,Os03g0737800,Os05g0366300,Os06g0699900,	GO:0042176	regulation of protein catabolic process	Any process that modulates the frequency, rate or extent of the chemical reactions and pathways resulting in the breakdown of a protein by the destruction of the native, active configuration, with or without the hydrolysis of peptide bonds. [GOC:go_curators, GOC:jl]	0.001449275
96	Os03g0744800,Os06g0339800,Os09g0549300,Os10g0377150,	GO:0046520	sphingoid biosynthetic process	The chemical reactions and pathways resulting in the formation of sphingoids, any of a class of compounds comprising sphinganine and its homologues and stereoisomers, and derivatives of these compounds. [ISBN:0198506732]	0.001449275
105	Os01g0763200,Os02g0526700,Os08g0160300,Os08g0249100,	GO:0002238	response to molecule of fungal origin	Any process that results in a change in state or activity of an organism (in terms of movement, secretion, enzyme production, gene expression, etc.) as a result of a stimulus by molecules of fungal origin such as chito-octamer oligosaccharide. [GOC:rl, GOC:sm]	0.001449275
118	Os02g0279600,Os04g0164300,Os08g0545000,Os09g0437500,	GO:0046786	viral replication complex formation and maintenance	The process of organizing and assembling viral replication proteins in preparation for viral replication. [ISBN:0781718325]	0.001449275
202	Os02g0189800,Os04g0491100,Os07g0568100,	GO:0010541	acropetal auxin transport	The unidirectional movement of auxin from the base towards the apex of an organ, including the shoot, leaf, primary root, or lateral root. [PMID:10677441]	0.001449333

15	Os01g0780900,Os01g0958000,Os02g0679500,Os02g0705600,Os04g0423400,Os05g0135500,Os07g0131250,Os09g0272000,Os09g0327300,Os09g0550000,Os10g0490800,Os10g0518800,	GO:0050792	regulation of viral process	Any process that modulates the rate or extent of the viral life cycle, the set of processes by which a virus reproduces and spreads among hosts. [GOC:go_curators, GOC:tb]	0.001449333
206	Os01g0604700,Os12g0618600,XLOC_071807,	GO:0016602	CCAAT-binding factor complex	A heteromeric transcription factor complex that binds to the CCAAT-box upstream of promoters; in Saccharomyces it activates the transcription of genes in response to growth in a nonfermentable carbon source; consists of four known subunits: HAP2, HAP3, HAP4 and HAP5. [PMID:7828851]	0.001449479
6	Os01g0210800,Os01g0214200,Os01g0749300,Os01g0777700,Os01g0868200,Os01g0915000,Os02g0146500,Os02g0148100,Os02g0539500,Os02g0823700,Os03g0221200,Os03g0267700,Os03g0690600,Os03g0750700,Os03g0815800,Os04g0438700,Os04g0687800,Os05g0111300,Os05g0531000,Os05g0545400,Os05g0579300,Os06g0164000,Os06g0672400,Os07g0541200,Os08g0478566,Os09g0134500,Os09g0481600,Os09g0516300,Os10g0391100,Os10g0495300,Os10g0521500,Os10g0524400,Os10g0572300,Os10g0577200,Os11g0147500,Os12g0175900,Os12g0586000,Os12g0615800,XLOC_049097,	GO:0000124	SAGA complex	A SAGA-type histone acetyltransferase complex that contains Spt8 (in budding yeast) or a homolog thereof; additional polypeptides include Spt group, consisting of Spt7, Spt3, and Spt20/Ada5, which interact with the TATA-binding protein (TBP); the Ada group, consisting of Ada1, Ada2, Ada3, Ada4/Gcn5, and Ada5/Spt20, which is functionally linked to the nucleosomal HAT activity; Tra1, an ATM/PI-3 kinase-related protein that targets DNA-bound activators for recruitment to promoters; the TBP-associated factor (TAF) proteins, consisting of Taf5, Taf6, Taf9, Taf10, and Taf12, which mediate nucleosomal HAT activity and are thought to help recruit the basal transcription machinery. [PMID:10637607, PMID:17337012]	0.00153053
42	Os01g0196800,Os01g0237000,Os01g0562600,Os01g0680950,Os01g0905300,Os01g0961300,Os03g0233300,Os03g0296800,Os03g0330200,Os03g0698800,Os03g0823000,Os03g0828300,Os04g0505700,Os05g0338933,Os05g0366600,Os05g0549700,Os06g0695500,Os07g0273900,Os07g0641800,Os08g0439000,Os08g0483200,Os08g0556900,Os09g0246300,Os09g0329200,Os09g0346500,Os09g0413000,Os09g0511700,Os09g0547500,Os10g0183500,Os10g0212100,Os10g0391500,Os10g0503300,Os11g0151700,Os11g0167200,Os11g0217300,Os12g0162100,Os12g0228400,Os12g0618800,XLOC_003338,XLOC_014220,XLOC_018392,XLOC_019586,XLOC_025312,XLOC_027543,XLOC_031093,XLOC_037399,XLOC_041683,XLOC_056178,XLOC_058089,	GO:0047940	glucuronokinase activity	Catalysis of the reaction: D-glucuronate + ATP = 1-phospho-alpha-D-glucuronate + ADP + 2 H(+). [EC:2.7.1.43, RHEA:17008]	0.00153053
34	Os05g0272800,Os05g0401500,Os05g0527900,Os07g0620300,Os08g0113000,Os09g0499450,Os10g0562200,Os11g0274700,	GO:0042398	cellular modified amino acid biosynthetic process	The chemical reactions and pathways resulting in the formation of compounds derived from amino acids, organic acids containing one or more amino substituents. [CHEBI:25359, GOC:ai]	0.001610176
46	Os02g0179800,Os02g0801300,Os03g0168300,Os04g0662600,Os07g0296100,Os07g0474700,Os09g0115600,Os12g0206700,XLOC_066660,	GO:0004629	phospholipase C activity	Catalysis of the reaction: a phospholipid + H2O = 1,2-diacylglycerol + a phosphatidate. [EC:3.1.4.3, EC:3.1.4.4, GOC:mah]	0.001610176
100	Os02g0810200,Os03g0288500,Os05g0116000,Os12g0601300,	GO:0010102	lateral root morphogenesis	The process in which the anatomical structures of a lateral root are generated and organized. A lateral root is one formed from pericycle cells located on the xylem radius of the root, as opposed to the initiation of the main root from the embryo proper. [GOC:tair_curators]	0.001610208
101	Os01g0859500,Os08g0564500,Os09g0314300,Os09g0369400,	GO:0004805	trehalose-phosphatase activity	Catalysis of the reaction: trehalose 6-phosphate + H2O = trehalose + phosphate. [EC:3.1.3.12]	0.001610208
86	Os04g0610100,Os07g0164700,Os08g0112000,Os08g0544600,	GO:0004386	helicase activity	Catalysis of the reaction: NTP + H2O = NDP + phosphate, to drive the unwinding of a DNA or RNA helix. [GOC:mah, ISBN:0198506732]	0.001803375
144	Os04g0436000,Os06g0282000,Os06g0652250,Os10g0577500,Os11g0488400,	GO:0080044	quercetin 7-O-glucosyltransferase activity	Catalysis of the transfer of a glucosyl group from UDP-glucose to the 7-hydroxy group of a quercetin molecule. [PMID:15352060]	0.002012396
98	Os02g0833300,Os04g0308000,Os07g0211900,Os12g0233800,	GO:0043130	ubiquitin binding	Interacting selectively and non-covalently with ubiquitin, a protein that when covalently bound to other cellular proteins marks them for proteolytic degradation. [GOC:ecd]	0.002092892
125	Os01g0720300,Os02g0112000,Os06g0183700,Os10g0159800,	GO:0016651	oxidoreductase activity, acting on NAD(P)H	Catalysis of an oxidation-reduction (redox) reaction in which NADH or NADPH acts as a hydrogen or electron donor and reduces a hydrogen or electron acceptor. [GOC:ai]	0.002092892
58	Os01g0704100,Os02g0651900,Os06g0612951,Os08g0425300,Os12g0106801,	GO:0015112	nitrate transmembrane transporter activity	Catalysis of the transfer of nitrate ions (NO3-) from one side of a membrane to the other. [GOC:ai]	0.002213457

67	Os01g0267200,Os03g0641250,Os03g0685300,Os04g0423800,Os08g0515900,Os09g0564200,Os10g0495900,Os11g0303800,	GO:0080110	sporopollenin biosynthetic process	The chemical reactions and pathways resulting in the formation of sporopollenin, a primary constituent of the pollen exine layer. [PMID:19218397]	0.002253611
5	Os01g0148400,Os01g0512200,Os01g0689900,Os01g0737100,Os01g0823200,Os01g0867600,Os01g0955700,Os02g0144300,Os03g0125900,Os03g0179400,Os03g0264150,Os03g0264400,Os03g0362200,Os03g0587100,Os03g0684500,Os03g0756200,Os03g0789466,Os03g0816500,Os03g0835800,Os03g0852800,Os04g0501100,Os04g0529400,Os04g0558700,Os04g0594100,Os05g0110000,Os05g0318600,Os05g0391500,Os05g0474900,Os05g0511400,Os05g0537400,Os06g0110000,Os06g0140200,Os06g0218500,Os06g0222800,Os06g0552400,Os06g0560300,Os06g0649000,Os06g0675600,Os06g0698200,Os07g0111600,Os07g0643800,Os08g0387400,Os08g0433500,Os08g0564800,Os09g0109800,Os09g0360900,Os09g0397800,Os09g0498200,Os09g0509100,Os10g0130500,Os10g0135500,Os10g0324600,Os10g0495416,Os10g0515200,Os11g0201175,Os11g0452400,Os11g0535600,Os12g0609100,Os12g0626500,XLOC_009492,XLOC_020260,XLOC_025834,XLOC_026516,XLOC_030698,XLOC_032273,XLOC_032724,XLOC_040629,XLOC_040981,XLOC_044441,XLOC_044634,XLOC_051599,XLOC_055176,XLOC_060222,XLOC_067770,XLOC_077187,	GO:0006848	pyruvate transport	The directed movement of pyruvate into, out of or within a cell, or between cells, by means of some agent such as a transporter or pore. [GOC:krc]	0.002376349
13	Os01g0706000,Os01g0900900,Os02g0175100,Os03g0113000,Os03g0227900,Os03g0279600,Os03g0645100,Os03g0686900,Os03g0852500,Os04g0297800,Os04g0432000,Os05g0557800,Os06g0232000,Os06g0319700,Os06g0597400,Os06g0621800,Os06g0642550,Os07g0520400,Os08g0112800,Os08g0159700,Os09g0370000,Os10g0407100,Os10g0540800,Os10g0554200,Os12g0263100,	GO:0051567	histone H3-K9 methylation	The modification of histone H3 by addition of a methyl group to lysine at position 9 of the histone. [GOC:ai]	0.002382839
115	Os02g0190000,Os04g0534000,Os05g0313500,Os05g0353500,Os10g0474800,	GO:0010541	acropetal auxin transport	The unidirectional movement of auxin from the base towards the apex of an organ, including the shoot, leaf, primary root, or lateral root. [PMID:10677441]	0.002414486
78	Os01g0639900,Os01g0894500,Os01g0921000,Os02g0313400,Os03g0107300,Os03g0221500,Os03g0255200,Os04g0128001,Os04g0644100,Os04g0677100,Os05g0371200,Os05g0541100,Os05g0586500,Os06g0287500,Os06g0551500,Os08g0162300,Os08g0234200,Os08g0288050,Os10g0580800,Os12g0601800,XLOC_034785,XLOC_056467,	GO:0010037	response to carbon dioxide	Any process that results in a change in state or activity of a cell or an organism (in terms of movement, secretion, enzyme production, gene expression, etc.) as a result of a carbon dioxide (CO2) stimulus. [GOC:sm]	0.002414777
1	Os01g0109700,Os01g0180300,Os01g0310100,Os01g0532300,Os01g0632700,Os01g0639600,Os01g0655300,Os01g0672300,Os01g0688900,Os01g0793800,Os01g0800250,Os01g0839100,Os01g0879300,Os01g0931100,Os01g0956500,Os01g0971800,Os02g0133300,Os02g0184300,Os02g0282900,Os02g0317600,Os02g0526500,Os02g0631601,Os02g0648300,Os02g0701600,Os02g0702000,Os02g0714300,Os02g0822900,Os02g0823800,Os03g0111700,Os03g0115400,Os03g0126000,Os03g0130400,Os03g0162000,Os03g0205400,Os03g0245700,Os03g0324200,Os03g0324300,Os03g0326500,Os03g0336900,Os03g0345100,Os03g0395900,Os03g0399600,Os03g0565300,Os03g0712600,Os03g0752700,Os03g0764900,Os03g0793800,Os03g0808100,Os03g0812300,Os04g0388500,Os04g0433300,Os04g0442700,Os04g0450000,Os04g0471300,Os04g0508500,Os04g0542000,Os04g0565500,Os04g0568850,Os04g0661700,Os04g0671800,Os04g0687300,Os05g0156800,Os05g0161800,Os05g0238200,Os05g0392700,Os05g0474600,Os05g0494900,Os05g0552100,Os05g0557400,Os05g0576600,Os06g0111700,Os06g0155400,Os06g0166200,Os06g0334400,Os06g0335101,Os06g0594700,Os06g0603300,Os06g0622900,Os06g0645901,Os06g0697000,Os06g0702000,Os07g0230600,Os07g0490500,Os07g0497100,Os07g0599300,Os07g0607800,Os08g0148267,Os08g0150600,Os08g0154225,Os08g0159900,Os08g0337300,Os08g0404500,Os08g0459100,Os08g0508800,Os08g0517800,Os08g0519800,Os08g0531200,Os08g0535900,Os08g0550400,Os09g0290900,Os09g0371000,Os09g0391800,Os09g0474800,Os09g0539200,Os09g0542900,Os10g0457600,Os10g0465700,Os10g0537600,Os11g0207600,Os11g0217500,Os11g0456100,Os11g0490900,Os11g0598300,Os12g0105600,	GO:0004519	endonuclease activity	Catalysis of the hydrolysis of ester linkages within nucleic acids by creating internal breaks. [GOC:mah, ISBN:0198547684]	0.002542012
91	Os01g0565900,Os03g0341000,Os06g0218200,Os10g0498600,	GO:0009628	response to abiotic stimulus	Any process that results in a change in state or activity of a cell or an organism (in terms of movement, secretion, enzyme production, gene expression, etc.) as a result of an abiotic (non-living) stimulus. [GOC:hb]	0.0025754
223	Os05g0589700,Os09g0383300,Os12g0560500,XLOC_036022,	GO:0009982	pseudouridine synthase activity	Catalysis of the reaction: RNA uridine = RNA pseudouridine. Conversion of uridine in an RNA molecule to pseudouridine by rotation of the C1'-N-1 glycosidic bond of uridine in RNA to a C1'-C5. [EC:5.4.99.12, GOC:mah]	0.002776658
72	Os02g0272350,Os04g0118800,Os07g0277600,Os12g0246700,Os12g0556300,	GO:0043531	ADP binding	Interacting selectively and non-covalently with ADP, adenosine 5'-diphosphate. [GOC:jl]	0.002811981
18	Os01g0183300,Os01g0296000,Os04g0417000,Os04g0481700,Os05g0539400,Os06g0129650,Os07g0520300,Os11g0134600,Os11g0657850,Os12g0582700,	GO:0015140	malate transmembrane transporter activity	Catalysis of the transfer of malate from one side of the membrane to the other. Malate is a chiral hydroxydicarboxylic acid, hydroxybutanedioic acid. The (+) enantiomer is an important intermediate in metabolism as a component of both the TCA cycle and the glyoxylate cycle. [GOC:ai]	0.002816333
25	Os01g0736551,Os01g0847700,Os04g0634000,Os05g0149000,Os05g0468750,Os06g0297800,Os08g0538200,Os10g0380100,Os11g0175800,	GO:0042787	protein ubiquitination involved in ubiquitin-dependent protein catabolic process	The process in which a ubiquitin group, or multiple groups, are covalently attached to the target protein, thereby initiating the degradation of that protein. [GOC:go_curators]	0.002896683

4	Os01g0111900,Os01g0230200,Os01g0368000,Os01g0580800,Os01g0819700,Os01g0835700,Os01g0889000,Os01g0951200,Os01g0969100,Os02g0122700,Os02g0187800,Os02g0308800,Os02g0554800,Os02g0554900,Os02g0717800,Os02g0782800,Os02g0807750,Os03g0148000,Os03g0169100,Os03g0411800,Os03g0579200,Os03g0684700,Os03g0738900,Os03g0785800,Os03g0806700,Os03g0811600,Os03g0826200,Os03g0831400,Os04g0393500,Os04g0398000,Os04g0416500,Os04g0432100,Os04g0524400,Os04g0589700,Os04g0629200,Os04g0640500,Os04g0641400,Os04g0645200,Os04g0657500,Os05g0215300,Os05g0324700,Os05g0342900,Os05g0444200,Os05g0474800,Os05g0541800,Os05g0564700,Os06g0142800,Os06g0167600,Os06g0195800,Os06g0208200,Os06g0239200,Os06g0267500,Os06g0523400,Os06g0608600,Os07g0171100,Os07g0196300,Os07g0530600,Os07g0556300,Os08g0151900,Os08g0200750,Os08g0376600,Os08g0504400,Os08g0512900,Os08g0529000,Os08g0547600,Os09g0487900,Os09g0518500,Os09g0538400,Os09g0569800,Os10g0395000,Os10g0554800,Os11g0151200,Os11g0305400,Os11g0582300,Os12g0144700,Os12g0152600,Os12g0219700,Os12g0443700,Os12g0570400,Os12g0609600,Os12g0624300,	GO:0005456	CMP-N-acetylneuraminate transmembrane transporter activity	Catalysis of the transfer of a CMP-N-acetylneuraminate from one side of the membrane to the other. [GOC:ai, GOC:mtg_transport, ISBN:0815340729]	0.003262446
56	Os01g0604100,Os03g0596800,Os03g0789600,Os03g0793100,Os05g0203912,Os11g0180100,	GO:0015018	galactosylgalactosylxylosylprotein 3-beta-glucuronosyltransferase activity	Catalysis of the reaction: UDP-glucuronate + 3-beta-D-galactosyl-4-beta-D-galactosyl-O-beta-D-xylosylprotein = UDP + 3-beta-D-glucuronosyl-3-beta-D-galactosyl-4-beta-D-galactosyl-O-beta-D-xylosylprotein. [EC:2.4.1.135]	0.003378851
145	Os01g0631100,Os02g0122800,Os04g0430000,	GO:0016607	nuclear speck	A discrete extra-nucleolar subnuclear domain, 20-50 in number, in which splicing factors are seen to be localized by immunofluorescence microscopy. [http://www.cellnucleus.com/]	0.003379599
41	Os01g0231000,Os01g0383900,Os03g0137600,Os03g0751600,Os06g0693700,Os07g0481300,Os10g0147900,Os12g0446500,	GO:0090404	pollen tube tip	The region at growing end of the pollen tube cell, where polarized growth occurs. [GOC:tb, PO:0025195, PO:0025281]	0.003539392
220	Os01g0952800,Os02g0714600,Os03g0194600,Os03g0259300,Os04g0340550,Os04g0573900,Os06g0167200,Os06g0189600,Os09g0542200,Os11g0237300,Os12g0244200,Os12g0637000,XLOC_001252,XLOC_003953,	GO:0030433	ER-associated ubiquitin-dependent protein catabolic process	The chemical reactions and pathways resulting in the breakdown of proteins transported from the endoplasmic reticulum and targeted to cytoplasmic proteasomes for degradation. This process acts on misfolded proteins as well as in the regulated degradation of correctly folded proteins. [GOC:mah, GOC:rb, PMID:14607247, PMID:19520858]	0.003860611
132	Os01g0195400,Os01g0977600,Os02g0564300,Os03g0619600,Os04g0183500,Os07g0417400,Os11g0157600,	GO:0005375	copper ion transmembrane transporter activity	Catalysis of the transfer of copper (Cu) ions from one side of a membrane to the other. [GOC:ai]	0.003940961
81	Os01g0221300,Os01g0766300,Os02g0120000,Os06g0509100,Os09g0363100,	GO:0004707	MAP kinase activity	Catalysis of the reaction: protein + ATP = protein phosphate + ADP. This reaction is the phosphorylation of proteins. Mitogen-activated protein kinase; a family of protein kinases that perform a crucial step in relaying signals from the plasma membrane to the nucleus. They are activated by a wide range of proliferation- or differentiation-inducing signals; activation is strong with agonists such as polypeptide growth factors and tumor-promoting phorbol esters, but weak (in most cell backgrounds) by stress stimuli. [GOC:ma, ISBN:0198547684]	0.004222288
95	Os04g0166000,Os05g0477500,Os12g0552700,Os12g0615500,	GO:0009407	toxin catabolic process	The chemical reactions and pathways resulting in the breakdown of toxin, a poisonous compound (typically a protein) that is produced by cells or organisms and that can cause disease when introduced into the body or tissues of an organism. [GOC:go_curators]	0.004343099
12	Os01g0768200,Os02g0570300,Os02g0612900,Os03g0795200,Os04g0525700,Os04g0560100,Os05g0410200,Os05g0452500,Os05g0569300,Os07g0538000,Os10g0438600,Os10g0563800,Os12g0566600,	GO:0031425	chloroplast RNA processing	The conversion of a primary RNA molecule transcribed from a chloroplast genome into one or more mature RNA molecules. [GOC:mah]	0.00470332
142	Os03g0729100,Os04g0391500,Os09g0556200,	GO:0016597	amino acid binding	Interacting selectively and non-covalently with an amino acid, organic acids containing one or more amino substituents. [GOC:ai]	0.004946107
147	Os01g0834600,Os02g0714500,Os05g0177100,Os07g0109400,Os11g0585700,	GO:0005198	structural molecule activity	The action of a molecule that contributes to the structural integrity of a complex or assembly within or outside a cell. [GOC:mah]	0.004990556
126	Os03g0822300,Os12g0168400,Os12g0175700,XLOC_032633,	GO:0019243	methylglyoxal catabolic process to D-lactate	The chemical reactions and pathways resulting in the breakdown of methylglyoxal, CH3-CO-CHO, into D-lactate via the intermediate S-lactoyl-glutathione. Glutathione is used in the first step of the pathway and then regenerated in the second step. [GOC:ai]	0.005066539
135	Os03g0125300,Os03g0685750,Os06g0342500,	GO:0003676	nucleic acid binding	Interacting selectively and non-covalently with any nucleic acid. [GOC:jl]	0.005268685

123	Os02g0794500,Os03g0366100,Os05g0127500,Os07g0508900,	GO:0009742	brassinosteroid mediated signaling pathway	A series of molecular signals mediated by the detection of brassinosteroid. [GOC:sm]	0.006268817
183	Os05g0429400,Os06g0314000,Os11g0213600,	GO:0004180	carboxypeptidase activity	Catalysis of the hydrolysis of the terminal or penultimate peptide bond at the C-terminal end of a peptide or polypeptide. [ISBN:0198506732]	0.006510972
16	Os02g0550300,Os02g0729100,Os03g0817800,Os03g0849500,Os04g0110500,Os04g0510500,Os05g0369500,Os06g0238000,Os06g0589600,Os06g0641932,Os06g0677600,Os06g0683000,Os06g0695700,Os08g0111300,Os08g0424100,Os08g0495500,Os08g0502400,Os09g0400400,Os10g0441900,Os10g0450000,Os10g0481300,	GO:0018456	aryl-alcohol dehydrogenase (NAD+) activity	Catalysis of the reaction: an aromatic alcohol + NAD+ = an aromatic aldehyde + NADH + H+. [EC:1.1.1.90]	0.006747505
222	Os01g0690900,Os01g0828100,Os03g0336700,Os04g0490600,Os04g0674500,Os06g0541600,Os08g0526350,	GO:0005351	sugar:hydrogen symporter activity	Catalysis of the transfer of a solute or solutes from one side of a membrane to the other according to the reaction: sugar(out) + H+(out) = sugar(in) + H+(in). [TC:2.A.1.1.-]	0.007028084
92	Os02g0110500,Os03g0134800,Os03g0719000,Os05g0391600,	GO:0000910	cytokinesis	The division of the cytoplasm and the plasma membrane of a cell and its separation into two daughter cells. [GOC:mtg_cell_cycle]	0.007390862
218	Os06g0646000,Os10g0503800,Os12g0242800,	GO:0010287	plastoglobule	A lipoprotein particle present in chloroplasts. They are rich in non-polar lipids (triglycerides, esters) as well as in prenylquinones, plastoquinone and tocopherols. Plastoglobules are often associated with thylakoid membranes, suggesting an exchange of lipids with thylakoids. [GOC:tair_curators, PMID:16461379]	0.007954006
216	Os04g0634600,Os05g0163400,Os05g0334400,	GO:0031072	heat shock protein binding	Interacting selectively and non-covalently with a heat shock protein, any protein synthesized or activated in response to heat shock. [GOC:mah, GOC:vw]	0.00879513
27	Os01g0630300,Os02g0162150,Os03g0328900,Os04g0604200,Os07g0537600,Os08g0115200,Os10g0538450,Os12g0594950,XLOC_008525,	GO:0006073	cellular glucan metabolic process	The chemical reactions and pathways involving glucans, polysaccharides consisting only of glucose residues. [ISBN:0198547684]	0.008987803
200	Os02g0274700,Os09g0570500,Os12g0114200,	GO:0008270	zinc ion binding	Interacting selectively and non-covalently with zinc (Zn) ions. [GOC:ai]	0.009921266
26	Os01g0136500,Os01g0949900,Os02g0557500,Os04g0617600,Os04g0626500,Os05g0420200,Os08g0288500,Os09g0532000,	GO:0015996	chlorophyll catabolic process	The chemical reactions and pathways resulting in the breakdown of chlorophyll, any compound of magnesium complexed in a porphyrin (tetrapyrrole) ring and which functions as a photosynthetic pigment, into less complex products. [GOC:jl]	0.009946576
29	Os01g0186000,Os03g0263350,Os04g0368800,Os04g0685400,Os07g0106100,Os08g0102700,Os11g0111800,XLOC_055417,	GO:0051607	defense response to virus	Reactions triggered in response to the presence of a virus that act to protect the cell or organism. [GOC:ai]	0.010386498
192	Os06g0309200,XLOC_040280,XLOC_071610,	GO:0008289	lipid binding	Interacting selectively and non-covalently with a lipid. [GOC:ai]	0.010874819
148	Os05g0241000,Os07g0690800,Os10g0561500,	GO:0000151	ubiquitin ligase complex	A protein complex that includes a ubiquitin-protein ligase and other proteins that may confer substrate specificity on the complex. [GOC:jh2, PMID:9529603]	0.011435568
74	Os01g0222700,Os04g0466100,Os04g0548100,Os07g0172600,Os07g0570600,	GO:0004222	metalloendopeptidase activity	Catalysis of the hydrolysis of internal, alpha-peptide bonds in a polypeptide chain by a mechanism in which water acts as a nucleophile, one or two metal ions hold the water molecule in place, and charged amino acid side chains are ligands for the metal ions. [GOC:mah, http://merops.sanger.ac.uk/about/glossary.htm#CATTYPE, http://merops.sanger.ac.uk/about/glossary.htm#ENDOPEPTIDASE]	0.012823426
139	Os03g0646900,Os03g0711800,Os11g0608000,	GO:0046777	protein autophosphorylation	The phosphorylation by a protein of one or more of its own amino acid residues, or residues on an identical protein. [ISBN:0198506732]	0.01299362

117	Os02g0608100,Os03g0184300,Os07g0627500,Os07g0687900,	GO:0016757	transferase activity, transferring glycosyl groups	Catalysis of the transfer of a glycosyl group from one compound (donor) to another (acceptor). [GOC:jl, ISBN:0198506732]	0.013375229
143	Os06g0697500,XLOC_054355,XLOC_071313,	GO:0017111	nucleoside-triphosphatase activity	Catalysis of the reaction: a nucleoside triphosphate + H2O = nucleoside diphosphate + phosphate. [EC:3.6.1.15]	0.013492831
130	Os02g0642200,Os03g0372700,Os11g0264600,Os11g0629400,	GO:0005874	microtubule	Any of the long, generally straight, hollow tubes of internal diameter 12-15 nm and external diameter 24 nm found in a wide variety of eukaryotic cells; each consists (usually) of 13 protofilaments of polymeric tubulin, staggered in such a manner that the tubulin monomers are arranged in a helical pattern on the microtubular surface, and with the alpha/beta axes of the tubulin subunits parallel to the long axis of the tubule; exist in equilibrium with pool of tubulin monomers and can be rapidly assembled or disassembled in response to physiological stimuli; concerned with force generation, e.g. in the [illegible]	0.015218594
140	Os11g0588400,XLOC_007198,XLOC_054077,	GO:0043531	ADP binding	Interacting selectively and non-covalently with ADP, adenosine 5'-diphosphate. [GOC:jl]	0.017077493
161	Os02g0562600,XLOC_044650,XLOC_045368,	GO:0006952	defense response	Reactions, triggered in response to the presence of a foreign body or the occurrence of an injury, which result in restriction of damage to the organism attacked or prevention/recovery from the infection caused by the attack. [GOC:go_curators]	0.017480264
82	Os02g0562400,Os02g0718600,Os03g0412300,Os11g0162000,	GO:0030001	metal ion transport	The directed movement of metal ions, any metal ion with an electric charge, into, out of or within a cell, or between cells, by means of some agent such as a transporter or pore. [GOC:ai]	0.017923489
173	Os01g0691500,XLOC_035144,XLOC_040426,	GO:0009941	chloroplast envelope	The double lipid bilayer enclosing the chloroplast and separating its contents from the rest of the cytoplasm; includes the intermembrane space. [GOC:tb]	0.023844047
158	Os06g0279900,Os07g0545800,XLOC_047988,	GO:0017111	nucleoside-triphosphatase activity	Catalysis of the reaction: a nucleoside triphosphate + H2O = nucleoside diphosphate + phosphate. [EC:3.6.1.15]	0.026804141
208	Os06g0273100,Os07g0619200,XLOC_045126,	GO:0005509	calcium ion binding	Interacting selectively and non-covalently with calcium ions (Ca2+). [GOC:ai]	0.030852835
180	Os05g0592500,Os09g0444800,XLOC_031802,	GO:0005488	binding	The selective, non-covalent, often stoichiometric, interaction of a molecule with one or more specific sites on another molecule. [GOC:ceb, GOC:mah, ISBN:0198506732]	0.033626467
169	Os01g0179800,Os02g0637200,Os10g0343200,	GO:0009793	embryo development ending in seed dormancy	The process whose specific outcome is the progression of the embryo over time, from zygote formation to the end of seed dormancy. An example of this process is found in Arabidopsis thaliana. [GOC:go_curators, GOC:mtg_sensu]	0.036050427
185	Os02g0231700,Os02g0820600,Os12g0551200,	GO:0005507	copper ion binding	Interacting selectively and non-covalently with copper (Cu) ions. [GOC:ai]	0.0394659
48	Os01g0668600,Os01g0797800,Os03g0194900,Os03g0294100,Os07g0417800,Os08g0105100,Os09g0517100,Os10g0213800,Os11g0131800,Os12g0102100,XLOC_030611,	GO:0048544	recognition of pollen	The process, involving the sharing and interaction of the single locus incompatibility haplotypes, involved in the recognition or rejection of the self pollen by cells in the stigma. This process ensures out-breeding in certain plant species. [GOC:dph, GOC:pj, GOC:tb]	0.040726702
146	Os03g0266000,Os04g0513000,Os11g0596250,	GO:0009535	chloroplast thylakoid membrane	The pigmented membrane of a chloroplast thylakoid. An example of this component is found in Arabidopsis thaliana. [GOC:lr, GOC:mtg_sensu]	0.040759311
152	Os01g0243300,Os02g0474300,Os11g0107500,	GO:0005777	peroxisome	A small organelle enclosed by a single membrane, and found in most eukaryotic cells. Contains peroxidases and other enzymes involved in a variety of metabolic processes including free radical detoxification, lipid catabolism and biosynthesis, and hydrogen peroxide metabolism. [GOC:pm, PMID:9302272, UniProtKB-KW:KW-0576]	0.044984173

170	Os08g0374701,XLOC_036169,XLOC_040618,	GO:0016310	phosphorylation	The process of introducing a phosphate group into a molecule, usually with the formation of a phosphoric ester, a phosphoric anhydride or a phosphoric amide. [ISBN:0198506732]	0.047365877
153	Os01g0228901,Os03g0329200,Os06g0653200,	GO:0046983	protein dimerization activity	The formation of a protein dimer, a macromolecular structure consists of two noncovalently associated identical or nonidentical subunits. [ISBN:0198506732]	0.048612293
162	Os01g0174400,Os09g0325220,Os09g0510900,	GO:0005515	protein binding	Interacting selectively and non-covalently with any protein or protein complex (a complex of two or more proteins that may include other nonprotein molecules). [GOC:go_curators]	0.054004158
210	Os03g0829100,Os12g0218500,Os12g0222800,	GO:0005515	protein binding	Interacting selectively and non-covalently with any protein or protein complex (a complex of two or more proteins that may include other nonprotein molecules). [GOC:go_curators]	0.054004158
176	Os06g0690900,Os07g0687500,Os08g0290000,	GO:0016853	isomerase activity	Catalysis of the geometric or structural changes within one molecule. Isomerase is the systematic name for any enzyme of EC class 5. [ISBN:0198506732]	0.05862927
97	Os04g0620950,Os07g0645200,Os08g0508700,Os11g0486000,	GO:0043531	ADP binding	Interacting selectively and non-covalently with ADP, adenosine 5'-diphosphate. [GOC:jl]	0.066583884
39	Os03g0158900,Os04g0106500,Os04g0480050,Os05g0111800,Os05g0596500,Os07g0202000,Os09g0327600,Os10g0536450,	GO:0016758	transferase activity, transferring hexosyl groups	Catalysis of the transfer of a hexosyl group from one compound (donor) to another (acceptor). [GOC:jl]	0.075377829
121	Os03g0306302,Os03g0405550,Os11g0586100,XLOC_056911,	GO:0016310	phosphorylation	The process of introducing a phosphate group into a molecule, usually with the formation of a phosphoric ester, a phosphoric anhydride or a phosphoric amide. [ISBN:0198506732]	0.135478514
150	Os01g0705400,Os04g0204200,Os10g0187500,	GO:0016310	phosphorylation	The process of introducing a phosphate group into a molecule, usually with the formation of a phosphoric ester, a phosphoric anhydride or a phosphoric amide. [ISBN:0198506732]	0.135478514
154	Os08g0155550,Os10g0468600,Os11g0542100,	GO:0008270	zinc ion binding	Interacting selectively and non-covalently with zinc (Zn) ions. [GOC:ai]	0.165928264
201	Os01g0391100,Os01g0640700,Os02g0580700,	GO:0016021	integral component of membrane	The component of a membrane consisting of gene products and protein complexes that have some part that penetrates at least one leaflet of the membrane bilayer. This component includes gene products that are buried in the bilayer with no exposure outside the bilayer. [GOC:dos, GOC:go_curators]	0.224665909
172	Os01g0117900,Os11g0245800,Os11g0617800,	GO:0016020	membrane	Double layer of lipid molecules that encloses all cells, and, in eukaryotes, many organelles; may be a single or double lipid bilayer; also includes associated proteins. [GOC:mah, ISBN:0815316194]	0.337142128
191	Os06g0298400,Os09g0112100,Os10g0456950,	GO:0005515	protein binding	Interacting selectively and non-covalently with any protein or protein complex (a complex of two or more proteins that may include other nonprotein molecules). [GOC:go_curators]	0.366106098
10	XLOC_023258,XLOC_032515,XLOC_040714,XLOC_040716,XLOC_060298,XLOC_060300,XLOC_060301,XLOC_060303,XLOC_060304,XLOC_060305,XLOC_060306,XLOC_076740,XLOC_076742,XLOC_077209,	GO:0000002	mitochondrial genome maintenance	The maintenance of the structure and integrity of the mitochondrial genome; includes replication and segregation of the mitochondrial chromosome. [GOC:ai, GOC:vw]	1
99	Os03g0432666,XLOC_008789,XLOC_028252,XLOC_041250,	GO:0000002	mitochondrial genome maintenance	The maintenance of the structure and integrity of the mitochondrial genome; includes replication and segregation of the mitochondrial chromosome. [GOC:ai, GOC:vw]	1

124	Os01g0132200,Os01g0283300,Os01g0343100,Os03g0159400,	GO:0000002	mitochondrial genome maintenance	The maintenance of the structure and integrity of the mitochondrial genome; includes replication and segregation of the mitochondrial chromosome. [GOC:ai, GOC:vw]	1
149	XLOC_012843,XLOC_034808,XLOC_057170,	GO:0000002	mitochondrial genome maintenance	The maintenance of the structure and integrity of the mitochondrial genome; includes replication and segregation of the mitochondrial chromosome. [GOC:ai, GOC:vw]	1
155	Os01g0217100,Os04g0401900,XLOC_039450,	GO:0000002	mitochondrial genome maintenance	The maintenance of the structure and integrity of the mitochondrial genome; includes replication and segregation of the mitochondrial chromosome. [GOC:ai, GOC:vw]	1
157	Os03g0314500,Os05g0531400,Os09g0550400,	GO:0000002	mitochondrial genome maintenance	The maintenance of the structure and integrity of the mitochondrial genome; includes replication and segregation of the mitochondrial chromosome. [GOC:ai, GOC:vw]	1
177	XLOC_001170,XLOC_018220,XLOC_058982,	GO:0000002	mitochondrial genome maintenance	The maintenance of the structure and integrity of the mitochondrial genome; includes replication and segregation of the mitochondrial chromosome. [GOC:ai, GOC:vw]	1
184	Os03g0575500,Os05g0420000,Os07g0187001,	GO:0000002	mitochondrial genome maintenance	The maintenance of the structure and integrity of the mitochondrial genome; includes replication and segregation of the mitochondrial chromosome. [GOC:ai, GOC:vw]	1
188	Os05g0485500,Os07g0140232,XLOC_047717,	GO:0000002	mitochondrial genome maintenance	The maintenance of the structure and integrity of the mitochondrial genome; includes replication and segregation of the mitochondrial chromosome. [GOC:ai, GOC:vw]	1
193	Os04g0638300,XLOC_008728,XLOC_058135,	GO:0000002	mitochondrial genome maintenance	The maintenance of the structure and integrity of the mitochondrial genome; includes replication and segregation of the mitochondrial chromosome. [GOC:ai, GOC:vw]	1
195	Os04g0147200,Os04g0269800,Os05g0119100,	GO:0000002	mitochondrial genome maintenance	The maintenance of the structure and integrity of the mitochondrial genome; includes replication and segregation of the mitochondrial chromosome. [GOC:ai, GOC:vw]	1
196	Os02g0582900,Os08g0428400,Os10g0389500,	GO:0000002	mitochondrial genome maintenance	The maintenance of the structure and integrity of the mitochondrial genome; includes replication and segregation of the mitochondrial chromosome. [GOC:ai, GOC:vw]	1
205	Os02g0142100,Os02g0552700,XLOC_029615,	GO:0000002	mitochondrial genome maintenance	The maintenance of the structure and integrity of the mitochondrial genome; includes replication and segregation of the mitochondrial chromosome. [GOC:ai, GOC:vw]	1
224	XLOC_016626,XLOC_018454,XLOC_020886,XLOC_049042,	GO:0000002	mitochondrial genome maintenance	The maintenance of the structure and integrity of the mitochondrial genome; includes replication and segregation of the mitochondrial chromosome. [GOC:ai, GOC:vw]	1

Table S2. Annotations of the Clusters in the Rice Shoot CeRNA Network

Cluster ID	Nodes in the cluster	GO ID	GO name	GO description	*P* -value
1	Os05g0151000,Os07g0583700,Os04g0388500,Os08g0337300,Os03g0214900, Os03g0111700,Os07g0497100,Os03g0740700,Os04g0691200,Os03g0565300, Os04g0321600,Os06g0645901,Os06g0342500,Os11g0598300,Os11g0217500, Os03g0701900,Os08g0159900,Os04g0661700,Os04g0671800,Os05g0494900, Os03g0853700,Os03g0598200,Os10g0154566,Os01g0314000,Os01g0930200, Os08g0519800,Os06g0134300,Os09g0354900,Os03g0333100,Os11g0660500, Os01g0109700,Os02g0133300,Os11g0579400,Os04g0483600,Os08g0459100, Os04g0568850,Os01g0672300,Os07g0409100,Os02g0231600,Os05g0255600, Os05g0557400,Os03g0694900,Os10g0464100,Os06g0694100,Os01g0234100, Os03g0312600,Os05g0539700,Os03g0775500,Os08g0343300,Os03g0115400, Os10g0447600,Os01g0928300,Os03g0205400,Os07g0613300,Os05g0301600, Os08g0162000,Os05g0128000,Os05g0156800,Os01g0956500,Os02g0631601, Os04g0636800,Os11g0205900,Os01g0655300,Os04g0369100,Os11g0602750, Os04g0635900,Os05g0392700,Os02g0766600,Os06g0111700,Os05g0222200, Os08g0547200,Os12g0624800,Os09g0282300,Os06g0622500,Os04g0687300, Os12g0539333,Os09g0474800,Os06g0602500,Os01g0814000,Os05g0114800, Os02g0785900,Os10g0450900,Os03g0125300,Os03g0395900,Os01g0310100, Os02g0317600,Os06g0724500,Os05g0179800,Os08g0531200,Os04g0614600, Os03g0345100,Os06g0622900,Os12g0498800,Os08g0148267,Os06g0186400, Os04g0471300,Os03g0336300,Os04g0534300,Os11g0557400,Os05g0292200, Os09g0371000,Os03g0843300,Os03g0399600,Os06g0166200,Os03g0800100, Os06g0564300,Os07g0623300,Os08g0460700,Os04g0442700,Os08g0150600, Os05g0177000,Os04g0337300,Os09g0481300,Os03g0812300,Os03g0828500, Os08g0548300,Os08g0157400,Os01g0125700,Os12g0162800,Os01g0939000, Os07g0611600,Os01g0632700,Os07g0164800,Os08g0154225,Os07g0490500, Os03g0685750,Os08g0127300,Os07g0595750,Os06g0605750,Os12g0161900, Os12g0197500,Os11g0197500,Os03g0808100,Os07g0647400,Os05g0417251, Os03g0756500,Os05g0479475,Os08g0494000	GO:0009790	embryo development	The process whose specific outcome is the progression of an embryo from its formation until the end of its embryonic life stage. The end of the embryonic stage is organism-specific. For example, for mammals, the process would begin with zygote formation and end with birth. For insects, the process would begin at zygote formation and end with larval hatching. For plant zygotic embryos, this would be from zygote formation to the end of seed dormancy. For plant vegetative embryos, this would be from the initial determination of the cell or group of cells to form an embryo until the point when the embryo becomes independent of the parent plant. [GOC:go_curators, GOC:isa_complete, GOC:mtg_sensu]	0.000743024
28	Os03g0696300,Os07g0608200,Os03g0647600,Os03g0174900,Os07g0158500, Os12g0618600,Os01g0604700,Os02g0776400,Os12g0154000,	GO:0016602	CCAAT-binding factor complex	A heteromeric transcription factor complex that binds to the CCAAT-box upstream of promoters; in Saccharomyces it activates the transcription of genes in response to growth in a nonfermentable carbon source; consists of four known subunits: HAP2, HAP3, HAP4 and HAP5. [PMID:7828851]	1.29896E-14
113	Os11g0691500,Os09g0471550,Os01g0696800,Os01g0364100,Os02g0624100, Os04g0371000,Os09g0562600,	GO:0030247	polysaccharide binding	Interacting selectively and non-covalently with any polysaccharide, a polymer of many (typically more than 10) monosaccharide residues linked glycosidically. [CHEBI:18154, GOC:mah]	6.13657E-11
237	Os05g0458300,Os05g0458600,Os01g0634500,	GO:0052716	hydroquinone:oxygen oxidoreductase activity	Catalysis of the reaction: 4 hydroquinone + O2 = 4 benzosemiquinone + 4 H2O. [EC:1.10.3.2]	9.87222E-10
40	Os03g0288500,Os04g0466600,Os04g0466700,Os11g0621300,Os02g0139400, Os02g0677800,Os04g0630000,Os06g0215200,Os01g0922600,Os08g0509600, Os06g0663500,Os02g0174100,Os09g0491532,Os08g0531600,	GO:0006351	transcription, DNA-templated	The cellular synthesis of RNA on a template of DNA. [GOC:jl, GOC:txnOH]	3.95129E-08
80	Os05g0355500,Os05g0506000,Os01g0303000,Os03g0687900,Os03g0306800,	GO:0080153	negative regulation of reductive pentose-phosphate cycle	Any process that stops, prevents, or reduces the frequency, rate or extent of the reductive pentose-phosphate cycle. [PMID:17031544, PMID:20399532]	9.52707E-08
290	Os02g0172600,Os08g0390100,Os06g0665800,	GO:0043682	copper-transporting ATPase activity	Catalysis of the transfer of a solute or solutes from one side of a membrane to the other according to the reaction: ATP + H2O + Cu2+ = ADP + phosphate + Cu2+, directly driving the transport of the copper ions across a membrane. [GOC:jl]	9.52707E-08
218	Os01g0839500,Os08g0490900,Os01g0149600,	GO:0046982	protein heterodimerization activity	Interacting selectively and non-covalently with a nonidentical protein to form a heterodimer. [GOC:ai]	1.2065E-07

292	Os01g0639100,Os03g0566800,Os11g0686500,	GO:0035145	exon-exon junction complex	A multi-subunit complex deposited by the spliceosome upstream of messenger RNA exon-exon junctions. The exon-exon junction complex provides a binding platform for factors involved in mRNA export and nonsense-mediated mRNA decay. [PMID:11532962, PMID:11743026]	1.42902E-07
48	Os01g0355600,Os07g0681500,Os11g0551900,Os12g0484900,Os06g0474900, Os10g0463800,Os03g0729500,Os04g0663100,Os01g0510901,Os05g0193900,	GO:0016818	hydrolase activity, acting on acid anhydrides, in phosphorus-containing anhydrides	Catalysis of the hydrolysis of any acid anhydride which contains phosphorus. [GOC:jl]	1.83948E-07
12	Os01g0873300,Os06g0610800,Os05g0401100,Os10g0190100,Os12g0609600, Os07g0171100,Os01g0179600,Os03g0292100,Os05g0542150,Os02g0778500, Os12g0116200,Os06g0103800,Os06g0664300,Os01g0763600,Os10g0346600, Os05g0215300,Os03g0793800,Os04g0645200,Os03g0684700,Os04g0566100, Os04g0640600,Os01g0180300,Os02g0228600,Os08g0200750,Os12g0610250, Os02g0603600,Os04g0657500,Os04g0640500,Os02g0823100,Os08g0506350, Os11g0146800,Os05g0134700,Os06g0562300,Os10g0476000,Os06g0636700, Os10g0488100,Os11g0116550,Os06g0183900,Os12g0143800,	GO:0017119	Golgi transport complex	A complex of proteins that, in vitro, stimulates intra-Golgi transport; a 13S complex, about 800 kDa in size and consists of at least five polypeptides. In yeast, this complex is called the Sec34/35 complex and is composed of eight subunits (Sec34p, Sec35p, Dor1p, Cod1p, Cod2p, Cod3p, Cod4p, and Cod5p). [GOC:krc, PMID:9792665]	9.81819E-07
31	Os07g0603200,Os04g0312100,Os02g0576700,Os02g0102400,Os07g0640000, Os03g0646200,Os04g0459700,Os07g0124500,Os11g0272800,	GO:0009220	pyrimidine ribonucleotide biosynthetic process	The chemical reactions and pathways resulting in the formation of a pyrimidine ribonucleotide, a compound consisting of nucleoside (a pyrimidine base linked to a ribose sugar) esterified with a phosphate group at either the 3' or 5'-hydroxyl group of the sugar. [GOC:go_curators, ISBN:0198506732]	2.12605E-06
93	Os04g0465000,Os03g0356638,Os07g0658300,Os07g0565800,Os08g0442200, Os04g0548100,Os07g0114400,Os05g0391600,Os09g0555700,Os07g0175200, Os05g0568800,Os09g0544900,Os06g0702500,	GO:0005096	GTPase activator activity	Increases the activity of a GTPase, an enzyme that catalyzes the hydrolysis of GTP. [GOC:mah]	2.47507E-06
10	Os01g0383900,Os01g0698000,Os07g0481300,Os12g0228900,Os02g0119600, Os04g0560400,Os01g0231000,Os03g0125650,Os08g0548200,Os10g0147900, Os10g0513900,Os05g0501700,Os06g0693700,Os09g0532000,Os12g0446500, Os07g0163800,Os02g0557500,Os06g0101300,Os01g0753000,Os03g0150700, Os11g0207000,Os03g0328900,Os08g0430800,Os01g0106200,Os10g0502600, Os08g0288500,Os04g0617600,Os04g0626500,Os01g0898300,Os01g0136500, Os10g0420000,Os08g0159800,Os10g0517500,Os01g0183000,Os04g0585200, Os11g0690466,Os12g0110050,Os10g0513400,Os07g0107900,Os05g0100750, Os03g0329200,Os08g0365500,Os03g0379300,Os03g0262000,Os01g0949900, Os05g0149251,Os03g0573750,Os07g0199350,Os12g0102200,Os11g0102600, Os10g0464500,	GO:0034453	microtubule anchoring	Any process in which a microtubule is maintained in a specific location in a cell. [GOC:mah]	4.04933E-06
20	Os02g0564500,Os06g0361500,XLOC_062487,Os05g0106000,Os04g0517350, Os07g0562400,Os11g0615000,Os05g0539400,Os12g0582700,Os01g0832900, Os10g0100500,Os06g0606000,Os07g0678600,Os01g0183300,Os04g0417000, Os06g0548000,Os07g0194100,Os09g0514200,Os08g0207600,Os03g0764800, Os04g0653000,Os12g0527700,Os11g0435500,Os05g0357100,Os05g0489900, Os03g0610900,Os02g0161050,Os05g0186900,Os01g0279000,Os08g0402500, Os02g0806400,Os10g0500400,Os06g0717700,Os07g0564600,Os04g0395700, Os01g0122000,	GO:0004674	protein serine/threonine kinase activity	Catalysis of the reactions: ATP + protein serine = ADP + protein serine phosphate, and ATP + protein threonine = ADP + protein threonine phosphate. [GOC:bf]	6.68899E-06
250	Os03g0273200,Os11g0708100,Os08g0453766,	GO:0009809	lignin biosynthetic process	The chemical reactions and pathways resulting in the formation of lignins, a class of polymers formed by the dehydrogenetive radical polymerization of various phenylpropanoid monomers. [GOC:tair_curators, ISBN:0198547684]	7.42421E-06
143	Os02g0724600,Os09g0551300,Os02g0788400,Os11g0207300,Os01g0705400, Os09g0245500,Os06g0570650,	GO:0008757	S-adenosylmethionine-dependent methyltransferase activity	Catalysis of the transfer of a methyl group from S-adenosyl-L-methionine to a substrate. [GOC:mah]	9.05244E-06
148	Os09g0551900,Os04g0444900,Os04g0686300,Os10g0544500,Os05g0145400,	GO:0016568	chromatin modification	The alteration of DNA, protein, or sometimes RNA, in chromatin, which may result in changing the chromatin structure. [GOC:mah, PMID:20404130]	4.19454E-05

54	Os03g0369800,Os05g0103600,Os08g0459600,Os06g0105400,Os07g0616100,Os02g0559400,	GO:0031408	oxylipin biosynthetic process	The chemical reactions and pathways resulting in the formation of any oxylipin, any of a group of biologically active compounds formed by oxidative metabolism of polyunsaturated fatty acids. [GOC:mah, PMID:11960741]	4.69477E-05
114	Os03g0370500,Os07g0636000,Os03g0778100,Os06g0348800,Os09g0103300,Os07g0141450,Os08g0483200,Os05g0212400,Os12g0102600,Os07g0420000,Os06g0666500,XLOC_036022,Os10g0543500,Os07g0111900,Os03g0342850,	GO:0009982	pseudouridine synthase activity	Catalysis of the reaction: RNA uridine = RNA pseudouridine. Conversion of uridine in an RNA molecule to pseudouridine by rotation of the C1'-N-1 glycosidic bond of uridine in RNA to a C1'-C5. [EC:5.4.99.12, GOC:mah]	7.26318E-05
18	Os08g0153900,Os10g0466000,Os11g0114600,Os10g0492200,Os06g0195800,Os09g0465500,Os02g0173900,Os07g0541600,Os05g0420200,Os11g0432800,Os10g0320400,Os04g0432500,Os01g0519400,Os02g0221500,Os01g0171800,Os09g0413100,Os11g0151200,Os10g0522400,Os04g0524400,Os07g0495100,Os01g0819800,Os02g0178100,Os01g0868000,Os07g0645701,Os12g0605200,Os02g0762800,Os10g0561500,Os10g0390000,Os07g0607800,Os01g0130900,	GO:0048573	photoperiodism, flowering	A change from the vegetative to the reproductive phase as a result of detection of, or exposure to, a period of light or dark of a given length. The length of the period of light or dark required to initiate the change is set relative to a particular duration known as the 'critical day length'. The critical day length varies between species. [GOC:jid, GOC:pj, ISBN:0582015952, ISBN:0697037754, ISBN:0709408862]	9.82216E-05
177	Os02g0566800,Os06g0105700,Os03g0144800,Os10g0442700,	GO:0006486	protein glycosylation	A protein modification process that results in the addition of a carbohydrate or carbohydrate derivative unit to a protein amino acid, e.g. the addition of glycan chains to proteins. [GOC:curators, GOC:pr]	0.000103475
179	Os09g0493400,Os04g0326366,XLOC_070774,Os04g0319600,	GO:0016429	tRNA (adenine-N1-)-methyltransferase activity	Catalysis of the reaction: S-adenosyl-L-methionine + tRNA = S-adenosyl-L-homocysteine + tRNA containing N1-methyladenine. [EC:2.1.1.36]	0.000119546
205	Os03g0433200,Os05g0187500,Os05g0527900,	GO:0045930	negative regulation of mitotic cell cycle	Any process that stops, prevents or reduces the rate or extent of progression through the mitotic cell cycle. [GOC:dph, GOC:go_curators, GOC:tb]	0.000119546
220	Os12g0141900,Os12g0407300,Os10g0553900,	GO:0000374	Group III intron splicing	The splicing of Group III introns. This occurs by a ribozymic mechanism where the intron sequence forms a distinct 3D structure, characteristic of Group III introns, that is involved in catalyzing the splicing reactions, though protein factors are also required in vivo. Splicing occurs by a series of two transesterification reactions begun by a bulged adenosine residue within the intron sequence as the initiating nucleophile. The intron is excised as a lariat. Though very similar in structure and mechanism to Group II introns, Group III introns are smaller and more streamlined and the splice site consensus sequences are not as well conserved. [GOC:krc, PMID:11377794]	0.000119546
284	Os01g0556700,Os08g0416800,Os07g0509800,	GO:0004604	phosphoadenylyl-sulfate reductase (thioredoxin) activity	Catalysis of the reaction: adenosine 3',5'-diphosphate + H(+) + sulfite + thioredoxin disulfide = 3'-phospho-5'-adenylyl sulfate + thioredoxin. Thioredoxin disulfide is the oxidized form of thioredoxin; 3'-phosphoadenosine 5'-phosphosulfate is also known as PAPS. [EC:1.8.4.8, RHEA:11727]	0.000119546
291	Os01g0585700,Os02g0140800,Os06g0137300,	GO:0090239	regulation of histone H4 acetylation	Any process that modulates the rate, frequency, or extent of histone H4 acetylation, the modification of histone H4 by the addition of an acetyl group. [GOC:dph, GOC:tb]	0.000119546
300	Os02g0266000,Os11g0209600,Os01g0311600,	GO:0004640	phosphoribosylanthranilate isomerase activity	Catalysis of the reaction: N-(5-phospho-beta-D-ribosyl)anthranilate = 1-(2-carboxyphenylamino)-1-deoxy-D-ribulose 5-phosphate. [EC:5.3.1.24, RHEA:21543]	0.000119546

73	Os05g0230600,Os02g0472700,Os09g0491852,Os02g0635300,XLOC_068493,	GO:0072546	ER membrane protein complex	A transmembrane protein complex that is involved in protein folding in the endoplasmic reticulum. In S. cerevisiae, it has six members: EMC1, EMC2, AIM27, EMC4, KRE27, and EMC6. [GOC:dgf, PMID:19325107]	0.000159394
116	Os01g0232300,Os02g0294600,Os08g0130900,Os08g0520300,	GO:0042779	tRNA 3'-trailer cleavage	Cleavage of the 3'-end of the pre-tRNA as part of the process of generating the mature 3'-end of the tRNA; may involve endonucleolytic or exonucleolytic cleavage, or both. [GOC:go_curators]	0.000159394
119	Os11g0610900,Os01g0920300,Os03g0266000,XLOC_014220,Os11g0217300,	GO:0047940	glucuronokinase activity	Catalysis of the reaction: D-glucuronate + ATP = 1-phospho-alpha-D-glucuronate + ADP + 2 H(+). [EC:2.7.1.43, RHEA:17008]	0.000159394
124	Os12g0143200,Os04g0204200,Os11g0667700,Os08g0467600,	GO:0080182	histone H3-K4 trimethylation	The modification of histone H3 by addition of three methyl groups to lysine at position 4 of the histone. [GOC:BHF, GOC:se, GOC:tb]	0.000159394
131	Os01g0175500,Os02g0127800,Os07g0164000,Os11g0664800,	GO:0010589	leaf proximal/distal pattern formation	The regionalization process within a leaf by which specific areas of cell differentiation are determined along a proximal/distal axis. [PMID:18398054]	0.000159394
153	Os01g0211600,Os04g0495400,Os10g0370000,Os07g0112100,	GO:0016052	carbohydrate catabolic process	The chemical reactions and pathways resulting in the breakdown of carbohydrates, any of a group of organic compounds based of the general formula Cx(H2O)y. [ISBN:0198506732]	0.000159394
155	Os01g0917500,Os03g0648300,Os11g0183800,Os10g0189100,	GO:0019388	galactose catabolic process	The chemical reactions and pathways resulting in the breakdown of galactose, the aldohexose galacto-hexose. [ISBN:0198506732]	0.000159394
159	Os03g0845000,Os05g0387200,Os01g0929500,Os06g0111400,	GO:0046506	sulfolipid biosynthetic process	The chemical reactions and pathways resulting in the formation of sulfolipid, a compound containing a sulfonic acid residue joined by a carbon-sulfur bond to a lipid. [PMID:9751667]	0.000159394
160	Os01g0314100,Os07g0282300,Os04g0490500,Os12g0607000,	GO:0003826	alpha-ketoacid dehydrogenase activity	Catalysis of an oxidation-reduction (redox) reaction involving an alpha-ketoacid. [GOC:mah]	0.000159394
304	Os03g0785800,Os01g0909400,Os06g0497500,Os02g0121100,	GO:0043457	regulation of cellular respiration	Any process that modulates the frequency, rate or extent of cellular respiration, the enzymatic release of energy from organic compounds. [GOC:jl]	0.000159394

305	Os02g0816900,Os02g0793300,Os08g0106300,Os08g0162400,	GO:0008239	dipeptidyl-peptidase activity	Catalysis of the hydrolysis of N-terminal dipeptides from a polypeptide chain. [GOC:mb]	0.000159394
166	Os05g0410200,Os06g0116800,Os01g0844500,Os09g0461500,	GO:0008219	cell death	Any biological process that results in permanent cessation of all vital functions of a cell. A cell should be considered dead when any one of the following molecular or morphological criteria is met: (1) the cell has lost the integrity of its plasma membrane; (2) the cell, including its nucleus, has undergone complete fragmentation into discrete bodies (frequently referred to as \apoptotic bodies\"); and/or (3) its corpse (or its fragments) have been engulfed by an adjacent cell in vivo." [GOC:mah, GOC:mtg_apoptosis]	0.000161149
76	Os03g0832400,Os07g0521600,Os04g0682900,Os08g0520100,Os02g0597200,	GO:0000404	loop DNA binding	Interacting selectively and non-covalently with DNA containing a loop. A loop occurs when DNA contains a large insertion or deletion that causes a region of unpaired single-stranded DNA to loop out, while the rest of the DNA is in a paired double-stranded configuration. [GOC:elh, PMID:16781730]	0.000199243
77	Os04g0416100,Os04g0653200,Os01g0741900,Os04g0535600,Os08g0421825,	GO:0071370	cellular response to gibberellin stimulus	Any process that results in a change in state or activity of a cell (in terms of movement, secretion, enzyme production, gene expression, etc.) as a result of a gibberellin stimulus. [GOC:mah]	0.000199243
97	Os03g0159700,Os10g0116800,Os01g0836400,Os03g0843900,Os09g0474000,	GO:0004649	poly(ADP-ribose) glycohydrolase activity	Catalysis of the hydrolysis of poly(ADP-ribose) at glycosidic (1''-2') linkage of ribose-ribose bond to produce free ADP-ribose. [EC:3.2.1.143]	0.000199243
109	Os01g0597800,Os09g0532900,Os11g0138400,Os01g0217800,Os03g0695600,	GO:0010294	abscisic acid glucosyltransferase activity	Catalysis of the reaction: (+)-abscisate + UDP-D-glucose = abscisic acid glucose ester + UDP. [DOI:10.1016/j.tetasy.2004.11.062]	0.000199243
188	Os03g0681300,Os02g0579600,Os07g0583600,XLOC_013369,Os05g0176500, Os07g0298700,	GO:0033615	mitochondrial proton-transporting ATP synthase complex assembly	The aggregation, arrangement and bonding together of a proton-transporting ATP synthase in the mitochondrial inner membrane. [GOC:mah]	0.000199243
57	Os06g0687900,Os07g0496200,Os01g0934100,Os02g0592200,Os03g0272900, Os02g0503500,Os05g0118000,	GO:0051287	NAD binding	Interacting selectively and non-covalently with nicotinamide adenine dinucleotide, a coenzyme involved in many redox and biosynthetic reactions; binding may be to either the oxidized form, NAD+, or the reduced form, NADH. [GOC:ai]	0.000213834
150	Os03g0597200,Os10g0580800,Os04g0534000,Os10g0403700,	GO:0010027	thylakoid membrane organization	A process that is carried out at the cellular level which results in the assembly, arrangement of constituent parts, or disassembly of the thylakoid membrane. [GOC:dph, GOC:jl, GOC:mah, GOC:tb]	0.000216944
239	Os05g0435400,Os06g0231300,Os10g0427300,	GO:0009408	response to heat	Any process that results in a change in state or activity of a cell or an organism (in terms of movement, secretion, enzyme production, gene expression, etc.) as a result of a heat stimulus, a temperature stimulus above the optimal temperature for that organism. [GOC:lr]	0.000219978

108	Os01g0828100,Os08g0526350,Os02g0224300,Os03g0336700,Os06g0651000,	GO:0044237	cellular metabolic process	The chemical reactions and pathways by which individual cells transform chemical substances. [GOC:go_curators]	0.000220911
211	Os03g0800050,Os12g0613200,XLOC_062234,	GO:0010452	histone H3-K36 methylation	The modification of histone H3 by addition of a methyl group to lysine at position 36 of the histone. [GOC:tb]	0.000239082
257	XLOC_012445,Os03g0810600,Os01g0141700,	GO:0019725	cellular homeostasis	Any process involved in the maintenance of an internal steady state at the level of the cell. [GOC:isa_complete, GOC:jl, ISBN:0395825172]	0.000239082
121	Os12g0406000,XLOC_034785,Os05g0361200,XLOC_015320,Os10g0442100, XLOC_060644,XLOC_040535,	GO:0004325	ferrochelatase activity	Catalysis of the reaction: protoheme = Fe(2+) + protoporphyrin IX. [EC:4.99.1.1, RHEA:22587]	0.000239082
132	Os01g0690600,Os05g0489100,Os06g0698300,XLOC_033689,	GO:0004656	procollagen-proline 4-dioxygenase activity	Catalysis of the reaction: procollagen L-proline + 2-oxoglutarate + O2 = procollagen trans-4-hydroxy-L-proline + succinate + CO2. [EC:1.14.11.2]	0.000239082
178	Os01g0691050,Os04g0514600,XLOC_013940,Os12g0297400,	GO:0050776	regulation of immune response	Any process that modulates the frequency, rate or extent of the immune response, the immunological reaction of an organism to an immunogenic stimulus. [GOC:ai]	0.000239082
197	Os01g0225400,Os02g0184100,Os12g0576300,	GO:0003864	3-methyl-2-oxobutanoate hydroxymethyltransferase activity	Catalysis of the reaction: 5,10-methylenetetrahydrofolate + 3-methyl-2-oxobutanoate = tetrahydrofolate + 2-dehydropantoate. [EC:2.1.2.11]	0.000239082
200	Os02g0440000,Os11g0149300,Os03g0586400,	GO:0008836	diaminopimelate decarboxylase activity	Catalysis of the reaction: meso-2,6-diaminopimelate + H(+) = L-lysine + CO(2). [EC:4.1.1.20, RHEA:15104]	0.000239082
204	Os07g0236300,Os10g0411700,Os02g0700500,	GO:0006407	rRNA export from nucleus	The directed movement of rRNA from the nucleus to the cytoplasm; the rRNA is usually in the form of ribonucleoproteins. [GOC:ma, GOC:mah]	0.000239082
243	Os08g0224000,Os11g0572800,Os03g0684000,	GO:0043985	histone H4-R3 methylation	The modification of histone H4 by addition of a methyl group to arginine at position 3 of the histone. [GOC:mah]	0.000239082

259	Os02g0600400,Os07g0130500,Os02g0664200,	GO:0003980	UDP-glucose:glycoprotein glucosyltransferase activity	Catalysis of the addition of UDP-glucose on to asparagine-linked (N-linked) oligosaccharides of the form Man7-9GlcNAc2 on incorrectly folded glycoproteins. [GOC:al, PMID:10764828]	0.000239082
280	Os04g0397100,Os10g0445600,Os10g0497100,	GO:0034214	protein hexamerization	The formation of a protein hexamer, a macromolecular structure consisting of six noncovalently associated identical or nonidentical subunits. [GOC:ecd]	0.000239082
295	Os01g0851100,Os03g0268100,Os01g0752200,	GO:0003860	3-hydroxyisobutyryl-CoA hydrolase activity	Catalysis of the reaction: 3-hydroxy-2-methylpropanoyl-CoA + H2O = CoA + 3-hydroxy-2-methylpropanoate. [EC:3.1.2.4]	0.000239082
53	Os01g0814800,Os03g0363600,Os01g0358300,Os04g0396800,Os04g0481300, Os09g0542200,	GO:0090322	regulation of superoxide metabolic process	Any process that modulates the rate, frequency, or extent of superoxide metabolism, the chemical reactions and pathways involving superoxide, the superoxide anion O2- (superoxide free radical), or any compound containing this species. [GOC:tb]	0.000239091
42	Os04g0490700,Os12g0270200,Os06g0670000,Os12g0623500,Os07g0540100, Os12g0134700,Os01g0174000,	GO:0008265	Mo-molybdopterin cofactor sulfurase activity	Catalysis of the sulfurylation of the desulfo form of molybdenum cofactor (MoCo), a cofactor required for the activity of some enzymes, such as aldehyde oxidase. [GOC:mah, PMID:11549764]	0.00027894
43	XLOC_026644,Os03g0743900,Os03g0195400,Os08g0450800,Os12g0571900, Os04g0585400,Os02g0107000,Os11g0668100,	GO:0070206	protein trimerization	The formation of a protein trimer, a macromolecular structure consisting of three noncovalently associated identical or nonidentical subunits. [GOC:hjd]	0.00027894
186	Os03g0438400,Os10g0498600,Os06g0484950,Os06g0218200,Os05g0220600, Os09g0448500,Os01g0565900,	GO:0080140	regulation of jasmonic acid metabolic process	Any process that modulates the frequency, rate or extent of the chemical reactions and pathways involving jasmonic acid. [GOC:dhl]	0.00027894
47	Os08g0135800,Os12g0120100,Os11g0120300,Os12g0573000,Os06g0309200, XLOC_071610,Os11g0172150,	GO:0003743	translation initiation factor activity	Functions in the initiation of ribosome-mediated translation of mRNA into a polypeptide. [ISBN:0198506732]	0.000287484
49	Os06g0103300,Os02g0562400,Os11g0162000,Os01g0580100,Os04g0407900, Os07g0187300,Os03g0412300,Os02g0696900,	GO:0019395	fatty acid oxidation	The removal of one or more electrons from a fatty acid, with or without the concomitant removal of a proton or protons, by reaction with an electron-accepting substance, by addition of oxygen or by removal of hydrogen. [ISBN:0198506732, MetaCyc:FAO-PWY]	0.000318789
167	Os02g0267000,Os09g0346500,Os05g0215033,XLOC_056004,	GO:0055035	plastid thylakoid membrane	The lipid bilayer membrane of any thylakoid within a plastid. [GOC:jid, GOC:rph]	0.000358609

193	Os06g0691400,Os05g0112200,Os08g0485500,	GO:0010178	IAA-amino acid conjugate hydrolase activity	Catalysis of the cleavage of the amide bond between IAA (auxin) and the conjugated amino acid. [GOC:tb]	0.000358609
194	Os05g0223000,Os11g0552500,Os04g0509000,	GO:0010068	protoderm histogenesis	The formation of the primary meristem or meristematic tissue that gives rise to the epidermis. [GOC:tb, ISBN:0471245208]	0.000358609
262	Os03g0138700,Os07g0613200,Os03g0857100,	GO:0043201	response to leucine	Any process that results in a change in state or activity of a cell or an organism (in terms of movement, secretion, enzyme production, gene expression, etc.) as a result of a leucine stimulus. [GOC:mlg]	0.000358609
39	Os01g0391100,Os01g0689451,Os02g0580700,Os12g0156400,Os03g0156500,Os06g0123000,Os03g0313000,Os02g0736300,Os03g0819900,	GO:0022904	respiratory electron transport chain	A process in which a series of electron carriers operate together to transfer electrons from donors such as NADH and FADH2 to any of several different terminal electron acceptors to generate a transmembrane electrochemical gradient. [GOC:mtg_electron_transport, ISBN:0716720094]	0.000358637
64	XLOC_073642,Os04g0455401,XLOC_030020,Os11g0586100,Os05g0511400,Os09g0364800,Os03g0669200,Os06g0710700,XLOC_054224,Os12g0288266,Os09g0533650,Os05g0582500,XLOC_066861,XLOC_063811,	GO:0009991	response to extracellular stimulus	Any process that results in a change in state or activity of a cell or an organism (in terms of movement, secretion, enzyme production, gene expression, etc.) as a result of an extracellular stimulus. [GOC:go_curators]	0.000358637
125	Os02g0120000,Os12g0417100,Os08g0559000,Os09g0343200,Os10g0577900,Os12g0209000,Os09g0128400,Os08g0531000,Os01g0816000,	GO:0004366	glycerol-3-phosphate O-acyltransferase activity	Catalysis of the reaction: acyl-CoA + sn-glycerol 3-phosphate = CoA + 1-acyl-sn-glycerol 3-phosphate. [EC:2.3.1.15]	0.000358637
202	Os01g0158600,Os08g0111200,XLOC_061908,	GO:0006680	glucosylceramide catabolic process	The chemical reactions and pathways resulting in the breakdown of glucosylceramides, any compound formed by the replacement of the glycosidic hydroxyl group of a cyclic form of glucose by a ceramide group. [GOC:ai]	0.000398454
66	Os01g0120400,Os01g0886000,XLOC_067155,Os04g0667700,Os03g0621650,Os07g0181200,	GO:0030029	actin filament-based process	Any cellular process that depends upon or alters the actin cytoskeleton, that part of the cytoskeleton comprising actin filaments and their associated proteins. [GOC:mah]	0.000398454
71	Os02g0313450,Os12g0228400,Os02g0122800,Os08g0326600,Os04g0169100,	GO:0003922	GMP synthase (glutamine-hydrolyzing) activity	Catalysis of the reaction: ATP + xanthosine 5'-phosphate + L-glutamine + H2O = AMP + diphosphate + GMP + L-glutamate. [EC:6.3.5.2]	0.000398454
72	Os05g0220500,Os05g0417000,Os06g0218800,Os08g0502400,Os06g0182100,	GO:0009799	specification of symmetry	The establishment of an organism's body plan or part of an organism such that a similar arrangement in form and relationship of parts around a common axis, or around each side of a plane is created. [GOC:go_curators]	0.000398454

79	Os02g0186400,Os10g0572100,Os04g0298200,Os06g0714000,Os01g0540800,	GO:0008509	anion transmembrane transporter activity	Catalysis of the transfer of a negatively charged ion from one side of a membrane to the other. [GOC:dgf, GOC:mtg_transport, ISBN:0815340729]	0.000398454
83	Os06g0127000,Os09g0570000,Os04g0671700,Os08g0272200,Os03g0225200,	GO:0019172	glyoxalase III activity	Catalysis of the reaction: methylglyoxal + H2O = D-lactate. [MetaCyc:GLYOXIII-RXN]	0.000398454
98	Os08g0557600,Os11g0158400,Os07g0223100,Os07g0566200,Os01g0827300,	GO:0046480	galactolipid galactosyltransferase activity	Catalysis of the reaction: 2 mono-beta-D-galactosyldiacylglycerol = alpha-D-galactosyl-beta-D-galactosyldiacylglycerol + 1,2-diacylglycerol. [EC:2.4.1.184]	0.000398454
110	Os03g0723000,Os05g0580000,Os07g0510800,Os03g0784400,Os02g0602900,	GO:0010170	glucose-1-phosphate adenylyltransferase complex	Complex that catalyzes the synthesis of ADP-glucose and pyrophosphate from glucose-1-phosphate and ATP. In plants, the complex is a heterotetramer composed of two types of subunits (small and large). In bacteria, the enzyme complex is composed of four identical subunits. [GOC:tb, PMID:12748181]	0.000398454
115	Os01g0951200,Os04g0687900,Os02g0158800,Os07g0535100,Os05g0139200,	GO:0004588	orotate phosphoribosyltransferase activity	Catalysis of the reaction: orotidine 5'-phosphate + diphosphate = orotate + 5-phospho-alpha-D-ribose 1-diphosphate. [EC:2.4.2.10]	0.000398454
164	Os06g0142800,Os07g0111700,Os01g0805400,Os01g0835700,Os01g0717650,	GO:0016277	[myelin basic protein]-arginine N-methyltransferase activity	Catalysis of the reaction: S-adenosyl-L-methionine + (myelin basic protein)-arginine = S-adenosyl-L-homocysteine + (myelin basic protein)-N(omega)-methyl-arginine. [EC:2.1.1.126]	0.000398454
165	Os02g0541325,Os02g0258250,Os05g0163300,Os03g0750700,Os12g0158300,	GO:0006580	ethanolamine metabolic process	The chemical reactions and pathways involving ethanolamine (2-aminoethanol), an important water-soluble base of phospholipid (phosphatidylethanolamine). [CHEBI:16000, GOC:jl, ISBN:01928006X]	0.000398454
13	Os02g0702000,Os07g0168000,Os03g0121700,Os03g0265400,Os03g0861700, Os01g0711800,Os08g0117100,Os04g0691600,Os06g0237000,Os04g0438300, Os01g0361000,Os02g0184300,Os11g0466966,Os11g0544000,Os08g0560800,	GO:0009570	chloroplast stroma	The space enclosed by the double membrane of a chloroplast but excluding the thylakoid space. It contains DNA, ribosomes and some temporary products of photosynthesis. [ISBN:0198547684]	0.000420454
25	Os03g0667201,Os09g0246300,Os03g0271500,Os02g0308800,Os03g0245700, Os03g0826200,Os02g0170000,Os06g0574100,Os12g0615400,Os04g0397500, Os07g0229900,Os04g0513000,XLOC_010433,Os06g0147100,XLOC_010819, Os10g0140200,XLOC_073131,Os09g0391800,Os03g0326500,Os05g0179900, Os11g0606400,Os12g0538700,Os02g0782800,Os12g0182200,Os03g0227300, XLOC_061072,Os03g0806700,Os11g0547800,Os07g0110000,Os11g0201175,	GO:0008610	lipid biosynthetic process	The chemical reactions and pathways resulting in the formation of lipids, compounds soluble in an organic solvent but not, or sparingly, in an aqueous solvent. [GOC:go_curators]	0.000471174
140	Os09g0452900,Os06g0554700,Os12g0230600,Os11g0215100,	GO:0048531	beta-1,3-galactosyltransferase activity	Catalysis of the transfer of a galactose residue from a donor molecule to an oligosaccharide, forming a beta-1,3-linkage. [PMID:11551958]	0.000478126

137	Os09g0567700,Os07g0693900,Os07g0481400,XLOC_025724,	GO:0005847	mRNA cleavage and polyadenylation specificity factor complex	A multisubunit complex that binds to the canonical AAUAAA hexamer and to U-rich upstream sequence elements on the pre-mRNA, thereby stimulating the otherwise weakly active and nonspecific polymerase to elongate efficiently RNAs containing a poly(A) signal. [PMID:14749727]	0.000478126
170	Os02g0680500,Os03g0651000,Os04g0455800,XLOC_065652,	GO:0009249	protein lipoylation	The addition of a lipoyl group to an amino acid residue in a protein. [GOC:mah]	0.000478126
198	Os07g0103100,Os01g0847700,Os09g0441400,	GO:0004965	G-protein coupled GABA receptor activity	Combining with the amino acid gamma-aminobutyric acid (GABA, 4-aminobutyrate) and transmitting the signal across the membrane by activating an associated G-protein; promotes the exchange of GDP for GTP on the alpha subunit of a heterotrimeric G-protein complex. [GOC:ai, GOC:bf, IUPHAR_RECEPTOR:1276, Wikipedia:GABAB_receptor]	0.000478126
213	Os01g0270100,Os05g0457800,Os03g0793000,	GO:0090447	glycerol-3-phosphate 2-O-acyltransferase activity	Catalysis of the reaction: an acyl-CoA + sn-glycerol 3-phosphate = CoA + a 2-acyl-sn-glycerol 3-phosphate. [EC:2.3.1.198]	0.000478126
227	Os01g0102600,Os09g0515400,Os04g0473150,	GO:0004765	shikimate kinase activity	Catalysis of the reaction: ATP + shikimate = 3-phosphoshikimate + ADP + 2 H(+). [EC:2.7.1.71, RHEA:13124]	0.000478126
231	Os05g0428400,Os10g0474900,Os03g0810500,	GO:0046577	long-chain-alcohol oxidase activity	Catalysis of the reaction: 2 long-chain alcohol + O2 = 2 long-chain aldehyde + 2 H2O. [EC:1.1.3.20]	0.000478126
255	Os05g0390300,Os04g0470150,Os02g0771100,	GO:0000152	nuclear ubiquitin ligase complex	A ubiquitin ligase complex found in the nucleus. [GOC:mah]	0.000478126
276	Os03g0712300,Os05g0100600,Os05g0142500,	GO:0031372	UBC13-MMS2 complex	A heterodimeric ubiquitin conjugating enzyme complex that catalyzes assembly of K63-linked polyubiquitin chains, which act as a signal to promote error-free DNA postreplication repair; in Saccharomyces the complex comprises Ubc13p and Mms2p. [GOC:mah, PMID:15772086]	0.000478126
281	Os04g0220501,Os04g0452000,Os06g0471100,	GO:0010161	red light signaling pathway	The series of molecular signals initiated upon sensing of red light by a photoreceptor molecule. Red light is electromagnetic radiation of wavelength of 580-700nm. An example of this response is seen at the beginning of many plant species developmental stages. These include germination, and the point when cotyledon expansion is triggered. In certain species these processes take place in response to absorption of red light by the pigment molecule phytochrome, but the signal can be reversed by exposure to far red light. During the initial phase the phytochrome molecule is only present in the red light absorbing form, but on absorption of red light it changes to a far red light absorbing form, triggering progress through development. An immediate short period of exposure to far red light entirely returns the pigment to its [illegible]	0.000478126
289	Os04g0692300,Os06g0588900,Os08g0191800,	GO:0004831	tyrosine-tRNA ligase activity	Catalysis of the reaction: L-tyrosine + ATP + tRNA(Tyr) = L-tyrosyl-tRNA(Tyr) + AMP + diphosphate + 2 H(+). [EC:6.1.1.1, RHEA:10223]	0.000478126

60	Os11g0432900,Os03g0123300,Os07g0615900,Os07g0657600,Os05g0476100,Os12g0103150,	GO:0032877	positive regulation of DNA endoreduplication	Any process that activates or increases the frequency, rate or extent of DNA endoreduplication. [GOC:mah]	0.000478135
21	Os01g0355800,Os07g0467600,Os08g0520400,Os03g0775300,Os04g0388601,Os02g0323300,Os04g0422300,Os02g0140200,Os05g0319700,Os05g0597200,Os02g0529600,Os02g0821400,	GO:0034074	marneral synthase activity	Catalysis of the reaction: oxidosqualene = marneral. [GOC:cb, http://www.wiley-vch.de/contents/jc_2002/2006/z503420_s.pdf, PMID:16425307, PMID:18033581]	0.000478183
44	Os07g0164100,Os07g0646000,Os10g0580300,Os03g0657000,Os12g0131150,Os02g0100700,Os09g0482720,Os03g0169000,Os08g0100700,Os05g0172300,Os05g0255800,Os03g0346900,	GO:0007160	cell-matrix adhesion	The binding of a cell to the extracellular matrix via adhesion molecules. [GOC:hb]	0.000478183
16	Os03g0395600,Os09g0306700,Os02g0510000,Os09g0440300,Os01g0872700,Os05g0346500,Os04g0471400,Os07g0227800,Os03g0385900,Os04g0665500,Os03g0243300,Os03g0167800,Os08g0386300,	GO:0006517	protein deglycosylation	The removal of sugar residues from a glycosylated protein. [GOC:mah]	0.000518031
87	Os09g0321500,Os02g0557800,Os02g0564300,Os01g0919800,Os07g0406800,Os12g0548100,Os01g0852400,Os12g0569900,Os10g0543800,Os08g0520850,Os02g0174000,Os12g0583500,Os02g0121400,	GO:0000799	nuclear condensin complex	A multisubunit protein complex that plays a central role in the condensation of chromosomes that remain in the nucleus. [GOC:elh]	0.000518031
58	Os03g0326100,Os09g0567300,Os01g0654200,Os03g0278366,Os02g0139300,Os07g0471050,Os05g0143700,	GO:0043903	regulation of symbiosis, encompassing mutualism through parasitism	Any process that modulates the frequency, rate or extent of symbiosis, an interaction between two organisms living together in more or less intimate association. [GOC:jl]	0.000557813
94	Os02g0110700,Os04g0602800,Os06g0258900,XLOC_037328,Os01g0730300,Os03g0187400,Os08g0527300,Os11g0707300,	GO:0016832	aldehyde-lyase activity	Catalysis of the cleavage of a C-C bond in a molecule containing a hydroxyl group and a carbonyl group to form two smaller molecules, each being an aldehyde or a ketone. [http://www.mercksource.com/]	0.000557813
99	Os03g0750100,Os03g0650200,Os01g0866600,Os02g0752701,Os03g0625800,	GO:0051879	Hsp90 protein binding	Interacting selectively and non-covalently with Hsp90 proteins, any of a group of heat shock proteins around 90kDa in size. [GOC:ai]	0.000597633
101	Os03g0224200,Os12g0597300,Os06g0585900,Os02g0545000,Os01g0636600,	GO:0017137	Rab GTPase binding	Interacting selectively and non-covalently with Rab protein, any member of the Rab subfamily of the Ras superfamily of monomeric GTPases. [GOC:mah]	0.000597633
149	Os08g0318500,Os11g0264600,XLOC_057842,Os03g0372700,	GO:0048354	mucilage biosynthetic process involved in seed coat development	The chemical reactions and pathways resulting in the formation of mucilage that occur as part of seed coat development; mucilage is normally synthesized during seed coat development. [GOC:dph, GOC:jid, GOC:tb]	0.000597633

208	Os05g0305600,Os05g0521500,Os09g0378300,Os03g0274700,Os09g0508951,	GO:0004823	leucine-tRNA ligase activity	Catalysis of the reaction: L-leucine + ATP + tRNA(Leu) = AMP + diphosphate + 2 H(+) + Leu-tRNA(Leu). [EC:6.1.1.4, RHEA:11691]	0.000597633
260	Os04g0440000,Os03g0161800,Os07g0179400,	GO:0010309	acireductone dioxygenase [iron(II)-requiring] activity	Catalysis of the reaction: 1,2-dihydroxy-5-(methylthio)pent-1-en-3-one + O(2) = 4-methylthio-2-oxobutanoate + formate + H(+). [EC:1.13.11.54, RHEA:24507]	0.000597633
267	Os02g0196600,Os07g0225000,Os07g0634900,	GO:0043682	copper-transporting ATPase activity	Catalysis of the transfer of a solute or solutes from one side of a membrane to the other according to the reaction: ATP + H2O + Cu2+ = ADP + phosphate + Cu2+, directly driving the transport of the copper ions across a membrane. [GOC:jl]	0.000597633
293	Os01g0742500,Os01g0873700,Os07g0577700,	GO:0004775	succinate-CoA ligase (ADP-forming) activity	Catalysis of the reaction: ATP + succinate + CoA = ADP + succinyl-CoA + phosphate. [EC:6.2.1.5]	0.000597633
11	Os09g0460000,Os11g0549700,Os04g0623066,Os07g0512200,Os02g0809800, Os03g0765200,Os12g0255200,Os02g0720700,Os03g0759700,Os11g0537300, Os01g0299500,Os03g0651300,Os03g0200700,Os07g0204000,Os02g0203500,	GO:0005776	autophagic vacuole	A double-membrane-bounded compartment in which endogenous cellular material is sequestered; known as autophagosome in yeast. [ISBN:0198547684, PMID:11099404]	0.000597729
130	Os01g0641100,Os02g0193500,Os07g0688800,Os09g0500600,	GO:0003917	DNA topoisomerase type I activity	Catalysis of a DNA topological transformation by transiently cleaving one DNA strand at a time to allow passage of another strand; changes the linking number by +1 per catalytic cycle. [PMID:8811192]	0.000637463
36	Os03g0761100,Os09g0327500,Os04g0662900,Os07g0679300,Os03g0211100, Os03g0700450,Os04g0528300,Os01g0883100,	GO:0016480	negative regulation of transcription from RNA polymerase III promoter	Any process that stops, prevents, or reduces the frequency, rate or extent of transcription from an RNA polymerase III promoter. [GOC:go_curators]	0.000637488
52	Os06g0700300,Os03g0852500,Os05g0429400,Os03g0279600,Os03g0826500, Os10g0407100,	GO:0004049	anthranilate synthase activity	Catalysis of the reaction: chorismate + L-glutamine = anthranilate + pyruvate + L-glutamate. [EC:4.1.3.27]	0.000717131
67	Os11g0482000,Os02g0192700,Os06g0320500,Os12g0583300,Os08g0476300, Os02g0673000,	GO:0009768	photosynthesis, light harvesting in photosystem I	After a photon of light is absorbed by one of the many chlorophyll molecules, in one of the light-harvesting complexes of an antenna on photosystem I, some of the absorbed energy is transferred to the pair of chlorophyll molecules in the reaction center. [GOC:jid, ISBN:0716731363, ISBN:0816017360]	0.000717131
175	Os01g0882300,Os04g0639100,XLOC_068207,Os06g0649800,	GO:0008725	DNA-3-methyladenine glycosylase activity	Catalysis of the reaction: DNA containing 3-methyladenine + H2O = DNA with abasic site + 3-methyladenine. This reaction is the hydrolysis of DNA by cleavage of the N-C1' glycosidic bond between the damaged DNA 3-methyladenine and the deoxyribose sugar to remove the 3-methyladenine, leaving an abasic site. [EC:3.2.2.20, GOC:elh, PMID:10872450, PMID:9224623]	0.000717131

296	Os03g0318600,Os11g0191300,Os03g0673700,	GO:0007143	female meiosis	A cell cycle process comprising the steps by which a cell progresses through the nuclear division phase of a meiotic cell cycle in the female germline. [GOC:dph, GOC:ems, GOC:mah]	0.000717131
256	Os12g0485800,Os09g0525400,XLOC_002340,	GO:0016272	prefoldin complex	A multisubunit chaperone that is capable of delivering unfolded proteins to cytosolic chaperonin, which it acts as a cofactor for. In humans, the complex is a heterohexamer of two PFD-alpha and four PFD-beta type subunits. In Saccharomyces cerevisiae, it also acts in the nucleus to regulate the rate of elongation by RNA polymerase II via a direct effect on histone dynamics. [GOC:jl, PMID:17384227, PMID:24068951, PMID:9630229]	0.00071716
285	Os08g0249100,XLOC_046758,Os11g0237900,	GO:0002238	response to molecule of fungal origin	Any process that results in a change in state or activity of an organism (in terms of movement, secretion, enzyme production, gene expression, etc.) as a result of a stimulus by molecules of fungal origin such as chito-octamer oligosaccharide. [GOC:rl, GOC:sm]	0.00071716
92	Os11g0148700,Os12g0233800,Os01g0128250,Os07g0626300,Os10g0550200, Os04g0308000,Os07g0211900,Os01g0233900,Os02g0833300,Os02g0116400, Os10g0389200,Os04g0580001,Os06g0538200,Os08g0505900,Os12g0562900, Os04g0582700,Os06g0158300,Os03g0832900,	GO:0042493	response to drug	Any process that results in a change in state or activity of a cell or an organism (in terms of movement, secretion, enzyme production, gene expression, etc.) as a result of a drug stimulus. A drug is a substance used in the diagnosis, treatment or prevention of a disease. [GOC:jl]	0.000717274
127	Os01g0267100,Os03g0243350,Os05g0557600,Os01g0772700,Os09g0296900, Os12g0631600,Os03g0748800,Os08g0223900,Os04g0269900,Os02g0828100, Os03g0777000,Os03g0813700,Os11g0485200,Os11g0167800,XLOC_017361, Os11g0302500,Os03g0766100,Os01g0253900,Os01g0717000,	GO:0010686	tetracyclic triterpenoid biosynthetic process	The chemical reactions and pathways resulting in the formation of tetracyclic triterpenoid compounds, terpenoids with six isoprene units and 4 carbon rings. [GOC:tair_curators]	0.000717274
234	XLOC_030613,Os02g0777700,XLOC_070228,	GO:0003774	motor activity	Catalysis of movement along a polymeric molecule such as a microfilament or microtubule, coupled to the hydrolysis of a nucleoside triphosphate. [GOC:mah, ISBN:0815316194]	0.000717274
134	Os09g0570500,Os04g0629300,Os02g0274700,Os11g0697001,	GO:0008270	zinc ion binding	Interacting selectively and non-covalently with zinc (Zn) ions. [GOC:ai]	0.000747373
105	Os02g0287000,Os02g0829200,Os01g0265200,Os05g0553300,Os01g0913900,	GO:0005347	ATP transmembrane transporter activity	Catalysis of the transfer of ATP, adenosine triphosphate, from one side of a membrane to the other. [GOC:ai]	0.000796781
117	Os02g0608300,Os08g0478800,Os01g0773100,Os01g0691500,Os06g0234200,	GO:0004347	glucose-6-phosphate isomerase activity	Catalysis of the reaction: D-glucose 6-phosphate = D-fructose 6-phosphate. [EC:5.3.1.9]	0.000796781
118	Os01g0610800,Os03g0577500,Os04g0658100,Os03g0148800,	GO:0070461	SAGA-type complex	A histone acetyltransferase complex that acetylates nucleosomal H3 and H2B and is required for the expression of a subset of Pol II-transcribed genes. The budding yeast complex includes the acetyltransferase Gcn5p, several proteins of the Spt and Ada families, and several TBP-associate proteins (TAFs); analogous complexes in other species have analogous compositions, and usually contain homologs of the yeast proteins. [GOC:mah, PMID:10637607, PMID:17337012]	0.000796781

133	Os04g0411800,Os09g0506800,Os06g0618300,Os05g0268500,	GO:0009554	megasporogenesis	The process in which the megasporocyte undergoes meiosis, giving rise to four haploid megaspores in the nucellus. [GOC:mtg_plant, GOC:tb]	0.000796781
135	Os04g0209200,Os09g0464400,Os03g0285900,Os05g0120100,	GO:0008517	folic acid transporter activity	Enables the directed movement of folic acid (pteroylglutamic acid) into, out of or within a cell, or between cells. Folic acid is widely distributed as a member of the vitamin B complex and is essential for the synthesis of purine and pyrimidines. [GOC:ai]	0.000796781
156	Os11g0479000,Os12g0479400,Os02g0221600,Os05g0299500,	GO:0008285	negative regulation of cell proliferation	Any process that stops, prevents or reduces the rate or extent of cell proliferation. [GOC:go_curators]	0.000796781
176	Os02g0193000,Os01g0278600,Os04g0602700,Os01g0134700,	GO:0002098	tRNA wobble uridine modification	The process in which a uridine in position 34 of a tRNA is post-transcriptionally modified. [GOC:hjd, ISBN:155581073X]	0.000796781
24	Os02g0105100,Os03g0625700,Os01g0925700,Os01g0832000,Os06g0210200, Os05g0215600,Os03g0279000,Os10g0510300,Os01g0152500,Os02g0684000,	GO:0004046	aminoacylase activity	Catalysis of the reaction: an N-acyl-L-amino acid + H2O = a carboxylate + an L-amino acid. [EC:3.5.1.14]	0.000796829
30	XLOC_071313,Os07g0543000,XLOC_005426,XLOC_035144,Os10g0498100, Os11g0592000,XLOC_076779,XLOC_054355,XLOC_030886,XLOC_021897, XLOC_030038,	GO:0080027	response to herbivore	Any process that results in a change in state or activity of a cell or an organism (in terms of movement, secretion, enzyme production, gene expression, etc.) as a result of a stimulus from a herbivore. [PMID:18987211]	0.00083662
196	Os04g0432000,Os04g0601500,Os10g0499400,	GO:0070300	phosphatidic acid binding	Interacting selectively and non-covalently with phosphatidic acid, any of a class of glycerol phosphate in which both the remaining hydroxyl groups of the glycerol moiety are esterified with fatty acids. [CHEBI:16337, GOC:jp, ISBN:0198506732]	0.00083662
252	Os01g0223600,Os06g0671800,Os03g0645000,	GO:0052546	cell wall pectin metabolic process	The chemical reactions and pathways involving pectin, a polymer containing a backbone of alpha-1,4-linked D-galacturonic acid residues, as part of the organization and biogenesis of the cell wall. [GOC:ai]	0.00083662
19	Os04g0610000,Os06g0217300,Os06g0638900,Os07g0599000,Os03g0650400, Os01g0757500,Os05g0427300,Os10g0405100,Os10g0134700,Os11g0236932, Os04g0181901,	GO:0000060	protein import into nucleus, translocation	A protein transport process that contributes to protein import into the nucleus, and that results in the vectorial transfer of a cargo-carrier protein complex through the nuclear pore complex from the cytoplasmic side to the nucleoplasmic side of the nuclear envelope. [GOC:curators, ISBN:0198506732, PMID:14570049, PMID:9126736]	0.000876494
3	Os10g0160600,XLOC_047346,Os02g0622200,XLOC_019886,Os03g0837000, Os09g0514400,Os01g0512200,Os07g0601900,Os03g0756200,Os03g0684500, XLOC_048281,XLOC_051599,Os07g0695400,XLOC_068645,Os02g0144300, XLOC_021491,Os11g0433200,Os02g0555000,Os11g0525800,Os02g0504100, Os11g0428500,XLOC_002113,Os02g0592600,Os07g0647800,Os01g0935700, Os12g0107500,Os03g0644000,Os03g0835800,Os04g0401300,XLOC_031943, Os05g0318600,Os01g0321700,Os04g0558700,Os05g0564000,Os06g0648200, Os07g0643800,Os07g0466200,Os11g0452400,Os10g0135500,Os03g0683900, Os08g0562600,XLOC_053044,Os01g0392800,Os01g0910200,Os11g0565400, XLOC_069348,Os10g0410900,Os04g0418100,Os09g0109800,Os10g0556500, Os09g0506900,Os05g0568100,XLOC_025619,Os01g0949700,Os06g0218500, Os06g0560300,Os09g0314400,Os11g0167200,Os01g0963600,Os02g0122200, [illegible]	GO:0006378	mRNA polyadenylation	The enzymatic addition of a sequence of 40-200 adenylyl residues at the 3' end of a eukaryotic mRNA primary transcript. [ISBN:0198506732]	0.000932449

154	Os05g0442550,Os08g0289400,Os02g0642600,Os12g0588400,	GO:0000712	resolution of meiotic recombination intermediates	The cleavage and rejoining of intermediates, such as Holliday junctions, formed during meiotic recombination to produce two intact molecules in which genetic material has been exchanged. [GOC:elh, PMID:11733053]	0.00095608
181	Os04g0228100,Os09g0242800,Os02g0714200,Os08g0199200,	GO:0047334	diphosphate-fructose-6-phosphate 1-phosphotransferase activity	Catalysis of the reaction: fructose-6-phosphate + diphosphate = phosphate + fructose-1,6-bisphosphate. [EC:2.7.1.90, MetaCyc:2.7.1.90-RXN]	0.00095608
183	Os04g0398800,Os09g0407300,Os04g0384800,Os06g0183100,	GO:0071203	WASH complex	A protein complex that localizes at the surface of endosomes, where it recruits and activates the Arp2/3 complex to induce actin polymerization. In human, the WASH complex is composed of F-actin-capping protein subunits alpha and beta, WASH1, FAM21, KIAA1033, KIAA0196 and CCDC53. [GOC:sp, PMID:19922875]	0.00095608
219	Os01g0861000,Os08g0246400,Os07g0120900,	GO:0006122	mitochondrial electron transport, ubiquinol to cytochrome c	The transfer of electrons from ubiquinol to cytochrome c that occurs during oxidative phosphorylation, mediated by the multisubunit enzyme known as complex III. [ISBN:0716731363]	0.000956099
65	Os03g0619600,Os11g0157600,Os02g0137800,Os01g0195400,Os02g0109900,Os05g0563600,Os07g0641200,Os11g0687100,Os10g0495416,Os03g0826400,Os12g0169700,Os08g0192800,	GO:0070207	protein homotrimerization	The formation of a protein homotrimer, a macromolecular structure consisting of three noncovalently associated identical subunits. [GOC:hjd]	0.000956156
90	Os01g0193900,Os02g0791800,Os01g0531200,Os12g0609200,Os05g0467300,Os04g0172400,Os01g0877600,XLOC_066757,Os03g0789600,Os07g0686500,Os03g0704200,Os05g0501350,Os02g0679200,XLOC_075298,	GO:0017053	transcriptional repressor complex	A protein complex that possesses activity that prevents or downregulates transcription. [GOC:mah]	0.000956156
5	Os08g0480100,Os10g0440500,Os03g0233500,XLOC_045455,Os01g0202601,Os08g0494400,Os07g0178600,Os12g0175400,Os01g0740700,Os04g0390000,Os06g0287500,XLOC_027543,Os08g0500500,Os01g0667100,Os03g0259300,Os04g0512300,Os01g0135900,Os02g0681900,Os07g0661600,Os01g0830700,Os01g0861900,Os07g0641800,Os01g0174900,Os02g0786200,Os02g0264700,Os06g0505302,Os05g0100500,Os05g0466800,Os06g0644500,Os01g0629400,Os07g0412100,Os01g0283700,Os02g0719600,Os01g0196800,Os04g0414100,Os09g0498000,Os08g0300200,Os01g0729200,Os05g0426066,Os05g0137400,Os07g0169600,Os01g0907300,Os01g0611100,Os01g0742400,Os01g0664500,Os05g0549700,XLOC_051624,Os05g0515400,Os06g0148000,Os04g0505700,XLOC_011019,Os02g0119700,XLOC_059443,Os12g0618800,Os10g0212100,Os10g0497900,Os01g0876400,Os03g0264400,Os01g0743300,Os08g0451400,[illegible]	GO:0009787	regulation of abscisic acid-activated signaling pathway	Any process that modulates the frequency, rate or extent of abscisic acid (ABA) signaling. [GOC:lr]	0.001022543
244	Os08g0158400,Os03g0744800,Os06g0536200,	GO:0046520	sphingoid biosynthetic process	The chemical reactions and pathways resulting in the formation of sphingoids, any of a class of compounds comprising sphinganine and its homologues and stereoisomers, and derivatives of these compounds. [ISBN:0198506732]	0.001075569
112	Os05g0595100,Os01g0813400,Os02g0782600,Os12g0231000,Os02g0180500,Os11g0156200,Os11g0195000,	GO:0004106	chorismate mutase activity	Catalysis of the reaction: chorismate = prephenate. [EC:5.4.99.5, RHEA:13900]	0.00111536
38	XLOC_029375,Os02g0792100,Os12g0175700,XLOC_020442,Os12g0210200,Os12g0168400,Os01g0694100,Os10g0485300,XLOC_000267,	GO:0000304	response to singlet oxygen	Any process that results in a change in state or activity of a cell or an organism (in terms of movement, secretion, enzyme production, gene expression, etc.) as a result of a singlet oxygen stimulus. Singlet oxygen is a dioxygen (O2) molecule in which two 2p electrons have similar spin. Singlet oxygen is more highly reactive than the form in which these electrons are of opposite spin, and it is produced in mutant chloroplasts lacking carotenoids and by leukocytes during metabolic burst. [GOC:krc, ISBN:0124325653, ISBN:0198506732]	0.001194981

14	Os05g0495100,Os01g0105700,Os03g0698800,Os01g0562600,Os05g0542200,Os02g0536400,Os06g0662000,Os05g0546300,Os01g0121100,Os11g0151700,Os09g0280500,Os03g0823000,Os03g0725800,Os03g0104000,Os08g0356800,Os06g0291500,XLOC_067697,Os05g0581100,Os01g0850100,Os05g0191500,Os06g0726300,Os01g0578700,XLOC_001874,Os01g0961300,Os04g0417800,Os03g0682100,Os07g0211200,Os02g0238500,XLOC_064796,XLOC_019586,Os10g0183500,XLOC_034236,Os07g0642600,Os03g0330200,Os04g0677100,Os01g0680950,	GO:0045792	negative regulation of cell size	Any process that reduces cell size. [GOC:go_curators]	0.001235306
139	Os01g0253300,Os02g0571800,Os03g0209500,Os04g0483200,	GO:0048471	perinuclear region of cytoplasm	Cytoplasm situated near, or occurring around, the nucleus. [GOC:jid]	0.001274621
171	Os07g0688500,Os03g0320100,Os10g0357850,Os11g0167500,	GO:0031123	RNA 3'-end processing	Any process involved in forming the mature 3' end of an RNA molecule. [GOC:mah]	0.001274621
278	Os01g0675100,Os02g0192300,Os12g0409800,	GO:0051920	peroxiredoxin activity	Catalysis of the reaction: 2 R'-SH + ROOH = R'-S-S-R' + H2O + ROH. [EC:1.11.1.15]	0.001314479
86	Os04g0108101,XLOC_063523,Os01g0292900,Os02g0582300,Os09g0478400,Os02g0599151,Os03g0828300,Os09g0329200,Os09g0441000,Os10g0480500,Os01g0857650,Os02g0728700,Os03g0339900,Os08g0558800,Os01g0268800,Os01g0167700,Os04g0272200,Os03g0392300,XLOC_040486,Os08g0337700,Os04g0451200,Os10g0406900,Os06g0695800,Os03g0391400,Os12g0158800,XLOC_037810,Os05g0227600,Os03g0183600,Os01g0905300,XLOC_021899,Os11g0275000,Os04g0444300,XLOC_031093,Os08g0288050,Os09g0455500,Os09g0549450,Os11g0141400,Os12g0275500,XLOC_001879,	GO:0071368	cellular response to cytokinin stimulus	Any process that results in a change in state or activity of a cell (in terms of movement, secretion, enzyme production, gene expression, etc.) as a result of a cytokinin stimulus. [GOC:mah]	0.001315003
75	Os01g0875400,Os06g0315900,Os04g0672200,Os10g0316400,Os08g0465800,	GO:0004351	glutamate decarboxylase activity	Catalysis of the reaction: L-glutamate = 4-aminobutanoate + CO2. [EC:4.1.1.15]	0.001394033
85	Os09g0499600,Os02g0608200,Os08g0191700,Os11g0194900,Os11g0170400,	GO:0004462	lactoylglutathione lyase activity	Catalysis of the reaction: (R)-S-lactoylglutathione = glutathione + methylglyoxal. [EC:4.4.1.5, RHEA:19072]	0.001394033
96	Os04g0433600,Os10g0479600,Os02g0167500,Os04g0103100,Os01g0347000,	GO:0030150	protein import into mitochondrial matrix	The import of proteins across the outer and inner mitochondrial membranes into the matrix. Unfolded proteins enter the mitochondrial matrix with a chaperone protein; the information required to target the precursor protein from the cytosol to the mitochondrial matrix is contained within its N-terminal matrix-targeting sequence. Translocation of precursors to the matrix occurs at the rare sites where the outer and inner membranes are close together. [ISBN:0716731363]	0.001394033
111	Os03g0386000,Os05g0358400,Os09g0419200,Os08g0544800,Os06g0235850,Os02g0598400,Os04g0591232,	GO:0016621	cinnamoyl-CoA reductase activity	Catalysis of the reaction: cinnamaldehyde + CoA + NADP+ = cinnamoyl-CoA + NADPH + H+. [EC:1.2.1.44]	0.001394033
275	Os02g0170500,Os12g0236225,XLOC_071461,	GO:0016602	CCAAT-binding factor complex	A heteromeric transcription factor complex that binds to the CCAAT-box upstream of promoters; in Saccharomyces it activates the transcription of genes in response to growth in a nonfermentable carbon source; consists of four known subunits: HAP2, HAP3, HAP4 and HAP5. [PMID:7828851]	0.001434063

29	Os09g0397200,Os06g0241100,Os10g0370400,Os08g0439100,Os02g0179800,Os01g0304300,Os02g0173500,Os12g0568800,Os05g0163700,Os01g0821700,XLOC_037969,Os07g0154400,Os02g0598350,Os06g0235200,Os04g0607600,Os08g0551300,XLOC_056566,XLOC_045026,Os02g0173000,Os04g0580800,Os05g0573400,Os01g0923000,	GO:0006656	phosphatidylcholine biosynthetic process	The chemical reactions and pathways resulting in the formation of phosphatidylcholines, any of a class of glycerophospholipids in which the phosphatidyl group is esterified to the hydroxyl group of choline. [ISBN:0198506732]	0.001513703
9	Os04g0647300,Os07g0108900,Os05g0313500,Os03g0295800,Os05g0586500,Os04g0667600,Os06g0216000,Os02g0190000,Os03g0107900,Os01g0542000,Os01g0267200,Os05g0371200,Os08g0234200,Os02g0712700,Os03g0107300,Os05g0336000,Os05g0541100,Os07g0122400,Os01g0921000,	GO:0000502	proteasome complex	A large multisubunit complex which catalyzes protein degradation, found in eukaryotes, archaea and some bacteria. In eukaryotes, this complex consists of the barrel shaped proteasome core complex and one or two associated proteins or complexes that act in regulating entry into or exit from the core. [GOC:rb, http://en.wikipedia.org/wiki/Proteasome]	0.001536184
45	Os01g0248600,Os10g0114300,Os03g0712900,Os12g0170600,Os08g0190300,Os03g0381200,Os01g0121200,Os07g0501800,Os12g0106900,Os02g0565200,Os05g0443400,Os03g0745000,Os02g0833600,	GO:0008290	F-actin capping protein complex	A heterodimer consisting of alpha and beta subunits that binds to and caps the barbed ends of actin filaments, thereby regulating the polymerization of actin monomers but not severing actin filaments. [GOC:go_curators, ISBN:0198599560]	0.001553351
258	Os07g0268800,Os11g0141000,Os03g0296700,	GO:0009966	regulation of signal transduction	Any process that modulates the frequency, rate or extent of signal transduction. [GOC:sm]	0.001553351
15	Os01g0931000,Os01g0859400,Os03g0654700,Os12g0278800,Os03g0241900,Os08g0155550,Os03g0151900,Os01g0583100,Os08g0342300,Os11g0131800,Os04g0191600,Os04g0447700,Os02g0453300,Os05g0137500,Os02g0722800,Os12g0137500,Os09g0346700,Os07g0106100,Os03g0294100,Os07g0164500,Os03g0194900,Os08g0105100,Os06g0726200,Os03g0728100,Os01g0718300,Os07g0417800,Os07g0102000,Os04g0368800,Os03g0603500,Os01g0839900,Os11g0685500,Os12g0410150,Os02g0580500,Os09g0319701,Os12g0456200,Os10g0213800,Os03g0678800,Os11g0111800,Os06g0153000,	GO:0009029	tetraacyldisaccharide 4'-kinase activity	Catalysis of the reaction: 2,3-bis(3-hydroxytetradecanoyl)-D-glucosaminyl-(1->6)-beta-D-2,3-bis(3-hydroxytetradecanoyl)-beta-D-glucosaminyl 1-phosphate + ATP = ADP + 2 H(+) + lipid IV(a). [EC:2.7.1.130, RHEA:20703]	0.001554094
7	Os04g0197100,Os11g0175800,Os01g0952600,Os02g0638400,Os09g0536200,Os06g0297800,Os08g0440300,Os06g0121200,Os10g0154700,Os06g0638000,Os01g0736551,Os09g0563950,Os08g0120600,Os07g0631700,Os08g0102700,Os08g0428100,Os01g0247700,Os07g0572100,Os09g0482840,Os08g0483900,	GO:0009341	beta-galactosidase complex	A protein complex that possesses beta-galactosidase activity, i.e. catalyzes the hydrolysis of terminal non-reducing beta-D-galactose residues in beta-D-galactosides. In E. coli, the complex is a homotetramer; dimeric and hexameric beta-galactosidase complexes have been observed in other species. [BRENDA:3.2.1.2, EC:3.2.1.23, PMID:15950161]	0.00159334
268	Os04g0674300,Os06g0608700,Os07g0638100,	GO:0004332	fructose-bisphosphate aldolase activity	Catalysis of the reaction: D-fructose 1,6-bisphosphate = glycerone phosphate + D-glyceraldehyde-3-phosphate. [EC:4.1.2.13]	0.001672773
199	Os07g0688100,Os12g0601800,Os03g0237600,	GO:0016114	terpenoid biosynthetic process	The chemical reactions and pathways resulting in the formation of terpenoids, any member of a class of compounds characterized by an isoprenoid chemical structure. [GOC:ai]	0.001792186
141	Os03g0259900,Os03g0708750,Os12g0279100,Os02g0189800,	GO:0010541	acropetal auxin transport	The unidirectional movement of auxin from the base towards the apex of an organ, including the shoot, leaf, primary root, or lateral root. [PMID:10677441]	0.001911474
161	Os04g0490800,Os11g0242400,Os11g0432400,Os11g0127800,	GO:0009507	chloroplast	A chlorophyll-containing plastid with thylakoids organized into grana and frets, or stroma thylakoids, and embedded in a stroma. [ISBN:0471245208]	0.001957735

69	Os10g0500500,Os04g0459800,Os05g0116000,Os04g0550200,Os04g0682600,XLOC_025912,	GO:0004004	ATP-dependent RNA helicase activity	Catalysis of the reaction: ATP + H2O = ADP + phosphate; this reaction drives the unwinding of an RNA helix. [EC:3.6.1.3, GOC:jl]	0.001991
32	Os01g0896800,Os03g0668900,Os05g0373700,Os05g0597100,Os07g0475800,Os07g0617000,Os11g0130300,Os09g0103400,Os02g0714300,Os06g0270925,	GO:0008097	5S rRNA binding	Interacting selectively and non-covalently with 5S ribosomal RNA, the smallest RNA constituent of a ribosome. [GOC:jl, ISBN:0321000382]	0.001991
251	Os03g0170701,Os10g0404900,Os11g0221000,	GO:0000976	transcription regulatory region sequence-specific DNA binding	Interacting selectively and non-covalently with a specific sequence of DNA that is part of a regulatory region that controls transcription of that section of the DNA. The transcribed region might be described as a gene, cistron, or operon. [GOC:txnOH]	0.002030982
261	Os03g0674600,Os04g0593600,Os03g0371000,	GO:0009610	response to symbiotic fungus	Any process that results in a change in state or activity of a cell or an organism (in terms of movement, secretion, enzyme production, gene expression, etc.) as a result of a stimulus from a symbiotic fungus, a fungus living in close physical association with another organism. [GOC:hb, ISBN:0198506732]	0.002030982
294	Os06g0223700,Os07g0164600,Os09g0251100,	GO:0001104	RNA polymerase II transcription cofactor activity	Interacting selectively and non-covalently with an RNA polymerase II (RNAP II) regulatory transcription factor and also with the RNAP II basal transcription machinery in order to modulate transcription. Cofactors generally do not bind DNA, but rather mediate protein-protein interactions between regulatory transcription factors and the basal RNAP II transcription machinery. [GOC:txnOH, PMID:10213677, PMID:16858867]	0.002030982
26	Os08g0227100,Os02g0714500,Os07g0109400,Os01g0926200,Os11g0579700,Os06g0687500,Os09g0564800,Os03g0283100,Os01g0108200,	GO:0010731	protein glutathionylation	The protein modification process in which a glutathione molecule is added to a protein amino acid through a disulfide linkage. [GOC:BHF, GOC:dph, GOC:rl, GOC:tb]	0.002150109
195	Os07g0507300,Os11g0588400,XLOC_054077,	GO:0006972	hyperosmotic response	Any process that results in a change in state or activity of a cell or an organism (in terms of movement, secretion, enzyme production, gene expression, etc.) as a result of detection of, or exposure to, a hyperosmotic environment, i.e. an environment with a higher concentration of solutes than the organism or cell. [GOC:jl, PMID:12142009]	0.002150708
100	Os03g0210600,Os10g0565401,Os07g0107800,Os10g0535600,Os03g0809400,	GO:0007169	transmembrane receptor protein tyrosine kinase signaling pathway	A series of molecular signals initiated by the binding of an extracellular ligand to a receptor on the surface of the target cell where the receptor possesses tyrosine kinase activity, and ending with regulation of a downstream cellular process, e.g. transcription. [GOC:ceb, GOC:signaling]	0.002189926
33	Os03g0635000,Os04g0461100,Os03g0336000,Os01g0589000,Os03g0795200,Os09g0380200,Os03g0697200,Os10g0413400,Os02g0564400,Os01g0321300,Os08g0549600,Os07g0666300,Os05g0279850,Os12g0219700,Os08g0538800,Os05g0272800,Os03g0264600,Os02g0122700,Os03g0738900,Os03g0184300,Os02g0554800,Os09g0363400,Os05g0179950,Os10g0146901,Os06g0114366,Os02g0717800,Os04g0429450,Os08g0113000,	GO:0017038	protein import	The directed movement of proteins into a cell or organelle. [GOC:ai]	0.00223032
41	Os06g0255700,Os07g0434500,Os09g0436500,Os12g0131000,Os03g0861800,Os06g0166100,Os05g0420500,	GO:0003676	nucleic acid binding	Interacting selectively and non-covalently with any nucleic acid. [GOC:jl]	0.002289506

147	Os07g0648233,Os08g0276000,Os02g0567000,Os06g0604200,Os05g0466100,	GO:0070290	NAPE-specific phospholipase D activity	Catalysis of the release of N-acylethanolamine from N-acyl-phosphatidylethanolamine (NAPE) to generate N-acylethanolamine (NAE). [GOC:elh, PMID:14634025, PMID:15878693]	0.002388819
8	Os12g0176500,Os01g0660550,Os06g0597400,Os12g0288400,Os08g0433600, Os01g0913000,Os04g0391500,Os02g0175100,Os02g0715300,Os02g0106800, Os02g0564000,Os02g0276400,Os10g0184250,Os11g0181900,Os06g0731100, Os09g0432900,Os02g0706400,Os06g0621800,Os06g0247900,Os01g0889000, Os12g0443700,Os08g0112800,XLOC_045400,Os09g0556200,Os01g0187400, Os05g0470900,Os02g0450000,XLOC_075749,Os01g0301300,Os05g0200340, Os07g0165100,Os05g0548800,	GO:0080148	negative regulation of response to water deprivation	Any process that stops, prevents, or reduces the frequency, rate or extent of a response to water deprivation. Response to water deprivation is a change in state or activity of a cell or an organism (in terms of movement, secretion, enzyme production, gene expression, etc.) as a result of a water deprivation stimulus, prolonged deprivation of water. [PMID:18835996]	0.002389533
23	Os02g0272900,Os07g0183700,Os02g0232600,Os01g0776800,Os01g0793400, XLOC_025812,Os03g0411000,XLOC_047345,XLOC_076778,Os04g0337201, Os01g0825900,XLOC_027447,Os07g0227400,Os01g0128100,Os12g0138800, Os03g0215800,Os05g0437800,Os01g0681400,XLOC_054892,XLOC_040461, Os01g0934300,Os12g0298950,Os08g0105000,Os09g0510200,Os10g0468500, Os09g0371700,Os04g0252200,Os01g0910800,Os02g0805250,Os02g0474300, Os09g0115600,Os01g0650000,Os07g0438700,Os04g0433200,Os12g0582800, Os11g0545000,Os03g0168300,Os05g0363200,Os11g0107500,Os07g0496900, Os07g0474700,Os08g0431800,Os05g0346100,Os01g0653800,Os02g0720300, Os02g0222100,Os05g0415600,Os05g0437200,Os11g0594200,Os05g0482400, Os07g0503900,Os12g0580950,Os07g0605800,Os05g0479900,Os02g0254700, Os06g0730800,Os11g0549635,Os01g0557100,Os08g0528601,Os01g0899500, [illegible]	GO:0070652	HAUS complex	A protein complex that localizes to interphase centrosomes and to mitotic spindle tubules and regulates mitotic spindle assembly and centrosome integrity; in human, the complex consists of eight subunits, some of which are homologous to subunits of the Drosophila Augmin complex. [PMID:19427217]	0.002430763
236	Os02g0589700,Os01g0281100,Os02g0734300,	GO:0008374	O-acyltransferase activity	Catalysis of the transfer of an acyl group to an oxygen atom on the acceptor molecule. [GOC:ai]	0.00250846
55	Os02g0315600,Os06g0604300,Os06g0625500,Os12g0289800,Os07g0690900, Os07g0692600,	GO:0051920	peroxiredoxin activity	Catalysis of the reaction: 2 R'-SH + ROOH = R'-S-S-R' + H2O + ROH. [EC:1.11.1.15]	0.002627387
302	Os11g0487700,Os12g0611300,Os01g0740350,Os07g0630800,Os12g0632900, Os12g0601400,	GO:0009963	positive regulation of flavonoid biosynthetic process	Any process that activates or increases the frequency, rate or extent of the chemical reactions and pathways resulting in the formation of flavonoids. [GOC:tb]	0.002627387
22	Os02g0678200,Os03g0100300,Os10g0159800,Os01g0151100,Os01g0558200, Os01g0113350,Os10g0485500,Os04g0395100,Os12g0548401,Os07g0507600,	GO:0051205	protein insertion into membrane	The process that results in the incorporation of a protein into a biological membrane. [GOC:ai]	0.002786401
95	Os10g0442000,Os09g0456700,Os09g0542700,Os06g0260500,Os04g0450300,	GO:0000159	protein phosphatase type 2A complex	A protein complex that has protein serine/threonine phosphatase activity that is polycation-stimulated (PCS), being directly stimulated by protamine, polylysine, or histone H1; it constitutes a subclass of several enzymes activated by different histones and polylysine, and consists of catalytic, scaffolding, and regulatory subunits. The catalytic and scaffolding subunits form the core enzyme, and the holoenzyme also includes the regulatory subunit. [GOC:mah, ISBN:0198547684, PMID:17245430]	0.002786512
272	Os04g0382100,Os07g0187700,Os09g0379600,	GO:0006817	phosphate ion transport	The directed movement of phosphate into, out of or within a cell, or between cells, by means of some agent such as a transporter or pore. [GOC:krc]	0.002866469
2	Os03g0238200,Os01g0104800,Os03g0711800,Os10g0437600,Os04g0337800, Os05g0393200,Os06g0711600,Os02g0822900,Os12g0105600,Os08g0529000, Os01g0788500,Os07g0270900,Os01g0355500,Os12g0152600,Os03g0324300, Os03g0802500,Os03g0811600,Os12g0516700,Os03g0646900,Os01g0667200, Os06g0334400,Os11g0490900,Os02g0832400,Os07g0230600,Os08g0550400, Os01g0931100,Os01g0879300,Os01g0580800,Os03g0130400,Os01g0155500, Os05g0552100,Os01g0951400,Os02g0823800,Os02g0701600,Os06g0335101, Os01g0278200,Os04g0398000,Os02g0282900,Os02g0255500,Os09g0481650, Os10g0537600,Os01g0342750,Os01g0965500,Os06g0267500,Os02g0526500, Os03g0324200,Os07g0103200,Os05g0474600,Os04g0542000,Os01g0819700, Os02g0187800,Os03g0217900,Os01g0773800,Os08g0428200,Os09g0487900, Os04g0105200,Os05g0576600,Os10g0465700,Os07g0644100,Os12g0554000, [illegible]	GO:0071281	cellular response to iron ion	Any process that results in a change in state or activity of a cell (in terms of movement, secretion, enzyme production, gene expression, etc.) as a result of an iron ion stimulus. [GOC:mah]	0.002963299

287	Os03g0724700,Os06g0691000,Os08g0143500,	GO:0016926	protein desumoylation	The process in which a SUMO protein (small ubiquitin-related modifier) is cleaved from its target protein. [GOC:jl, PMID:11265250]	0.002985786
62	Os07g0609766,Os10g0124500,Os10g0456200,Os05g0134300,Os05g0164800,Os01g0743732,	GO:0005385	zinc ion transmembrane transporter activity	Catalysis of the transfer of zinc (Zn) ions from one side of a membrane to the other. [GOC:dgf]	0.003104475
129	Os07g0133700,Os10g0496900,Os04g0122000,XLOC_035326,XLOC_020272,XLOC_022927,	GO:0005528	FK506 binding	Interacting selectively and non-covalently with the immunosuppressant FK506. [GOC:jl]	0.003105093
103	Os02g0794300,Os04g0403300,Os04g0430200,Os10g0362700,Os09g0480100,	GO:0004556	alpha-amylase activity	Catalysis of the endohydrolysis of (1->4)-alpha-D-glucosidic linkages in polysaccharides containing three or more alpha-(1->4)-linked D-glucose units. [EC:3.2.1.1]	0.003184077
123	Os02g0648300,Os10g0562700,Os01g0232200,Os04g0376400,	GO:0006032	chitin catabolic process	The chemical reactions and pathways resulting in the breakdown of chitin, a linear polysaccharide consisting of beta-(1->4)-linked N-acetyl-D-glucosamine residues. [GOC:jl, ISBN:0198506732]	0.003343281
245	Os06g0649900,Os07g0282500,Os06g0215400,	GO:0004177	aminopeptidase activity	Catalysis of the hydrolysis of N-terminal amino acid residues from in a polypeptide chain. [GOC:jl, ISBN:0198506732]	0.00334368
126	Os01g0518800,XLOC_065020,Os04g0350000,Os04g0579700,Os06g0650800,	GO:0015095	magnesium ion transmembrane transporter activity	Catalysis of the transfer of magnesium (Mg) ions from one side of a membrane to the other. [GOC:dgf]	0.003661251
6	Os04g0338900,Os09g0369800,Os07g0602900,Os09g0272000,Os03g0666200,Os02g0550300,Os05g0211100,Os10g0563800,Os09g0354300,Os05g0401500,Os02g0705600,Os02g0704600,Os05g0569300,Os06g0652300,Os10g0518800,Os01g0182900,Os01g0276400,Os07g0620300,Os02g0612900,Os07g0538000,Os08g0497900,Os08g0282400,Os09g0550000,Os09g0338400,Os03g0284800,Os01g0780900,Os04g0169300,Os03g0831300,Os11g0141550,Os02g0570300,Os01g0768200,Os02g0772500,Os08g0559300,Os05g0444200,Os11g0582300,Os06g0232000,Os01g0770200,Os02g0625300,Os01g0958000,Os04g0510500,Os04g0423400,Os09g0327300,Os12g0121300,Os10g0536450,Os06g0589600,Os11g0525200,Os09g0518500,Os03g0148000,Os03g0741600,Os10g0441900,Os02g0697300,Os04g0484900,Os06g0651600,Os11g0123400,Os10g0562200,Os02g0136000,Os01g0248701,Os04g0478000,Os04g0641400,Os08g0537900,[illegible]	GO:0010084	specification of organ axis polarity	The process in which the polarity of an organ axis is specified. [GOC:tb]	0.003745766
89	Os04g0626700,Os05g0102900,Os04g0621650,Os01g0239300,XLOC_048059,XLOC_046102,Os02g0325100,XLOC_022758,Os07g0623000,Os07g0476500,Os02g0275100,Os04g0454600,Os08g0434201,Os04g0162701,Os03g0203000,XLOC_047552,	GO:0017004	cytochrome complex assembly	The aggregation, arrangement and bonding together of a cytochrome complex. A cytochrome complex is a protein complex in which at least one of the proteins is a cytochrome, i.e. a heme-containing protein involved in catalysis of redox reactions. [GOC:jl, GOC:mah]	0.003819599
298	Os08g0417000,Os09g0511900,XLOC_047013,	GO:0051213	dioxygenase activity	Catalysis of an oxidation-reduction (redox) reaction in which both atoms of oxygen from one molecule of O2 are incorporated into the (reduced) product(s) of the reaction. The two atoms of oxygen may be distributed between two different products. [DOI:10.1016/S0040-4020(03)00944-X, GOC:bf, http://www.onelook.com/]	0.003901425

162	Os01g0813100,Os08g0231100,Os02g0503100,Os05g0121600,Os06g0319700,Os09g0343400,Os05g0135500,Os10g0554200,Os01g0566500,	GO:0015112	nitrate transmembrane transporter activity	Catalysis of the transfer of nitrate ions (NO3-) from one side of a membrane to the other. [GOC:ai]	0.003938726
59	Os06g0687450,Os07g0621800,Os10g0477000,Os03g0859300,Os09g0345000,Os02g0805000,	GO:0005488	binding	The selective, non-covalent, often stoichiometric, interaction of a molecule with one or more specific sites on another molecule. [GOC:ceb, GOC:mah, ISBN:0198506732]	0.004027722
50	Os05g0130600,Os09g0540500,Os07g0508900,Os01g0283300,Os12g0273300,Os05g0127500,XLOC_040000,	GO:0001104	RNA polymerase II transcription cofactor activity	Interacting selectively and non-covalently with an RNA polymerase II (RNAP II) regulatory transcription factor and also with the RNAP II basal transcription machinery in order to modulate transcription. Cofactors generally do not bind DNA, but rather mediate protein-protein interactions between regulatory transcription factors and the basal RNAP II transcription machinery. [GOC:txnOH, PMID:10213677, PMID:16858867]	0.004058081
271	Os03g0786250,Os12g0270900,Os10g0555200,	GO:0008146	sulfotransferase activity	Catalysis of the transfer of a sulfate group from 3'-phosphoadenosine 5'-phosphosulfate to the hydroxyl group of an acceptor, producing the sulfated derivative and 3'-phosphoadenosine 5'-phosphate. [EC:2.8.2, GOC:curators]	0.004059212
37	Os06g0690900,Os08g0169700,Os07g0546700,Os07g0687500,Os06g0561501,Os01g0311500,Os08g0290000,Os11g0673000,	GO:0043405	regulation of MAP kinase activity	Any process that modulates the frequency, rate or extent of MAP kinase activity. [GOC:dph, GOC:go_curators]	0.004137322
215	Os04g0401900,Os01g0558500,Os11g0139900,Os03g0347800,Os06g0557600,Os07g0190900,Os06g0604500,Os09g0497100,Os11g0180100,Os05g0203912,XLOC_043736,Os04g0556100,XLOC_035920,	GO:0004045	aminoacyl-tRNA hydrolase activity	Catalysis of the reaction: N-substituted aminoacyl-tRNA + H2O = N-substituted amino acid + tRNA. [EC:3.1.1.29]	0.00437549
74	Os08g0282200,Os08g0444100,Os01g0128700,Os02g0522100,Os01g0136300,	GO:0015074	DNA integration	The process in which a segment of DNA is incorporated into another, usually larger, DNA molecule such as a chromosome. [GOC:mah]	0.004376013
46	Os01g0621600,Os10g0474800,Os05g0353500,Os03g0685300,Os02g0241200,Os05g0518300,Os03g0708400,	GO:0006541	glutamine metabolic process	The chemical reactions and pathways involving glutamine, 2-amino-4-carbamoylbutanoic acid. [GOC:ai]	0.004455045
217	Os03g0627500,Os03g0187600,Os03g0188200,	GO:0009911	positive regulation of flower development	Any process that activates or increases the frequency, rate or extent of flower development. [GOC:go_curators]	0.004536043
142	Os03g0818800,Os07g0235800,Os05g0334400,Os05g0163400,	GO:0006351	transcription, DNA-templated	The cellular synthesis of RNA on a template of DNA. [GOC:jl, GOC:txnOH]	0.004579463

78	Os05g0529700,Os07g0250300,Os06g0298400,Os01g0748600,Os09g0112100,<br>Os04g0415000,Os10g0456950,Os03g0570775,Os08g0564000,	GO:0009966	regulation of signal transduction	Any process that modulates the frequency, rate or extent of signal transduction. [GOC:sm]	0.004653374
224	Os10g0571100,Os12g0610600,Os04g0486800,	GO:0042631	cellular response to water deprivation	Any process that results in a change in state or activity of a cell (in terms of movement, secretion, enzyme production, gene expression, etc.) as a result of deprivation of water. [GOC:go_curators]	0.004655227
128	Os02g0654700,Os08g0177600,Os02g0123500,Os09g0359900,	GO:0006302	double-strand break repair	The repair of double-strand breaks in DNA via homologous and nonhomologous mechanisms to reform a continuous DNA helix. [GOC:elh]	0.004773546
230	Os05g0145000,Os07g0197400,Os01g0694200,	GO:0010413	glucuronoxylan metabolic process	The chemical reactions and pathways involving xylan, a polymer containing a beta-(1->4)-linked D-xylose backbone decorated with glucuronic acid side units. [GOC:tair_curators]	0.005012721
238	Os07g0568100,Os04g0491100,Os01g0956600,	GO:0010413	glucuronoxylan metabolic process	The chemical reactions and pathways involving xylan, a polymer containing a beta-(1->4)-linked D-xylose backbone decorated with glucuronic acid side units. [GOC:tair_curators]	0.005012721
253	Os09g0571100,Os12g0574700,Os07g0162900,	GO:0045330	aspartyl esterase activity	Catalysis of the hydrolysis of an ester bond by a mechanism involving a catalytically active aspartic acid residue. [GOC:mah, UniProtKB-KW:KW-0063]	0.005131867
82	Os12g0552700,Os12g0615500,Os04g0166000,Os03g0689688,Os05g0477500,	GO:0009407	toxin catabolic process	The chemical reactions and pathways resulting in the breakdown of toxin, a poisonous compound (typically a protein) that is produced by cells or organisms and that can cause disease when introduced into the body or tissues of an organism. [GOC:go_curators]	0.005368421
221	Os05g0169500,Os10g0437100,Os01g0767100,	GO:0015171	amino acid transmembrane transporter activity	Catalysis of the transfer of amino acids from one side of a membrane to the other. Amino acids are organic molecules that contain an amino group and a carboxyl group. [GOC:ai, GOC:mtg_transport, ISBN:0815340729]	0.005727454
136	Os08g0247600,Os03g0816000,Os02g0728500,Os06g0474866,	GO:0051607	defense response to virus	Reactions triggered in response to the presence of a virus that act to protect the cell or organism. [GOC:ai]	0.00588491
27	Os01g0200350,Os01g0646800,Os04g0610100,Os08g0112000,Os07g0164700,<br>Os01g0377700,Os01g0374900,Os04g0690451,Os03g0413250,	GO:0000723	telomere maintenance	Any process that contributes to the maintenance of proper telomeric length and structure by affecting and monitoring the activity of telomeric proteins and the length of telomeric DNA. These processes includes those that shorten and lengthen the telomeric DNA sequences. [GOC:elh, PMID:11092831]	0.006081304

273	Os01g0515300,Os04g0534200,Os06g0130100,	GO:0010103	stomatal complex morphogenesis	The process in which the anatomical structures of the stomatal complex are generated and organized. The stomatal complex is the stomatal guard cells and their associated epidermal cells. [GOC:tair_curators]	0.006084692
61	Os01g0174700,Os05g0592500,Os01g0956700,Os11g0702100,XLOC_031802, Os05g0548400,	GO:0004568	chitinase activity	Catalysis of the hydrolysis of (1->4)-beta linkages of N-acetyl-D-glucosamine (GlcNAc) polymers of chitin and chitodextrins. [EC:3.2.1.14, GOC:bf, GOC:kah, GOC:pde, PMID:11468293]	0.006161778
144	Os03g0752500,Os03g0782200,Os05g0512200,Os02g0798200,	GO:0010264	myo-inositol hexakisphosphate biosynthetic process	The chemical reactions and pathways resulting in the formation of phytic acid, myo-inositol hexakisphosphate, a regulator of intracellular signaling, a highly abundant animal anti-nutrient and a phosphate and mineral storage compound in plant seeds. [CHEBI:17401, PMID:16107538]	0.006202271
4	Os10g0363500,Os03g0179400,Os03g0832800,Os04g0301100,Os10g0204100, XLOC_044456,Os05g0589000,Os06g0140200,Os09g0511700,Os03g0125900, Os01g0179200,Os09g0397800,Os01g0148400,Os07g0111600,XLOC_030698, Os12g0162100,Os07g0273900,Os01g0777700,Os05g0545400,XLOC_077187, Os03g0362500,Os11g0535600,Os01g0214200,Os02g0202900,XLOC_057869, Os03g0755000,Os10g0515200,Os12g0234000,Os10g0324600,Os06g0111800, Os10g0136200,Os02g0148100,Os06g0700000,Os11g0148200,XLOC_039388, Os10g0400100,Os03g0229575,Os07g0101300,XLOC_058915,XLOC_024435, Os07g0541200,Os01g0704100,Os12g0115300,Os02g0511500,Os05g0531000, Os08g0220600,Os01g0894500,Os06g0714800,Os11g0116000,Os10g0151800, Os12g0570075,Os12g0285100,XLOC_033881,Os09g0271000,Os04g0432250, Os02g0564700,Os08g0472600,XLOC_059517,Os03g0827500,Os11g0571900, [illegible]	GO:0006869	lipid transport	The directed movement of lipids into, out of or within a cell, or between cells, by means of some agent such as a transporter or pore. Lipids are compounds soluble in an organic solvent but not, or sparingly, in an aqueous solvent. [ISBN:0198506732]	0.006299755
207	Os03g0255200,Os07g0189800,Os02g0671100,	GO:0009073	aromatic amino acid family biosynthetic process	The chemical reactions and pathways resulting in the formation of aromatic amino acid family, amino acids with aromatic ring (phenylalanine, tyrosine, tryptophan). [GOC:go_curators]	0.006322803
84	Os07g0498800,XLOC_029671,Os08g0296700,Os01g0120700,Os09g0509500,	GO:0000956	nuclear-transcribed mRNA catabolic process	The chemical reactions and pathways resulting in the breakdown of nuclear-transcribed mRNAs in eukaryotic cells. [GOC:krc]	0.006360924
174	Os10g0498800,Os04g0692400,Os05g0170000,Os09g0240975,	GO:0008375	acetylglucosaminyltransferase activity	Catalysis of the transfer of an N-acetylglucosaminyl residue from UDP-N-acetyl-glucosamine to a sugar. [ISBN:0198506732]	0.006678171
282	Os01g0524500,Os10g0181600,Os08g0459700,	GO:0030145	manganese ion binding	Interacting selectively and non-covalently with manganese (Mn) ions. [GOC:ai]	0.006798911
274	Os06g0230801,Os12g0283100,Os07g0203300,	GO:0001510	RNA methylation	Posttranscriptional addition of a methyl group to either a nucleotide or 2'-O ribose in a polyribonucleotide. Usually uses S-adenosylmethionine as a cofactor. [GOC:hjd]	0.007036908
286	Os01g0840200,Os05g0559400,Os04g0619800,	GO:0042538	hyperosmotic salinity response	Any process that results in a change in state or activity of a cell or an organism (in terms of movement, secretion, enzyme production, gene expression, etc.) as a result of detection of, or exposure to, an increase in the concentration of salt (particularly but not exclusively sodium and chloride ions) in the environment. [GOC:jl]	0.007274867

283	Os06g0472400,Os07g0496500,Os01g0590700,	GO:0004222	metalloendopeptidase activity	Catalysis of the hydrolysis of internal, alpha-peptide bonds in a polypeptide chain by a mechanism in which water acts as a nucleophile, one or two metal ions hold the water molecule in place, and charged amino acid side chains are ligands for the metal ions. [GOC:mah, http://merops.sanger.ac.uk/about/glossary.htm#CATTYPE, http://merops.sanger.ac.uk/about/glossary.htm#ENDOPEPTIDASE]	0.007631734
192	Os11g0608000,Os12g0527900,Os02g0494600,	GO:0009560	embryo sac egg cell differentiation	The process in which an uncellularized embryo sac nucleus cellularizes and acquires the specialized features of an egg cell. An example of this process is found in Arabidopsis thaliana. [GOC:jid, GOC:mtg_plant, GOC:mtg_sensu]	0.007988516
263	Os05g0574300,Os02g0820400,Os12g0528100,	GO:0000786	nucleosome	A complex comprised of DNA wound around a multisubunit core and associated proteins, which forms the primary packing unit of DNA into higher order structures. [GOC:elh]	0.007988516
168	Os07g0626800,Os05g0589600,Os07g0231800,Os04g0110500,	GO:0010103	stomatal complex morphogenesis	The process in which the anatomical structures of the stomatal complex are generated and organized. The stomatal complex is the stomatal guard cells and their associated epidermal cells. [GOC:tair_curators]	0.008104845
182	Os04g0531400,Os06g0138600,Os02g0767200,Os11g0661900,	GO:0004806	triglyceride lipase activity	Catalysis of the reaction: triacylglycerol + H2O = diacylglycerol + a carboxylate. [EC:3.1.1.3]	0.008263269
145	Os01g0179800,Os09g0481800,Os04g0556550,Os07g0619400,	GO:0009073	aromatic amino acid family biosynthetic process	The chemical reactions and pathways resulting in the formation of aromatic amino acid family, amino acids with aromatic ring (phenylalanine, tyrosine, tryptophan). [GOC:go_curators]	0.008421675
151	Os05g0564200,Os11g0544200,Os06g0528350,Os03g0728800,	GO:0008380	RNA splicing	The process of removing sections of the primary RNA transcript to remove sequences not present in the mature form of the RNA and joining the remaining sections to form the mature form of the RNA. [GOC:krc, GOC:mah]	0.008421675
299	Os09g0491820,Os03g0158000,Os01g0111000,	GO:0004857	enzyme inhibitor activity	Stops, prevents or reduces the activity of an enzyme. [GOC:ai]	0.008820674
185	Os03g0740200,Os10g0554800,Os08g0148300,Os04g0405500,Os01g0264400, Os05g0430900,Os01g0888300,	GO:0006857	oligopeptide transport	The directed movement of oligopeptides into, out of or within a cell, or between cells, by means of some agent such as a transporter or pore. Oligopeptides are molecules that contain a small number (2 to 20) of amino-acid residues connected by peptide linkages. [ISBN:0198506732]	0.009446625
229	Os04g0106500,Os10g0450000,Os10g0532000,	GO:0010200	response to chitin	A process that results in a change in state or activity of a cell or an organism (in terms of movement, secretion, enzyme production, gene expression, etc.) as a result of a chitin stimulus. [GOC:sm]	0.010246147

81	Os06g0290701,Os01g0102900,Os12g0257000,Os03g0602500,Os06g0698748,	GO:0004180	carboxypeptidase activity	Catalysis of the hydrolysis of the terminal or penultimate peptide bond at the C-terminal end of a peptide or polypeptide. [ISBN:0198506732]	0.010713762
63	Os03g0192900,Os05g0299300,Os04g0423700,Os12g0425500,Os11g0303400,Os06g0607000,	GO:0048316	seed development	The process whose specific outcome is the progression of the seed over time, from its formation to the mature structure. A seed is a propagating organ formed in the sexual reproductive cycle of gymnosperms and angiosperms, consisting of a protective coat enclosing an embryo and food reserves. [GOC:jid, PO:0009010]	0.011185923
56	Os07g0225100,Os12g0242800,Os07g0273600,Os12g0616800,Os08g0198000,Os01g0967800,	GO:0003333	amino acid transmembrane transport	The directed movement of amino acids, organic acids containing one or more amino substituents across a membrane by means of some agent such as a transporter or pore. [GOC:dph, GOC:tb]	0.012133082
266	Os01g0223900,Os08g0482100,Os06g0587900,	GO:0048544	recognition of pollen	The process, involving the sharing and interaction of the single locus incompatibility haplotypes, involved in the recognition or rejection of the self pollen by cells in the stigma. This process ensures out-breeding in certain plant species. [GOC:dph, GOC:pj, GOC:tb]	0.012263227
34	Os01g0665750,Os09g0535900,Os12g0534000,Os02g0258800,Os01g0101600,Os09g0454200,Os03g0277100,Os11g0123066,	GO:0008081	phosphoric diester hydrolase activity	Catalysis of the hydrolysis of a phosphodiester to give a phosphomonoester and a free hydroxyl group. [EC:3.1.4, GOC:curators]	0.012367055
107	Os03g0752800,Os06g0130800,Os02g0809100,Os02g0167600,Os05g0312201,	GO:0000786	nucleosome	A complex comprised of DNA wound around a multisubunit core and associated proteins, which forms the primary packing unit of DNA into higher order structures. [GOC:elh]	0.013279234
301	Os05g0551100,Os06g0695500,Os05g0534500,	GO:0030001	metal ion transport	The directed movement of metal ions, any metal ion with an electric charge, into, out of or within a cell, or between cells, by means of some agent such as a transporter or pore. [GOC:ai]	0.013329983
146	Os02g0527300,Os03g0752100,Os06g0622300,Os11g0678200,	GO:0010200	response to chitin	A process that results in a change in state or activity of a cell or an organism (in terms of movement, secretion, enzyme production, gene expression, etc.) as a result of a chitin stimulus. [GOC:sm]	0.013638417
70	Os05g0535100,Os09g0437500,Os01g0220300,Os04g0164300,Os04g0469700,	GO:0019843	rRNA binding	Interacting selectively and non-covalently with ribosomal RNA. [GOC:jl]	0.014264536
206	Os08g0174500,Os07g0195350,Os05g0172400,	GO:0046982	protein heterodimerization activity	Interacting selectively and non-covalently with a nonidentical protein to form a heterodimer. [GOC:ai]	0.014869496

277	Os02g0695600,Os04g0598900,Os07g0142900,	GO:0030247	polysaccharide binding	Interacting selectively and non-covalently with any polysaccharide, a polymer of many (typically more than 10) monosaccharide residues linked glycosidically. [CHEBI:18154, GOC:mah]	0.014987853
189	Os11g0594600,Os02g0807750,Os04g0677400,Os01g0863166,Os07g0556300, Os02g0506100,Os06g0329300,Os07g0176500,	GO:0048767	root hair elongation	The process in which the root hair grows longer. [GOC:jid, PMID:12468740]	0.015516455
51	Os02g0201900,Os02g0725300,Os06g0713300,Os01g0584800,Os02g0168900, Os11g0617800,Os11g0245800,	GO:0016760	cellulose synthase (UDP-forming) activity	Catalysis of the reaction: UDP-glucose + ((1,4)-beta-D-glucosyl)(n) = UDP + ((1,4)-beta-D-glucosyl)(n+1). [EC:2.4.1.12]	0.016068684
180	Os01g0223800,Os07g0186500,Os12g0565200,Os01g0223700,	GO:0048544	recognition of pollen	The process, involving the sharing and interaction of the single locus incompatibility haplotypes, involved in the recognition or rejection of the self pollen by cells in the stigma. This process ensures out-breeding in certain plant species. [GOC:dph, GOC:pj, GOC:tb]	0.016317782
120	Os04g0560100,Os06g0121500,Os07g0489200,Os05g0150733,	GO:0030001	metal ion transport	The directed movement of metal ions, any metal ion with an electric charge, into, out of or within a cell, or between cells, by means of some agent such as a transporter or pore. [GOC:ai]	0.017734058
102	Os03g0800800,XLOC_042345,Os09g0325220,XLOC_047572,XLOC_019266,	GO:0005515	protein binding	Interacting selectively and non-covalently with any protein or protein complex (a complex of two or more proteins that may include other nonprotein molecules). [GOC:go_curators]	0.019447102
248	Os02g0790600,Os12g0158900,Os01g0212900,	GO:0080167	response to karrikin	Any process that results in a change in state or activity of a cell or an organism (in terms of movement, secretion, enzyme production, gene expression, etc.) as a result of a karrikin stimulus. Karrikins are signaling molecules in smoke from burning vegetation that trigger seed germination for many angiosperms (flowering plants). [PMID:20351290]	0.020186212
223	Os02g0321800,Os04g0578700,Os11g0251400,	GO:0006886	intracellular protein transport	The directed movement of proteins in a cell, including the movement of proteins between specific compartments or structures within a cell, such as organelles of a eukaryotic cell. [GOC:mah]	0.021482937
216	Os01g0953400,Os11g0703900,Os04g0483000,	GO:0009408	response to heat	Any process that results in a change in state or activity of a cell or an organism (in terms of movement, secretion, enzyme production, gene expression, etc.) as a result of a heat stimulus, a temperature stimulus above the optimal temperature for that organism. [GOC:lr]	0.025601269
187	Os09g0284300,Os09g0360900,Os01g0924800,Os05g0171900,Os05g0214232, Os03g0260600,	GO:0022625	cytosolic large ribosomal subunit	The large subunit of a ribosome located in the cytosol. [GOC:mtg_sensu]	0.026951402

68	Os03g0615300,Os05g0496000,Os08g0379000,Os02g0117550,Os03g0101400, Os01g0318600,	GO:0006396	RNA processing	Any process involved in the conversion of one or more primary RNA transcripts into one or more mature RNA molecules. [GOC:mah]	0.031616708
91	Os04g0487000,Os03g0608800,Os01g0668600,Os07g0195100,Os10g0163300, Os05g0432400,Os03g0794900,XLOC_070320,Os12g0535300,	GO:0007264	small GTPase mediated signal transduction	Any series of molecular signals in which a small monomeric GTPase relays one or more of the signals. [GOC:mah]	0.031752097
212	Os03g0718800,Os12g0169950,Os07g0693700,	GO:0032259	methylation	The process in which a methyl group is covalently attached to a molecule. [GOC:mah]	0.031815578
122	Os03g0219800,Os03g0262500,Os11g0146950,Os06g0472700,Os06g0181400, Os11g0689400,Os07g0534100,XLOC_027908,	GO:0008026	ATP-dependent helicase activity	Catalysis of the reaction: ATP + H2O = ADP + phosphate, to drive the unwinding of a DNA or RNA helix. [EC:3.6.1.3, GOC:jl]	0.033271348
288	Os12g0143100,XLOC_047650,Os03g0744675,	GO:0005488	binding	The selective, non-covalent, often stoichiometric, interaction of a molecule with one or more specific sites on another molecule. [GOC:ceb, GOC:mah, ISBN:0198506732]	0.033271715
35	Os04g0430700,Os01g0658900,Os06g0640599,Os02g0272350,Os06g0639800, Os04g0555800,Os08g0158500,Os01g0107400,Os07g0565700,	GO:0008236	serine-type peptidase activity	Catalysis of the hydrolysis of peptide bonds in a polypeptide chain by a catalytic mechanism that involves a catalytic triad consisting of a serine nucleophile that is activated by a proton relay involving an acidic residue (e.g. aspartate or glutamate) and a basic residue (usually histidine). [http://merops.sanger.ac.uk/about/glossary.htm#CATTYPE, ISBN:0716720094]	0.033564421
247	Os08g0388300,Os09g0279100,Os06g0698822,	GO:0005524	ATP binding	Interacting selectively and non-covalently with ATP, adenosine 5'-triphosphate, a universally important coenzyme and enzyme regulator. [ISBN:0198506732]	0.038421458
17	XLOC_060303,XLOC_060304,XLOC_040714,XLOC_040716,XLOC_060305, XLOC_076740,XLOC_023258,XLOC_077209,XLOC_060301,XLOC_060300, XLOC_060298,Os01g0765300,	GO:0003676	nucleic acid binding	Interacting selectively and non-covalently with any nucleic acid. [GOC:jl]	0.042080096
104	Os05g0481800,Os07g0636900,Os12g0270300,XLOC_073764,Os01g0207200,	GO:0009505	plant-type cell wall	A more or less rigid stucture lying outside the cell membrane of a cell and composed of cellulose and pectin and other organic and inorganic substances. [ISBN:0471245208]	0.043120704
88	Os04g0110200,Os05g0564100,Os01g0602600,Os02g0599100,Os12g0127200, Os05g0298900,	GO:0016747	transferase activity, transferring acyl groups other than amino-acyl groups	Catalysis of the transfer of an acyl group, other than amino-acyl, from one compound (donor) to another (acceptor). [GOC:jl]	0.045730926

214	Os06g0490200,Os02g0803400,Os12g0596600,	GO:0005215	transporter activity	Enables the directed movement of substances (such as macromolecules, small molecules, ions) into, out of or within a cell, or between cells. [GOC:ai, GOC:dgf]	0.047061989
225	Os10g0494950,Os11g0158500,Os08g0521000,	GO:0046983	protein dimerization activity	The formation of a protein dimer, a macromolecular structure consists of two noncovalently associated identical or nonidentical subunits. [ISBN:0198506732]	0.048103536
264	Os01g0726400,Os09g0412900,Os07g0590600,	GO:0046983	protein dimerization activity	The formation of a protein dimer, a macromolecular structure consists of two noncovalently associated identical or nonidentical subunits. [ISBN:0198506732]	0.048103536
303	Os01g0327400,Os05g0439750,Os01g0626900,Os02g0658500,Os02g0125200, Os10g0141500,	GO:0004601	peroxidase activity	Catalysis of the reaction: donor + hydrogen peroxide = oxidized donor + 2 H2O. [EC:1.11.1.7]	0.049175247
209	Os07g0628900,Os01g0584300,Os12g0493900,	GO:0005488	binding	The selective, non-covalent, often stoichiometric, interaction of a molecule with one or more specific sites on another molecule. [GOC:ceb, GOC:mah, ISBN:0198506732]	0.049491084
157	Os04g0620950,Os07g0645200,Os08g0508700,Os11g0486000,	GO:0043531	ADP binding	Interacting selectively and non-covalently with ADP, adenosine 5'-diphosphate. [GOC:jl]	0.065893426
152	Os12g0218500,Os12g0222800,Os03g0829100,Os04g0338000,	GO:0046686	response to cadmium ion	Any process that results in a change in state or activity of a cell or an organism (in terms of movement, secretion, enzyme production, gene expression, etc.) as a result of a cadmium (Cd) ion stimulus. [GOC:ai]	0.078101343
269	Os01g0636400,Os09g0570200,XLOC_077108,	GO:0003676	nucleic acid binding	Interacting selectively and non-covalently with any nucleic acid. [GOC:jl]	0.082391063
172	Os03g0104400,Os05g0552800,Os09g0439200,Os01g0570500,	GO:0009506	plasmodesma	A fine cytoplasmic channel, found in all higher plants, that connects the cytoplasm of one cell to that of an adjacent cell. [ISBN:0198506732]	0.09404394
226	Os11g0599466,Os11g0114700,XLOC_009088,	GO:0016740	transferase activity	Catalysis of the transfer of a group, e.g. a methyl group, glycosyl group, acyl group, phosphorus-containing, or other groups, from one compound (generally regarded as the donor) to another compound (generally regarded as the acceptor). Transferase is the systematic name for any enzyme of EC class 2. [ISBN:0198506732]	0.116649455

228	Os02g0831200,Os03g0628800,Os03g0169900,	GO:0016310	phosphorylation	The process of introducing a phosphate group into a molecule, usually with the formation of a phosphoric ester, a phosphoric anhydride or a phosphoric amide. [ISBN:0198506732]	0.134105654
138	Os10g0577000,Os12g0556300,Os04g0663200,Os07g0277600,	GO:0005783	endoplasmic reticulum	The irregular network of unit membranes, visible only by electron microscopy, that occurs in the cytoplasm of many eukaryotic cells. The membranes form a complex meshwork of tubular channels, which are often expanded into slitlike cavities called cisternae. The ER takes two forms, rough (or granular), with ribosomes adhering to the outer surface, and smooth (with no ribosomes attached). [ISBN:0198506732]	0.144326145
210	Os02g0694400,Os12g0620000,Os07g0592300,	GO:0004674	protein serine/threonine kinase activity	Catalysis of the reactions: ATP + protein serine = ADP + protein serine phosphate, and ATP + protein threonine = ADP + protein threonine phosphate. [GOC:bf]	0.145674786
246	Os02g0518400,Os02g0686700,Os08g0247700,	GO:0004674	protein serine/threonine kinase activity	Catalysis of the reactions: ATP + protein serine = ADP + protein serine phosphate, and ATP + protein threonine = ADP + protein threonine phosphate. [GOC:bf]	0.145674786
203	Os04g0221600,Os04g0298600,Os01g0924200,	GO:0008270	zinc ion binding	Interacting selectively and non-covalently with zinc (Zn) ions. [GOC:ai]	0.164267321
297	Os05g0141500,Os07g0482566,Os03g0299800,	GO:0008270	zinc ion binding	Interacting selectively and non-covalently with zinc (Zn) ions. [GOC:ai]	0.164267321
254	Os03g0355900,Os03g0843200,Os09g0250700,	GO:0016772	transferase activity, transferring phosphorus-containing groups	Catalysis of the transfer of a phosphorus-containing group from one compound (donor) to another (acceptor). [GOC:jl, ISBN:0198506732]	0.188946983
270	Os05g0485300,Os10g0495200,Os10g0499500,	GO:0016021	integral component of membrane	The component of a membrane consisting of gene products and protein complexes that have some part that penetrates at least one leaflet of the membrane bilayer. This component includes gene products that are buried in the bilayer with no exposure outside the bilayer. [GOC:dos, GOC:go_curators]	0.222473056
232	Os05g0477800,Os04g0691800,Os05g0406550,	GO:0009507	chloroplast	A chlorophyll-containing plastid with thylakoids organized into grana and frets, or stroma thylakoids, and embedded in a stroma. [ISBN:0471245208]	0.222574138
191	Os08g0120301,Os05g0490000,Os06g0317100,	GO:0005829	cytosol	The part of the cytoplasm that does not contain organelles but which does contain other particulate matter, such as protein complexes. [GOC:hgd, GOC:jl]	0.24687837

163	Os06g0647500,Os04g0609900,Os01g0266000,Os02g0461100,XLOC_075932, Os06g0232400,	GO:0005829	cytosol	The part of the cytoplasm that does not contain organelles but which does contain other particulate matter, such as protein complexes. [GOC:hgd, GOC:jl]	0.37660025
249	Os12g0106300,Os01g0823500,Os03g0796700,	GO:0003677	DNA binding	Any molecular function by which a gene product interacts selectively and non-covalently with DNA (deoxyribonucleic acid). [GOC:dph, GOC:jl, GOC:tb, GOC:vw]	0.392836287
190	Os05g0232700,Os03g0572250,Os09g0290900,Os09g0356100,Os10g0126700,	GO:0005515	protein binding	Interacting selectively and non-covalently with any protein or protein complex (a complex of two or more proteins that may include other nonprotein molecules). [GOC:go_curators]	0.52815214
233	Os05g0182000,Os03g0441400,Os04g0500200,	GO:0008150	biological_process	Any process specifically pertinent to the functioning of integrated living units: cells, tissues, organs, and organisms. A process is a collection of molecular events with a defined beginning and end. [GOC:go_curators, GOC:isa_complete]	0.860864442
169	Os09g0488800,Os05g0149500,Os10g0457400,Os08g0397800,	GO:0008150	biological_process	Any process specifically pertinent to the functioning of integrated living units: cells, tissues, organs, and organisms. A process is a collection of molecular events with a defined beginning and end. [GOC:go_curators, GOC:isa_complete]	0.996980029
106	Os08g0394000,Os10g0485400,Os03g0575500,Os07g0187001,Os05g0420000,	GO:0000002	mitochondrial genome maintenance	The maintenance of the structure and integrity of the mitochondrial genome; includes replication and segregation of the mitochondrial chromosome. [GOC:ai, GOC:vw]	1
158	Os01g0836900,Os10g0389500,Os02g0582900,Os08g0428400,	GO:0000002	mitochondrial genome maintenance	The maintenance of the structure and integrity of the mitochondrial genome; includes replication and segregation of the mitochondrial chromosome. [GOC:ai, GOC:vw]	1
173	XLOC_073610,XLOC_064693,XLOC_020422,Os05g0556201,	GO:0000002	mitochondrial genome maintenance	The maintenance of the structure and integrity of the mitochondrial genome; includes replication and segregation of the mitochondrial chromosome. [GOC:ai, GOC:vw]	1
184	XLOC_026666,XLOC_056246,XLOC_072175,XLOC_012400,	GO:0000002	mitochondrial genome maintenance	The maintenance of the structure and integrity of the mitochondrial genome; includes replication and segregation of the mitochondrial chromosome. [GOC:ai, GOC:vw]	1
201	XLOC_072903,Os07g0564000,XLOC_071318,	GO:0000002	mitochondrial genome maintenance	The maintenance of the structure and integrity of the mitochondrial genome; includes replication and segregation of the mitochondrial chromosome. [GOC:ai, GOC:vw]	1

222	XLOC_009148,Os07g0543800,XLOC_035101,	GO:0000002	mitochondrial genome maintenance	The maintenance of the structure and integrity of the mitochondrial genome; includes replication and segregation of the mitochondrial chromosome. [GOC:ai, GOC:vw]	1
235	Os05g0515700,Os01g0225100,Os05g0417700,	GO:0000002	mitochondrial genome maintenance	The maintenance of the structure and integrity of the mitochondrial genome; includes replication and segregation of the mitochondrial chromosome. [GOC:ai, GOC:vw]	1
240	Os04g0480500,Os12g0212400,Os12g0626000,	GO:0000002	mitochondrial genome maintenance	The maintenance of the structure and integrity of the mitochondrial genome; includes replication and segregation of the mitochondrial chromosome. [GOC:ai, GOC:vw]	1
241	Os06g0124300,Os04g0163800,Os06g0122500,	GO:0000002	mitochondrial genome maintenance	The maintenance of the structure and integrity of the mitochondrial genome; includes replication and segregation of the mitochondrial chromosome. [GOC:ai, GOC:vw]	1
242	Os04g0684100,Os03g0559700,Os05g0150550,	GO:0000002	mitochondrial genome maintenance	The maintenance of the structure and integrity of the mitochondrial genome; includes replication and segregation of the mitochondrial chromosome. [GOC:ai, GOC:vw]	1
265	XLOC_008789,Os07g0575900,Os01g0834250,	GO:0000002	mitochondrial genome maintenance	The maintenance of the structure and integrity of the mitochondrial genome; includes replication and segregation of the mitochondrial chromosome. [GOC:ai, GOC:vw]	1
279	Os01g0553600,Os11g0641300,Os12g0121200,	GO:0000002	mitochondrial genome maintenance	The maintenance of the structure and integrity of the mitochondrial genome; includes replication and segregation of the mitochondrial chromosome. [GOC:ai, GOC:vw]	1

Table S3. Annotations of the LincRNAs in Rice Root

LincRNA	GO ID	GO name	GO description
XLOC_049097	GO:0000124	SAGA complex	A SAGA-type histone acetyltransferase complex that contains Spt8 (in budding yeast) or a homolog thereof; additional polypeptides include Spt group, consisting of Spt7, Spt3, and Spt20/Ada5, which interact with the TATA-binding protein (TBP); the Ada group, consisting of Ada1, Ada2, Ada3, Ada4/Gcn5, and Ada5/Spt20, which is functionally linked to the nucleosomal HAT activity; Tra1, an ATM/PI-3 kinase-related protein that targets DNA-bound activators for recruitment to promoters; the TBP-associated factor (TAF) proteins, consisting of Taf5, Taf6, Taf9, Taf10, and Taf12, which mediate nucleosomal HAT activity and are thought to help recruit the basal transcription machinery. [PMID:10637607, PMID:17337012]
XLOC_032300	GO:0000404	loop DNA binding	Interacting selectively and non-covalently with DNA containing a loop. A loop occurs when DNA contains a large insertion or deletion that causes a region of unpaired single-stranded DNA to loop out, while the rest of the DNA is in a paired double-stranded configuration. [GOC:elh, PMID:16781730]
XLOC_068568	GO:0000723	telomere maintenance	Any process that contributes to the maintenance of proper telomeric length and structure by affecting and monitoring the activity of telomeric proteins and the length of telomeric DNA. These processes includes those that shorten and lengthen the telomeric DNA sequences. [GOC:elh, PMID:11092831]
XLOC_076924	GO:0000723	telomere maintenance	Any process that contributes to the maintenance of proper telomeric length and structure by affecting and monitoring the activity of telomeric proteins and the length of telomeric DNA. These processes includes those that shorten and lengthen the telomeric DNA sequences. [GOC:elh, PMID:11092831]

XLOC_001099	GO:0002237	response to molecule of bacterial origin	Any process that results in a change in state or activity of an organism (in terms of movement, secretion, enzyme production, gene expression, etc.) as a result of a stimulus by molecules of bacterial origin such as peptides derived from bacterial flagellin. [GOC:rl, GOC:sm]
XLOC_006498	GO:0002237	response to molecule of bacterial origin	Any process that results in a change in state or activity of an organism (in terms of movement, secretion, enzyme production, gene expression, etc.) as a result of a stimulus by molecules of bacterial origin such as peptides derived from bacterial flagellin. [GOC:rl, GOC:sm]
XLOC_008468	GO:0002237	response to molecule of bacterial origin	Any process that results in a change in state or activity of an organism (in terms of movement, secretion, enzyme production, gene expression, etc.) as a result of a stimulus by molecules of bacterial origin such as peptides derived from bacterial flagellin. [GOC:rl, GOC:sm]
XLOC_013623	GO:0002237	response to molecule of bacterial origin	Any process that results in a change in state or activity of an organism (in terms of movement, secretion, enzyme production, gene expression, etc.) as a result of a stimulus by molecules of bacterial origin such as peptides derived from bacterial flagellin. [GOC:rl, GOC:sm]

XLOC_059443	GO:0002237	response to molecule of bacterial origin	Any process that results in a change in state or activity of an organism (in terms of movement, secretion, enzyme production, gene expression, etc.) as a result of a stimulus by molecules of bacterial origin such as peptides derived from bacterial flagellin. [GOC:rl, GOC:sm]
XLOC_059896	GO:0002237	response to molecule of bacterial origin	Any process that results in a change in state or activity of an organism (in terms of movement, secretion, enzyme production, gene expression, etc.) as a result of a stimulus by molecules of bacterial origin such as peptides derived from bacterial flagellin. [GOC:rl, GOC:sm]
XLOC_016325	GO:0003860	3-hydroxyisobutyryl-CoA hydrolase activity	Catalysis of the reaction: 3-hydroxy-2-methylpropanoyl-CoA + H2O = CoA + 3-hydroxy-2-methylpropanoate. [EC:3.1.2.4]
XLOC_040881	GO:0004151	dihydroorotase activity	Catalysis of the reaction: (S)-dihydroorotate + H(2)O = N-carbamoyl-L-aspartate + H(+). [EC:3.5.2.3, RHEA:24299]
XLOC_058130	GO:0004351	glutamate decarboxylase activity	Catalysis of the reaction: L-glutamate = 4-aminobutanoate + CO2. [EC:4.1.1.15]
XLOC_017209	GO:0004607	phosphatidylcholine-sterol O-acyltransferase activity	Catalysis of the reaction: phosphatidylcholine + a sterol = a sterol ester + 1-acylglycerophosphocholine. [EC:2.3.1.43]
XLOC_054029	GO:0004607	phosphatidylcholine-sterol O-acyltransferase activity	Catalysis of the reaction: phosphatidylcholine + a sterol = a sterol ester + 1-acylglycerophosphocholine. [EC:2.3.1.43]
XLOC_066660	GO:0004629	phospholipase C activity	Catalysis of the reaction: a phospholipid + H2O = 1,2-diacylglycerol + a phosphatidate. [EC:3.1.4.3, EC:3.1.4.4, GOC:mah]
XLOC_015851	GO:0004823	leucine-tRNA ligase activity	Catalysis of the reaction: L-leucine + ATP + tRNA(Leu) = AMP + diphosphate + 2 H(+) + Leu-tRNA(Leu). [EC:6.1.1.4, RHEA:11691]

XLOC_031802	GO:0005488	binding	The selective, non-covalent, often stoichiometric, interaction of a molecule with one or more specific sites on another molecule. [GOC:ceb, GOC:mah, ISBN:0198506732]
XLOC_045126	GO:0005509	calcium ion binding	Interacting selectively and non-covalently with calcium ions (Ca2+). [GOC:ai]
XLOC_001874	GO:0005774	vacuolar membrane	The lipid bilayer surrounding the vacuole and separating its contents from the cytoplasm of the cell. [GOC:ai]
XLOC_010893	GO:0005774	vacuolar membrane	The lipid bilayer surrounding the vacuole and separating its contents from the cytoplasm of the cell. [GOC:ai]
XLOC_015818	GO:0005774	vacuolar membrane	The lipid bilayer surrounding the vacuole and separating its contents from the cytoplasm of the cell. [GOC:ai]
XLOC_067697	GO:0005774	vacuolar membrane	The lipid bilayer surrounding the vacuole and separating its contents from the cytoplasm of the cell. [GOC:ai]
XLOC_073131	GO:0005774	vacuolar membrane	The lipid bilayer surrounding the vacuole and separating its contents from the cytoplasm of the cell. [GOC:ai]
XLOC_074955	GO:0005774	vacuolar membrane	The lipid bilayer surrounding the vacuole and separating its contents from the cytoplasm of the cell. [GOC:ai]
XLOC_059805	GO:0005829	cytosol	The part of the cytoplasm that does not contain organelles but which does contain other particulate matter, such as protein complexes. [GOC:hgd, GOC:jl]
XLOC_008525	GO:0006073	cellular glucan metabolic process	The chemical reactions and pathways involving glucans, polysaccharides consisting only of glucose residues. [ISBN:0198547684]
XLOC_009492	GO:0006848	pyruvate transport	The directed movement of pyruvate into, out of or within a cell, or between cells, by means of some agent such as a transporter or pore. [GOC:krc]
XLOC_020260	GO:0006848	pyruvate transport	The directed movement of pyruvate into, out of or within a cell, or between cells, by means of some agent such as a transporter or pore. [GOC:krc]

XLOC_025834	GO:0006848	pyruvate transport	The directed movement of pyruvate into, out of or within a cell, or between cells, by means of some agent such as a transporter or pore. [GOC:krc]
XLOC_026516	GO:0006848	pyruvate transport	The directed movement of pyruvate into, out of or within a cell, or between cells, by means of some agent such as a transporter or pore. [GOC:krc]
XLOC_030698	GO:0006848	pyruvate transport	The directed movement of pyruvate into, out of or within a cell, or between cells, by means of some agent such as a transporter or pore. [GOC:krc]
XLOC_032273	GO:0006848	pyruvate transport	The directed movement of pyruvate into, out of or within a cell, or between cells, by means of some agent such as a transporter or pore. [GOC:krc]
XLOC_032724	GO:0006848	pyruvate transport	The directed movement of pyruvate into, out of or within a cell, or between cells, by means of some agent such as a transporter or pore. [GOC:krc]
XLOC_040629	GO:0006848	pyruvate transport	The directed movement of pyruvate into, out of or within a cell, or between cells, by means of some agent such as a transporter or pore. [GOC:krc]
XLOC_040981	GO:0006848	pyruvate transport	The directed movement of pyruvate into, out of or within a cell, or between cells, by means of some agent such as a transporter or pore. [GOC:krc]
XLOC_044441	GO:0006848	pyruvate transport	The directed movement of pyruvate into, out of or within a cell, or between cells, by means of some agent such as a transporter or pore. [GOC:krc]
XLOC_044634	GO:0006848	pyruvate transport	The directed movement of pyruvate into, out of or within a cell, or between cells, by means of some agent such as a transporter or pore. [GOC:krc]
XLOC_051599	GO:0006848	pyruvate transport	The directed movement of pyruvate into, out of or within a cell, or between cells, by means of some agent such as a transporter or pore. [GOC:krc]
XLOC_055176	GO:0006848	pyruvate transport	The directed movement of pyruvate into, out of or within a cell, or between cells, by means of some agent such as a transporter or pore. [GOC:krc]
XLOC_060222	GO:0006848	pyruvate transport	The directed movement of pyruvate into, out of or within a cell, or between cells, by means of some agent such as a transporter or pore. [GOC:krc]

XLOC_067770	GO:0006848	pyruvate transport	The directed movement of pyruvate into, out of or within a cell, or between cells, by means of some agent such as a transporter or pore. [GOC:krc]
XLOC_077187	GO:0006848	pyruvate transport	The directed movement of pyruvate into, out of or within a cell, or between cells, by means of some agent such as a transporter or pore. [GOC:krc]
XLOC_044650	GO:0006952	defense response	Reactions, triggered in response to the presence of a foreign body or the occurrence of an injury, which result in restriction of damage to the organism attacked or prevention/recovery from the infection caused by the attack. [GOC:go_curators]
XLOC_045368	GO:0006952	defense response	Reactions, triggered in response to the presence of a foreign body or the occurrence of an injury, which result in restriction of damage to the organism attacked or prevention/recovery from the infection caused by the attack. [GOC:go_curators]
XLOC_040280	GO:0008289	lipid binding	Interacting selectively and non-covalently with a lipid. [GOC:ai]
XLOC_071610	GO:0008289	lipid binding	Interacting selectively and non-covalently with a lipid. [GOC:ai]
XLOC_066757	GO:0008430	selenium binding	Interacting selectively and non-covalently with selenium (Se). [GOC:ai]
XLOC_020078	GO:0008544	epidermis development	The process whose specific outcome is the progression of the epidermis over time, from its formation to the mature structure. The epidermis is the outer epithelial layer of a plant or animal, it may be a single layer that produces an extracellular material (e.g. the cuticle of arthropods) or a complex stratified squamous epithelium, as in the case of many vertebrate species. [GOC:go_curators, UBERON:0001003]
XLOC_037259	GO:0008544	epidermis development	The process whose specific outcome is the progression of the epidermis over time, from its formation to the mature structure. The epidermis is the outer epithelial layer of a plant or animal, it may be a single layer that produces an extracellular material (e.g. the cuticle of arthropods) or a complex stratified squamous epithelium, as in the case of many vertebrate species. [GOC:go_curators, UBERON:0001003]
XLOC_040499	GO:0008544	epidermis development	The process whose specific outcome is the progression of the epidermis over time, from its formation to the mature structure. The epidermis is the outer epithelial layer of a plant or animal, it may be a single layer that produces an extracellular material (e.g. the cuticle of arthropods) or a complex stratified squamous epithelium, as in the case of many vertebrate species. [GOC:go_curators, UBERON:0001003]

XLOC_008741	GO:0009250	glucan biosynthetic process	The chemical reactions and pathways resulting in the formation of glucans, polysaccharides consisting only of glucose residues. [GOC:go_curators]
XLOC_019493	GO:0009250	glucan biosynthetic process	The chemical reactions and pathways resulting in the formation of glucans, polysaccharides consisting only of glucose residues. [GOC:go_curators]
XLOC_024889	GO:0009250	glucan biosynthetic process	The chemical reactions and pathways resulting in the formation of glucans, polysaccharides consisting only of glucose residues. [GOC:go_curators]
XLOC_027868	GO:0009250	glucan biosynthetic process	The chemical reactions and pathways resulting in the formation of glucans, polysaccharides consisting only of glucose residues. [GOC:go_curators]
XLOC_031943	GO:0009250	glucan biosynthetic process	The chemical reactions and pathways resulting in the formation of glucans, polysaccharides consisting only of glucose residues. [GOC:go_curators]
XLOC_033910	GO:0009250	glucan biosynthetic process	The chemical reactions and pathways resulting in the formation of glucans, polysaccharides consisting only of glucose residues. [GOC:go_curators]
XLOC_044456	GO:0009250	glucan biosynthetic process	The chemical reactions and pathways resulting in the formation of glucans, polysaccharides consisting only of glucose residues. [GOC:go_curators]
XLOC_049577	GO:0009250	glucan biosynthetic process	The chemical reactions and pathways resulting in the formation of glucans, polysaccharides consisting only of glucose residues. [GOC:go_curators]
XLOC_053506	GO:0009250	glucan biosynthetic process	The chemical reactions and pathways resulting in the formation of glucans, polysaccharides consisting only of glucose residues. [GOC:go_curators]
XLOC_055397	GO:0009250	glucan biosynthetic process	The chemical reactions and pathways resulting in the formation of glucans, polysaccharides consisting only of glucose residues. [GOC:go_curators]
XLOC_058915	GO:0009250	glucan biosynthetic process	The chemical reactions and pathways resulting in the formation of glucans, polysaccharides consisting only of glucose residues. [GOC:go_curators]
XLOC_061152	GO:0009250	glucan biosynthetic process	The chemical reactions and pathways resulting in the formation of glucans, polysaccharides consisting only of glucose residues. [GOC:go_curators]

XLOC_062736	GO:0009250	glucan biosynthetic process	The chemical reactions and pathways resulting in the formation of glucans, polysaccharides consisting only of glucose residues. [GOC:go_curators]
XLOC_077203	GO:0009250	glucan biosynthetic process	The chemical reactions and pathways resulting in the formation of glucans, polysaccharides consisting only of glucose residues. [GOC:go_curators]
XLOC_035144	GO:0009941	chloroplast envelope	The double lipid bilayer enclosing the chloroplast and separating its contents from the rest of the cytoplasm; includes the intermembrane space. [GOC:tb]
XLOC_040426	GO:0009941	chloroplast envelope	The double lipid bilayer enclosing the chloroplast and separating its contents from the rest of the cytoplasm; includes the intermembrane space. [GOC:tb]
XLOC_036022	GO:0009982	pseudouridine synthase activity	Catalysis of the reaction: RNA uridine = RNA pseudouridine. Conversion of uridine in an RNA molecule to pseudouridine by rotation of the C1'-N-1 glycosidic bond of uridine in RNA to a C1'-C5. [EC:5.4.99.12, GOC:mah]
XLOC_034785	GO:0010037	response to carbon dioxide	Any process that results in a change in state or activity of a cell or an organism (in terms of movement, secretion, enzyme production, gene expression, etc.) as a result of a carbon dioxide (CO_2) stimulus. [GOC:sm]
XLOC_056467	GO:0010037	response to carbon dioxide	Any process that results in a change in state or activity of a cell or an organism (in terms of movement, secretion, enzyme production, gene expression, etc.) as a result of a carbon dioxide (CO_2) stimulus. [GOC:sm]
XLOC_009323	GO:0016036	cellular response to phosphate starvation	Any process that results in a change in state or activity of a cell (in terms of movement, secretion, enzyme production, gene expression, etc.) as a result of deprivation of phosphate. [GOC:jl]
XLOC_010233	GO:0016036	cellular response to phosphate starvation	Any process that results in a change in state or activity of a cell (in terms of movement, secretion, enzyme production, gene expression, etc.) as a result of deprivation of phosphate. [GOC:jl]
XLOC_026030	GO:0016036	cellular response to phosphate starvation	Any process that results in a change in state or activity of a cell (in terms of movement, secretion, enzyme production, gene expression, etc.) as a result of deprivation of phosphate. [GOC:jl]
XLOC_026206	GO:0016036	cellular response to phosphate starvation	Any process that results in a change in state or activity of a cell (in terms of movement, secretion, enzyme production, gene expression, etc.) as a result of deprivation of phosphate. [GOC:jl]
XLOC_036449	GO:0016036	cellular response to phosphate starvation	Any process that results in a change in state or activity of a cell (in terms of movement, secretion, enzyme production, gene expression, etc.) as a result of deprivation of phosphate. [GOC:jl]

XLOC_051315	GO:0016036	cellular response to phosphate starvation	Any process that results in a change in state or activity of a cell (in terms of movement, secretion, enzyme production, gene expression, etc.) as a result of deprivation of phosphate. [GOC:jl]
XLOC_054628	GO:0016036	cellular response to phosphate starvation	Any process that results in a change in state or activity of a cell (in terms of movement, secretion, enzyme production, gene expression, etc.) as a result of deprivation of phosphate. [GOC:jl]
XLOC_036169	GO:0016310	phosphorylation	The process of introducing a phosphate group into a molecule, usually with the formation of a phosphoric ester, a phosphoric anhydride or a phosphoric amide. [ISBN:0198506732]
XLOC_040618	GO:0016310	phosphorylation	The process of introducing a phosphate group into a molecule, usually with the formation of a phosphoric ester, a phosphoric anhydride or a phosphoric amide. [ISBN:0198506732]
XLOC_071807	GO:0016602	CCAAT-binding factor complex	A heteromeric transcription factor complex that binds to the CCAAT-box upstream of promoters; in Saccharomyces it activates the transcription of genes in response to growth in a nonfermentable carbon source; consists of four known subunits: HAP2, HAP3, HAP4 and HAP5. [PMID:7828851]
XLOC_047988	GO:0017111	nucleoside-triphosphatase activity	Catalysis of the reaction: a nucleoside triphosphate + H2O = nucleoside diphosphate + phosphate. [EC:3.6.1.15]
XLOC_054355	GO:0017111	nucleoside-triphosphatase activity	Catalysis of the reaction: a nucleoside triphosphate + H2O = nucleoside diphosphate + phosphate. [EC:3.6.1.15]
XLOC_071313	GO:0017111	nucleoside-triphosphatase activity	Catalysis of the reaction: a nucleoside triphosphate + H2O = nucleoside diphosphate + phosphate. [EC:3.6.1.15]
XLOC_032633	GO:0019243	methylglyoxal catabolic process to D-lactate	The chemical reactions and pathways resulting in the breakdown of methylglyoxal, CH3-CO-CHO, into D-lactate via the intermediate S-lactoyl-glutathione. Glutathione is used in the first step of the pathway and then regenerated in the second step. [GOC:ai]
XLOC_001252	GO:0030433	ER-associated ubiquitin-dependent protein catabolic process	The chemical reactions and pathways resulting in the breakdown of proteins transported from the endoplasmic reticulum and targeted to cytoplasmic proteasomes for degradation. This process acts on misfolded proteins as well as in the regulated degradation of correctly folded proteins. [GOC:mah, GOC:rb, PMID:14607247, PMID:19520858]

XLOC_003953	GO:0030433	ER-associated ubiquitin-dependent protein catabolic process	The chemical reactions and pathways resulting in the breakdown of proteins transported from the endoplasmic reticulum and targeted to cytoplasmic proteasomes for degradation. This process acts on misfolded proteins as well as in the regulated degradation of correctly folded proteins. [GOC:mah, GOC:rb, PMID:14607247, PMID:19520858]
XLOC_064848	GO:0031123	RNA 3'-end processing	Any process involved in forming the mature 3' end of an RNA molecule. [GOC:mah]
XLOC_023020	GO:0032973	amino acid export	The directed movement of amino acids out of a cell or organelle. [GOC:mah]
XLOC_022758	GO:0033542	fatty acid beta-oxidation, unsaturated, even number	A fatty acid beta-oxidation pathway by which fatty acids having cis-double bonds on even-numbered carbons are degraded. Fatty acid beta-oxidation begins with the addition of coenzyme A to a fatty acid, and ends when only two or three carbons remain (as acetyl-CoA or propionyl-CoA respectively). [GOC:mah, MetaCyc:PWY-5138]
XLOC_043736	GO:0033542	fatty acid beta-oxidation, unsaturated, even number	A fatty acid beta-oxidation pathway by which fatty acids having cis-double bonds on even-numbered carbons are degraded. Fatty acid beta-oxidation begins with the addition of coenzyme A to a fatty acid, and ends when only two or three carbons remain (as acetyl-CoA or propionyl-CoA respectively). [GOC:mah, MetaCyc:PWY-5138]
XLOC_024108	GO:0035091	phosphatidylinositol binding	Interacting selectively and non-covalently with any inositol-containing glycerophospholipid, i.e. phosphatidylinositol (PtdIns) and its phosphorylated derivatives. [GOC:bf, ISBN:0198506732, PMID:11395417]
XLOC_040357	GO:0035091	phosphatidylinositol binding	Interacting selectively and non-covalently with any inositol-containing glycerophospholipid, i.e. phosphatidylinositol (PtdIns) and its phosphorylated derivatives. [GOC:bf, ISBN:0198506732, PMID:11395417]
XLOC_007198	GO:0043531	ADP binding	Interacting selectively and non-covalently with ADP, adenosine 5'-diphosphate. [GOC:jl]
XLOC_054077	GO:0043531	ADP binding	Interacting selectively and non-covalently with ADP, adenosine 5'-diphosphate. [GOC:jl]
XLOC_003338	GO:0047940	glucuronokinase activity	Catalysis of the reaction: D-glucuronate + ATP = 1-phospho-alpha-D-glucuronate + ADP + 2 H(+). [EC:2.7.1.43, RHEA:17008]

XLOC_014220	GO:0047940	glucuronokinase activity	Catalysis of the reaction: D-glucuronate + ATP = 1-phospho-alpha-D-glucuronate + ADP + 2 H(+). [EC:2.7.1.43, RHEA:17008]
XLOC_018392	GO:0047940	glucuronokinase activity	Catalysis of the reaction: D-glucuronate + ATP = 1-phospho-alpha-D-glucuronate + ADP + 2 H(+). [EC:2.7.1.43, RHEA:17008]
XLOC_019586	GO:0047940	glucuronokinase activity	Catalysis of the reaction: D-glucuronate + ATP = 1-phospho-alpha-D-glucuronate + ADP + 2 H(+). [EC:2.7.1.43, RHEA:17008]
XLOC_025312	GO:0047940	glucuronokinase activity	Catalysis of the reaction: D-glucuronate + ATP = 1-phospho-alpha-D-glucuronate + ADP + 2 H(+). [EC:2.7.1.43, RHEA:17008]
XLOC_027543	GO:0047940	glucuronokinase activity	Catalysis of the reaction: D-glucuronate + ATP = 1-phospho-alpha-D-glucuronate + ADP + 2 H(+). [EC:2.7.1.43, RHEA:17008]
XLOC_031093	GO:0047940	glucuronokinase activity	Catalysis of the reaction: D-glucuronate + ATP = 1-phospho-alpha-D-glucuronate + ADP + 2 H(+). [EC:2.7.1.43, RHEA:17008]
XLOC_037399	GO:0047940	glucuronokinase activity	Catalysis of the reaction: D-glucuronate + ATP = 1-phospho-alpha-D-glucuronate + ADP + 2 H(+). [EC:2.7.1.43, RHEA:17008]
XLOC_041683	GO:0047940	glucuronokinase activity	Catalysis of the reaction: D-glucuronate + ATP = 1-phospho-alpha-D-glucuronate + ADP + 2 H(+). [EC:2.7.1.43, RHEA:17008]
XLOC_056178	GO:0047940	glucuronokinase activity	Catalysis of the reaction: D-glucuronate + ATP = 1-phospho-alpha-D-glucuronate + ADP + 2 H(+). [EC:2.7.1.43, RHEA:17008]
XLOC_058089	GO:0047940	glucuronokinase activity	Catalysis of the reaction: D-glucuronate + ATP = 1-phospho-alpha-D-glucuronate + ADP + 2 H(+). [EC:2.7.1.43, RHEA:17008]
XLOC_030611	GO:0048544	recognition of pollen	The process, involving the sharing and interaction of the single locus incompatibility haplotypes, involved in the recognition or rejection of the self pollen by cells in the stigma. This process ensures out-breeding in certain plant species. [GOC:dph, GOC:pj, GOC:tb]
XLOC_055417	GO:0051607	defense response to virus	Reactions triggered in response to the presence of a virus that act to protect the cell or organism. [GOC:ai]

XLOC_026644	GO:0070206	protein trimerization	The formation of a protein trimer, a macromolecular structure consisting of three noncovalently associated identical or nonidentical subunits. [GOC:hjd]
XLOC_018843	GO:0070652	HAUS complex	A protein complex that localizes to interphase centrosomes and to mitotic spindle tubules and regulates mitotic spindle assembly and centrosome integrity; in human, the complex consists of eight subunits, some of which are homologous to subunits of the Drosophila Augmin complex. [PMID:19427217]
XLOC_033922	GO:0070652	HAUS complex	A protein complex that localizes to interphase centrosomes and to mitotic spindle tubules and regulates mitotic spindle assembly and centrosome integrity; in human, the complex consists of eight subunits, some of which are homologous to subunits of the Drosophila Augmin complex. [PMID:19427217]
XLOC_060416	GO:0070652	HAUS complex	A protein complex that localizes to interphase centrosomes and to mitotic spindle tubules and regulates mitotic spindle assembly and centrosome integrity; in human, the complex consists of eight subunits, some of which are homologous to subunits of the Drosophila Augmin complex. [PMID:19427217]
XLOC_067881	GO:0070652	HAUS complex	A protein complex that localizes to interphase centrosomes and to mitotic spindle tubules and regulates mitotic spindle assembly and centrosome integrity; in human, the complex consists of eight subunits, some of which are homologous to subunits of the Drosophila Augmin complex. [PMID:19427217]
XLOC_041251	GO:0080027	response to herbivore	Any process that results in a change in state or activity of a cell or an organism (in terms of movement, secretion, enzyme production, gene expression, etc.) as a result of a stimulus from a herbivore. [PMID:18987211]
XLOC_045400	GO:0080148	negative regulation of response to water deprivation	Any process that stops, prevents, or reduces the frequency, rate or extent of a response to water deprivation. Response to water deprivation is a change in state or activity of a cell or an organism (in terms of movement, secretion, enzyme production, gene expression, etc.) as a result of a water deprivation stimulus, prolonged deprivation of water. [PMID:18835996]
XLOC_030020	GO:0080150	S-adenosyl-L-methionine:benzoic acid carboxyl methyl transferase activity	Catalysis of the reaction: benzoate + S-adenosyl-L-methionine = methylbenzoate + S-adenosyl-L-homocysteine. [MetaCyc:RXN-6722, PMID:10852939]
XLOC_030239	GO:0080150	S-adenosyl-L-methionine:benzoic acid carboxyl methyl transferase activity	Catalysis of the reaction: benzoate + S-adenosyl-L-methionine = methylbenzoate + S-adenosyl-L-homocysteine. [MetaCyc:RXN-6722, PMID:10852939]

XLOC_031403	GO:0080150	S-adenosyl-L-methionine:benzoic acid carboxyl methyl transferase activity	Catalysis of the reaction: benzoate + S-adenosyl-L-methionine = methylbenzoate + S-adenosyl-L-homocysteine. [MetaCyc:RXN-6722, PMID:10852939]
XLOC_033881	GO:0080150	S-adenosyl-L-methionine:benzoic acid carboxyl methyl transferase activity	Catalysis of the reaction: benzoate + S-adenosyl-L-methionine = methylbenzoate + S-adenosyl-L-homocysteine. [MetaCyc:RXN-6722, PMID:10852939]
XLOC_056226	GO:0080150	S-adenosyl-L-methionine:benzoic acid carboxyl methyl transferase activity	Catalysis of the reaction: benzoate + S-adenosyl-L-methionine = methylbenzoate + S-adenosyl-L-homocysteine. [MetaCyc:RXN-6722, PMID:10852939]
XLOC_064796	GO:0080150	S-adenosyl-L-methionine:benzoic acid carboxyl methyl transferase activity	Catalysis of the reaction: benzoate + S-adenosyl-L-methionine = methylbenzoate + S-adenosyl-L-homocysteine. [MetaCyc:RXN-6722, PMID:10852939]
XLOC_025619	GO:0090322	regulation of superoxide metabolic process	Any process that modulates the rate, frequency, or extent of superoxide metabolism, the chemical reactions and pathways involving superoxide, the superoxide anion O2- (superoxide free radical), or any compound containing this species. [GOC:tb]
XLOC_000245			
XLOC_000267			
XLOC_000398			
XLOC_001170			
XLOC_001434			
XLOC_001450			
XLOC_001802			
XLOC_001833			
XLOC_001879			
XLOC_001896			
XLOC_001965			
XLOC_002071			
XLOC_002340			
XLOC_002894			
XLOC_003227			
XLOC_003301			
XLOC_003792			
XLOC_004218			
XLOC_004428			
XLOC_004842			
XLOC_006237			
XLOC_006373			
XLOC_006410			
XLOC_006453			
XLOC_007624			

XLOC_007797
XLOC_007962
XLOC_008477
XLOC_008554
XLOC_008566
XLOC_008591
XLOC_008615
XLOC_008657
XLOC_008660
XLOC_008713
XLOC_008728
XLOC_008789
XLOC_008989
XLOC_009047
XLOC_009088
XLOC_009124
XLOC_009294
XLOC_009455
XLOC_009483
XLOC_009491
XLOC_009573
XLOC_010395
XLOC_010442
XLOC_010819
XLOC_010867
XLOC_011019
XLOC_011196
XLOC_011301
XLOC_011622
XLOC_012328
XLOC_012445
XLOC_012671
XLOC_012843
XLOC_013292
XLOC_013368
XLOC_013373
XLOC_013639
XLOC_013758
XLOC_013830
XLOC_013851
XLOC_013895
XLOC_013917

XLOC_013940
XLOC_013977
XLOC_014017
XLOC_014111
XLOC_014121
XLOC_014222
XLOC_014674
XLOC_014714
XLOC_015085
XLOC_015169
XLOC_015476
XLOC_015588
XLOC_015614
XLOC_015690
XLOC_015995
XLOC_016626
XLOC_017127
XLOC_017361
XLOC_017743
XLOC_018058
XLOC_018099
XLOC_018191
XLOC_018220
XLOC_018434
XLOC_018454
XLOC_018935
XLOC_019106
XLOC_019364
XLOC_019399
XLOC_019417
XLOC_019473
XLOC_019482
XLOC_019502
XLOC_019526
XLOC_019614
XLOC_019768
XLOC_019991
XLOC_020431
XLOC_020463
XLOC_020671
XLOC_020755
XLOC_020886

XLOC_021223
XLOC_021305
XLOC_021491
XLOC_021765
XLOC_021872
XLOC_022062
XLOC_022187
XLOC_022384
XLOC_022534
XLOC_022785
XLOC_023258
XLOC_023803
XLOC_023885
XLOC_023903
XLOC_024116
XLOC_024285
XLOC_024410
XLOC_024435
XLOC_024573
XLOC_024623
XLOC_024644
XLOC_024801
XLOC_024825
XLOC_024826
XLOC_024907
XLOC_025020
XLOC_025134
XLOC_025332
XLOC_025494
XLOC_025724
XLOC_025780
XLOC_025812
XLOC_025856
XLOC_025944
XLOC_026094
XLOC_026124
XLOC_026581
XLOC_026618
XLOC_026627
XLOC_026666
XLOC_027549
XLOC_027625

XLOC_027729
XLOC_027908
XLOC_028103
XLOC_028252
XLOC_028454
XLOC_028458
XLOC_028495
XLOC_028941
XLOC_029130
XLOC_029351
XLOC_029465
XLOC_029466
XLOC_029477
XLOC_029564
XLOC_029615
XLOC_029617
XLOC_030002
XLOC_030038
XLOC_030250
XLOC_030303
XLOC_030680
XLOC_030886
XLOC_031008
XLOC_031095
XLOC_031521
XLOC_031801
XLOC_032177
XLOC_032361
XLOC_032404
XLOC_032410
XLOC_032515
XLOC_032540
XLOC_032631
XLOC_032707
XLOC_032754
XLOC_032957
XLOC_033689
XLOC_033907
XLOC_034236
XLOC_034808
XLOC_034836
XLOC_035121

XLOC_035223
XLOC_035639
XLOC_035796
XLOC_036036
XLOC_036136
XLOC_036701
XLOC_037613
XLOC_037810
XLOC_037969
XLOC_039388
XLOC_039450
XLOC_039972
XLOC_040314
XLOC_040342
XLOC_040420
XLOC_040461
XLOC_040535
XLOC_040714
XLOC_040716
XLOC_040734
XLOC_040783
XLOC_040827
XLOC_040871
XLOC_040963
XLOC_041079
XLOC_041106
XLOC_041250
XLOC_042270
XLOC_042284
XLOC_042345
XLOC_042449
XLOC_042875
XLOC_042926
XLOC_042951
XLOC_043351
XLOC_043389
XLOC_043457
XLOC_043564
XLOC_043766
XLOC_043810
XLOC_043932
XLOC_043958

XLOC_043980
XLOC_044242
XLOC_044515
XLOC_044635
XLOC_045026
XLOC_045190
XLOC_045455
XLOC_045461
XLOC_045665
XLOC_046295
XLOC_046568
XLOC_047013
XLOC_047081
XLOC_047592
XLOC_047716
XLOC_047717
XLOC_047746
XLOC_047875
XLOC_047986
XLOC_048281
XLOC_048675
XLOC_049042
XLOC_049495
XLOC_049563
XLOC_049823
XLOC_049837
XLOC_050565
XLOC_050950
XLOC_051624
XLOC_051986
XLOC_052319
XLOC_053386
XLOC_053440
XLOC_053534
XLOC_053622
XLOC_053667
XLOC_053839
XLOC_054002
XLOC_054066
XLOC_054125
XLOC_054513
XLOC_054850

XLOC_054985
XLOC_055222
XLOC_055774
XLOC_055908
XLOC_056246
XLOC_056566
XLOC_056656
XLOC_056671
XLOC_056911
XLOC_057170
XLOC_057525
XLOC_057659
XLOC_057842
XLOC_057845
XLOC_058135
XLOC_058982
XLOC_059063
XLOC_059517
XLOC_059894
XLOC_060017
XLOC_060298
XLOC_060300
XLOC_060301
XLOC_060303
XLOC_060304
XLOC_060305
XLOC_060306
XLOC_060438
XLOC_060439
XLOC_060472
XLOC_060547
XLOC_060947
XLOC_061693
XLOC_061882
XLOC_062612
XLOC_062698
XLOC_063414
XLOC_063523
XLOC_063803
XLOC_063811
XLOC_064584
XLOC_064585

XLOC_064620
XLOC_064693
XLOC_065044
XLOC_065750
XLOC_065967
XLOC_066010
XLOC_066141
XLOC_066198
XLOC_066345
XLOC_066417
XLOC_066419
XLOC_066431
XLOC_066443
XLOC_066486
XLOC_066507
XLOC_066606
XLOC_066753
XLOC_066826
XLOC_066861
XLOC_066869
XLOC_066977
XLOC_067155
XLOC_067278
XLOC_067721
XLOC_067752
XLOC_068237
XLOC_068343
XLOC_068683
XLOC_068826
XLOC_069227
XLOC_069337
XLOC_069353
XLOC_069387
XLOC_070203
XLOC_070227
XLOC_070321
XLOC_070774
XLOC_071006
XLOC_071147
XLOC_071318
XLOC_071724
XLOC_071795

XLOC_071799

XLOC_071842

XLOC_072104

XLOC_072179

XLOC_072536

XLOC_072614

XLOC_072682

XLOC_072903

XLOC_073344

XLOC_073610

XLOC_073764

XLOC_073953

XLOC_074256

XLOC_075932

XLOC_076030

XLOC_076344

XLOC_076691

XLOC_076740

XLOC_076742

XLOC_076778

XLOC_076779

XLOC_076839

XLOC_076889

XLOC_076894

XLOC_076919

XLOC_077089

XLOC_077108

XLOC_077146

XLOC_077209

Table S4. Annotations of the LincRNAs in Rice Shoot

LincRNA	GO ID	GO name	GO description
XLOC_067155	GO:0030029	actin filament-based process	Any cellular process that depends upon or alters the actin cytoskeleton, that part of the cytoskeleton comprising actin filaments and their associated proteins. [GOC:mah]
XLOC_037328	GO:0016832	aldehyde-lyase activity	Catalysis of the cleavage of a C-C bond in a molecule containing a hydroxyl group and a carbonyl group to form two smaller molecules, each being an aldehyde or a ketone. [http://www.mercksource.com/]
XLOC_035920	GO:0004045	aminoacyl-tRNA hydrolase activity	Catalysis of the reaction: N-substituted aminoacyl-tRNA + H2O = N-substituted amino acid + tRNA. [EC:3.1.1.29]
XLOC_043736	GO:0004045	aminoacyl-tRNA hydrolase activity	Catalysis of the reaction: N-substituted aminoacyl-tRNA + H2O = N-substituted amino acid + tRNA. [EC:3.1.1.29]
XLOC_027908	GO:0008026	ATP-dependent helicase activity	Catalysis of the reaction: ATP + H2O = ADP + phosphate, to drive the unwinding of a DNA or RNA helix. [EC:3.6.1.3, GOC:jl]
XLOC_025912	GO:0004004	ATP-dependent RNA helicase activity	Catalysis of the reaction: ATP + H2O = ADP + phosphate; this reaction drives the unwinding of an RNA helix. [EC:3.6.1.3, GOC:jl]

XLOC_047650	GO:0005488	binding	The selective, non-covalent, often stoichiometric, interaction of a molecule with one or more specific sites on another molecule. [GOC:ceb, GOC:mah, ISBN:0198506732]
XLOC_071461	GO:0016602	CCAAT-binding factor complex	A heteromeric transcription factor complex that binds to the CCAAT-box upstream of promoters; in Saccharomyces it activates the transcription of genes in response to growth in a nonfermentable carbon source; consists of four known subunits: HAP2, HAP3, HAP4 and HAP5. [PMID:7828851]
XLOC_012445	GO:0019725	cellular homeostasis	Any process involved in the maintenance of an internal steady state at the level of the cell. [GOC:isa_complete, GOC:jl, ISBN:0395825172]
XLOC_001879	GO:0071368	cellular response to cytokinin stimulus	Any process that results in a change in state or activity of a cell (in terms of movement, secretion, enzyme production, gene expression, etc.) as a result of a cytokinin stimulus. [GOC:mah]
XLOC_021899	GO:0071368	cellular response to cytokinin stimulus	Any process that results in a change in state or activity of a cell (in terms of movement, secretion, enzyme production, gene expression, etc.) as a result of a cytokinin stimulus. [GOC:mah]
XLOC_031093	GO:0071368	cellular response to cytokinin stimulus	Any process that results in a change in state or activity of a cell (in terms of movement, secretion, enzyme production, gene expression, etc.) as a result of a cytokinin stimulus. [GOC:mah]
XLOC_037810	GO:0071368	cellular response to cytokinin stimulus	Any process that results in a change in state or activity of a cell (in terms of movement, secretion, enzyme production, gene expression, etc.) as a result of a cytokinin stimulus. [GOC:mah]

XLOC_040486	GO:0071368	cellular response to cytokinin stimulus	Any process that results in a change in state or activity of a cell (in terms of movement, secretion, enzyme production, gene expression, etc.) as a result of a cytokinin stimulus. [GOC:mah]
XLOC_063523	GO:0071368	cellular response to cytokinin stimulus	Any process that results in a change in state or activity of a cell (in terms of movement, secretion, enzyme production, gene expression, etc.) as a result of a cytokinin stimulus. [GOC:mah]
XLOC_059805	GO:0071281	cellular response to iron ion	Any process that results in a change in state or activity of a cell (in terms of movement, secretion, enzyme production, gene expression, etc.) as a result of an iron ion stimulus. [GOC:mah]
XLOC_031802	GO:0004568	chitinase activity	Catalysis of the hydrolysis of (1->4)-beta linkages of N-acetyl-D-glucosamine (GlcNAc) polymers of chitin and chitodextrins. [EC:3.2.1.14, GOC:bf, GOC:kah, GOC:pde, PMID:11468293]
XLOC_022758	GO:0017004	cytochrome complex assembly	The aggregation, arrangement and bonding together of a cytochrome complex. A cytochrome complex is a protein complex in which at least one of the proteins is a cytochrome, i.e. a heme-containing protein involved in catalysis of redox reactions. [GOC:jl, GOC:mah]
XLOC_046102	GO:0017004	cytochrome complex assembly	The aggregation, arrangement and bonding together of a cytochrome complex. A cytochrome complex is a protein complex in which at least one of the proteins is a cytochrome, i.e. a heme-containing protein involved in catalysis of redox reactions. [GOC:jl, GOC:mah]
XLOC_047552	GO:0017004	cytochrome complex assembly	The aggregation, arrangement and bonding together of a cytochrome complex. A cytochrome complex is a protein complex in which at least one of the proteins is a cytochrome, i.e. a heme-containing protein involved in catalysis of redox reactions. [GOC:jl, GOC:mah]

XLOC_048059	GO:0017004	cytochrome complex assembly	The aggregation, arrangement and bonding together of a cytochrome complex. A cytochrome complex is a protein complex in which at least one of the proteins is a cytochrome, i.e. a heme-containing protein involved in catalysis of redox reactions. [GOC:jl, GOC:mah]
XLOC_047013	GO:0051213	dioxygenase activity	Catalysis of an oxidation-reduction (redox) reaction in which both atoms of oxygen from one molecule of O2 are incorporated into the (reduced) product(s) of the reaction. The two atoms of oxygen may be distributed between two different products. [DOI:10.1016/S0040-4020(03)00944-X, GOC:bf, http://www.onelook.com/]
XLOC_068207	GO:0008725	DNA-3-methyladenine glycosylase activity	Catalysis of the reaction: DNA containing 3-methyladenine + H2O = DNA with abasic site + 3-methyladenine. This reaction is the hydrolysis of DNA by cleavage of the N-C1' glycosidic bond between the damaged DNA 3-methyladenine and the deoxyribose sugar to remove the 3-methyladenine, leaving an abasic site. [EC:3.2.2.20, GOC:elh, PMID:10872450, PMID:9224623]
XLOC_068493	GO:0072546	ER membrane protein complex	A transmembrane protein complex that is involved in protein folding in the endoplasmic reticulum. In S. cerevisiae, it has six members: EMC1, EMC2, AIM27, EMC4, KRE27, and EMC6. [GOC:dgf, PMID:19325107]
XLOC_015320	GO:0004325	ferrochelatase activity	Catalysis of the reaction: protoheme = Fe(2+) + protoporphyrin IX. [EC:4.99.1.1, RHEA:22587]
XLOC_034785	GO:0004325	ferrochelatase activity	Catalysis of the reaction: protoheme = Fe(2+) + protoporphyrin IX. [EC:4.99.1.1, RHEA:22587]
XLOC_040535	GO:0004325	ferrochelatase activity	Catalysis of the reaction: protoheme = Fe(2+) + protoporphyrin IX. [EC:4.99.1.1, RHEA:22587]

XLOC_060644	GO:0004325	ferrochelatase activity	Catalysis of the reaction: protoheme = Fe(2+) + protoporphyrin IX. [EC:4.99.1.1, RHEA:22587]
XLOC_020272	GO:0005528	FK506 binding	Interacting selectively and non-covalently with the immunosuppressant FK506. [GOC:jl]
XLOC_022927	GO:0005528	FK506 binding	Interacting selectively and non-covalently with the immunosuppressant FK506. [GOC:jl]
XLOC_035326	GO:0005528	FK506 binding	Interacting selectively and non-covalently with the immunosuppressant FK506. [GOC:jl]
XLOC_061908	GO:0006680	glucosylceramide catabolic process	The chemical reactions and pathways resulting in the breakdown of glucosylceramides, any compound formed by the replacement of the glycosidic hydroxyl group of a cyclic form of glucose by a ceramide group. [GOC:ai]
XLOC_014220	GO:0047940	glucuronokinase activity	Catalysis of the reaction: D-glucuronate + ATP = 1-phospho-alpha-D-glucuronate + ADP + 2 H(+). [EC:2.7.1.43, RHEA:17008]
XLOC_025812	GO:0070652	HAUS complex	A protein complex that localizes to interphase centrosomes and to mitotic spindle tubules and regulates mitotic spindle assembly and centrosome integrity; in human, the complex consists of eight subunits, some of which are homologous to subunits of the Drosophila Augmin complex. [PMID:19427217]

XLOC_026618	GO:0070652	HAUS complex	A protein complex that localizes to interphase centrosomes and to mitotic spindle tubules and regulates mitotic spindle assembly and centrosome integrity; in human, the complex consists of eight subunits, some of which are homologous to subunits of the Drosophila Augmin complex. [PMID:19427217]
XLOC_027447	GO:0070652	HAUS complex	A protein complex that localizes to interphase centrosomes and to mitotic spindle tubules and regulates mitotic spindle assembly and centrosome integrity; in human, the complex consists of eight subunits, some of which are homologous to subunits of the Drosophila Augmin complex. [PMID:19427217]
XLOC_028941	GO:0070652	HAUS complex	A protein complex that localizes to interphase centrosomes and to mitotic spindle tubules and regulates mitotic spindle assembly and centrosome integrity; in human, the complex consists of eight subunits, some of which are homologous to subunits of the Drosophila Augmin complex. [PMID:19427217]
XLOC_040461	GO:0070652	HAUS complex	A protein complex that localizes to interphase centrosomes and to mitotic spindle tubules and regulates mitotic spindle assembly and centrosome integrity; in human, the complex consists of eight subunits, some of which are homologous to subunits of the Drosophila Augmin complex. [PMID:19427217]
XLOC_047345	GO:0070652	HAUS complex	A protein complex that localizes to interphase centrosomes and to mitotic spindle tubules and regulates mitotic spindle assembly and centrosome integrity; in human, the complex consists of eight subunits, some of which are homologous to subunits of the Drosophila Augmin complex. [PMID:19427217]
XLOC_054892	GO:0070652	HAUS complex	A protein complex that localizes to interphase centrosomes and to mitotic spindle tubules and regulates mitotic spindle assembly and centrosome integrity; in human, the complex consists of eight subunits, some of which are homologous to subunits of the Drosophila Augmin complex. [PMID:19427217]
XLOC_076778	GO:0070652	HAUS complex	A protein complex that localizes to interphase centrosomes and to mitotic spindle tubules and regulates mitotic spindle assembly and centrosome integrity; in human, the complex consists of eight subunits, some of which are homologous to subunits of the Drosophila Augmin complex. [PMID:19427217]

XLOC_062234	GO:0010452	histone H3-K36 methylation	The modification of histone H3 by addition of a methyl group to lysine at position 36 of the histone. [GOC:tb]
XLOC_054077	GO:0006972	hyperosmotic response	Any process that results in a change in state or activity of a cell or an organism (in terms of movement, secretion, enzyme production, gene expression, etc.) as a result of detection of, or exposure to, a hyperosmotic environment, i.e. an environment with a higher concentration of solutes than the organism or cell. [GOC:jl, PMID:12142009]
XLOC_010433	GO:0008610	lipid biosynthetic process	The chemical reactions and pathways resulting in the formation of lipids, compounds soluble in an organic solvent but not, or sparingly, in an aqueous solvent. [GOC:go_curators]
XLOC_010819	GO:0008610	lipid biosynthetic process	The chemical reactions and pathways resulting in the formation of lipids, compounds soluble in an organic solvent but not, or sparingly, in an aqueous solvent. [GOC:go_curators]
XLOC_061072	GO:0008610	lipid biosynthetic process	The chemical reactions and pathways resulting in the formation of lipids, compounds soluble in an organic solvent but not, or sparingly, in an aqueous solvent. [GOC:go_curators]
XLOC_073131	GO:0008610	lipid biosynthetic process	The chemical reactions and pathways resulting in the formation of lipids, compounds soluble in an organic solvent but not, or sparingly, in an aqueous solvent. [GOC:go_curators]
XLOC_000697	GO:0006869	lipid transport	The directed movement of lipids into, out of or within a cell, or between cells, by means of some agent such as a transporter or pore. Lipids are compounds soluble in an organic solvent but not, or sparingly, in an aqueous solvent. [ISBN:0198506732]

XLOC_008741	GO:0006869	lipid transport	The directed movement of lipids into, out of or within a cell, or between cells, by means of some agent such as a transporter or pore. Lipids are compounds soluble in an organic solvent but not, or sparingly, in an aqueous solvent. [ISBN:0198506732]
XLOC_018099	GO:0006869	lipid transport	The directed movement of lipids into, out of or within a cell, or between cells, by means of some agent such as a transporter or pore. Lipids are compounds soluble in an organic solvent but not, or sparingly, in an aqueous solvent. [ISBN:0198506732]
XLOC_024209	GO:0006869	lipid transport	The directed movement of lipids into, out of or within a cell, or between cells, by means of some agent such as a transporter or pore. Lipids are compounds soluble in an organic solvent but not, or sparingly, in an aqueous solvent. [ISBN:0198506732]
XLOC_024435	GO:0006869	lipid transport	The directed movement of lipids into, out of or within a cell, or between cells, by means of some agent such as a transporter or pore. Lipids are compounds soluble in an organic solvent but not, or sparingly, in an aqueous solvent. [ISBN:0198506732]
XLOC_025834	GO:0006869	lipid transport	The directed movement of lipids into, out of or within a cell, or between cells, by means of some agent such as a transporter or pore. Lipids are compounds soluble in an organic solvent but not, or sparingly, in an aqueous solvent. [ISBN:0198506732]
XLOC_026516	GO:0006869	lipid transport	The directed movement of lipids into, out of or within a cell, or between cells, by means of some agent such as a transporter or pore. Lipids are compounds soluble in an organic solvent but not, or sparingly, in an aqueous solvent. [ISBN:0198506732]
XLOC_030698	GO:0006869	lipid transport	The directed movement of lipids into, out of or within a cell, or between cells, by means of some agent such as a transporter or pore. Lipids are compounds soluble in an organic solvent but not, or sparingly, in an aqueous solvent. [ISBN:0198506732]

XLOC_031095	GO:0006869	lipid transport	The directed movement of lipids into, out of or within a cell, or between cells, by means of some agent such as a transporter or pore. Lipids are compounds soluble in an organic solvent but not, or sparingly, in an aqueous solvent. [ISBN:0198506732]
XLOC_033881	GO:0006869	lipid transport	The directed movement of lipids into, out of or within a cell, or between cells, by means of some agent such as a transporter or pore. Lipids are compounds soluble in an organic solvent but not, or sparingly, in an aqueous solvent. [ISBN:0198506732]
XLOC_039388	GO:0006869	lipid transport	The directed movement of lipids into, out of or within a cell, or between cells, by means of some agent such as a transporter or pore. Lipids are compounds soluble in an organic solvent but not, or sparingly, in an aqueous solvent. [ISBN:0198506732]
XLOC_044441	GO:0006869	lipid transport	The directed movement of lipids into, out of or within a cell, or between cells, by means of some agent such as a transporter or pore. Lipids are compounds soluble in an organic solvent but not, or sparingly, in an aqueous solvent. [ISBN:0198506732]
XLOC_044456	GO:0006869	lipid transport	The directed movement of lipids into, out of or within a cell, or between cells, by means of some agent such as a transporter or pore. Lipids are compounds soluble in an organic solvent but not, or sparingly, in an aqueous solvent. [ISBN:0198506732]
XLOC_048675	GO:0006869	lipid transport	The directed movement of lipids into, out of or within a cell, or between cells, by means of some agent such as a transporter or pore. Lipids are compounds soluble in an organic solvent but not, or sparingly, in an aqueous solvent. [ISBN:0198506732]
XLOC_057525	GO:0006869	lipid transport	The directed movement of lipids into, out of or within a cell, or between cells, by means of some agent such as a transporter or pore. Lipids are compounds soluble in an organic solvent but not, or sparingly, in an aqueous solvent. [ISBN:0198506732]

XLOC_057659	GO:0006869	lipid transport	The directed movement of lipids into, out of or within a cell, or between cells, by means of some agent such as a transporter or pore. Lipids are compounds soluble in an organic solvent but not, or sparingly, in an aqueous solvent. [ISBN:0198506732]
XLOC_057869	GO:0006869	lipid transport	The directed movement of lipids into, out of or within a cell, or between cells, by means of some agent such as a transporter or pore. Lipids are compounds soluble in an organic solvent but not, or sparingly, in an aqueous solvent. [ISBN:0198506732]
XLOC_058915	GO:0006869	lipid transport	The directed movement of lipids into, out of or within a cell, or between cells, by means of some agent such as a transporter or pore. Lipids are compounds soluble in an organic solvent but not, or sparingly, in an aqueous solvent. [ISBN:0198506732]
XLOC_059517	GO:0006869	lipid transport	The directed movement of lipids into, out of or within a cell, or between cells, by means of some agent such as a transporter or pore. Lipids are compounds soluble in an organic solvent but not, or sparingly, in an aqueous solvent. [ISBN:0198506732]
XLOC_077187	GO:0006869	lipid transport	The directed movement of lipids into, out of or within a cell, or between cells, by means of some agent such as a transporter or pore. Lipids are compounds soluble in an organic solvent but not, or sparingly, in an aqueous solvent. [ISBN:0198506732]
XLOC_065020	GO:0015095	magnesium ion transmembrane transporter activity	Catalysis of the transfer of magnesium (Mg) ions from one side of a membrane to the other. [GOC:dgf]
XLOC_013369	GO:0033615	mitochondrial proton-transporting ATP synthase complex assembly	The aggregation, arrangement and bonding together of a proton-transporting ATP synthase in the mitochondrial inner membrane. [GOC:mah]

XLOC_030613	GO:0003774	motor activity	Catalysis of movement along a polymeric molecule such as a microfilament or microtubule, coupled to the hydrolysis of a nucleoside triphosphate. [GOC:mah, ISBN:0815316194]
XLOC_070228	GO:0003774	motor activity	Catalysis of movement along a polymeric molecule such as a microfilament or microtubule, coupled to the hydrolysis of a nucleoside triphosphate. [GOC:mah, ISBN:0815316194]
XLOC_025724	GO:0005847	mRNA cleavage and polyadenylation specificity factor complex	A multisubunit complex that binds to the canonical AAUAAA hexamer and to U-rich upstream sequence elements on the pre-mRNA, thereby stimulating the otherwise weakly active and nonspecific polymerase to elongate efficiently RNAs containing a poly(A) signal. [PMID:14749727]
XLOC_002113	GO:0006378	mRNA polyadenylation	The enzymatic addition of a sequence of 40-200 adenylyl residues at the 3' end of a eukaryotic mRNA primary transcript. [ISBN:0198506732]
XLOC_006373	GO:0006378	mRNA polyadenylation	The enzymatic addition of a sequence of 40-200 adenylyl residues at the 3' end of a eukaryotic mRNA primary transcript. [ISBN:0198506732]
XLOC_011133	GO:0006378	mRNA polyadenylation	The enzymatic addition of a sequence of 40-200 adenylyl residues at the 3' end of a eukaryotic mRNA primary transcript. [ISBN:0198506732]
XLOC_015818	GO:0006378	mRNA polyadenylation	The enzymatic addition of a sequence of 40-200 adenylyl residues at the 3' end of a eukaryotic mRNA primary transcript. [ISBN:0198506732]

XLOC_016946	GO:0006378	mRNA polyadenylation	The enzymatic addition of a sequence of 40-200 adenylyl residues at the 3' end of a eukaryotic mRNA primary transcript. [ISBN:0198506732]
XLOC_019886	GO:0006378	mRNA polyadenylation	The enzymatic addition of a sequence of 40-200 adenylyl residues at the 3' end of a eukaryotic mRNA primary transcript. [ISBN:0198506732]
XLOC_021491	GO:0006378	mRNA polyadenylation	The enzymatic addition of a sequence of 40-200 adenylyl residues at the 3' end of a eukaryotic mRNA primary transcript. [ISBN:0198506732]
XLOC_025619	GO:0006378	mRNA polyadenylation	The enzymatic addition of a sequence of 40-200 adenylyl residues at the 3' end of a eukaryotic mRNA primary transcript. [ISBN:0198506732]
XLOC_031943	GO:0006378	mRNA polyadenylation	The enzymatic addition of a sequence of 40-200 adenylyl residues at the 3' end of a eukaryotic mRNA primary transcript. [ISBN:0198506732]
XLOC_037613	GO:0006378	mRNA polyadenylation	The enzymatic addition of a sequence of 40-200 adenylyl residues at the 3' end of a eukaryotic mRNA primary transcript. [ISBN:0198506732]
XLOC_041106	GO:0006378	mRNA polyadenylation	The enzymatic addition of a sequence of 40-200 adenylyl residues at the 3' end of a eukaryotic mRNA primary transcript. [ISBN:0198506732]

XLOC_046295	GO:0006378	mRNA polyadenylation	The enzymatic addition of a sequence of 40-200 adenylyl residues at the 3' end of a eukaryotic mRNA primary transcript. [ISBN:0198506732]
XLOC_047346	GO:0006378	mRNA polyadenylation	The enzymatic addition of a sequence of 40-200 adenylyl residues at the 3' end of a eukaryotic mRNA primary transcript. [ISBN:0198506732]
XLOC_048281	GO:0006378	mRNA polyadenylation	The enzymatic addition of a sequence of 40-200 adenylyl residues at the 3' end of a eukaryotic mRNA primary transcript. [ISBN:0198506732]
XLOC_049097	GO:0006378	mRNA polyadenylation	The enzymatic addition of a sequence of 40-200 adenylyl residues at the 3' end of a eukaryotic mRNA primary transcript. [ISBN:0198506732]
XLOC_051599	GO:0006378	mRNA polyadenylation	The enzymatic addition of a sequence of 40-200 adenylyl residues at the 3' end of a eukaryotic mRNA primary transcript. [ISBN:0198506732]
XLOC_053044	GO:0006378	mRNA polyadenylation	The enzymatic addition of a sequence of 40-200 adenylyl residues at the 3' end of a eukaryotic mRNA primary transcript. [ISBN:0198506732]
XLOC_053386	GO:0006378	mRNA polyadenylation	The enzymatic addition of a sequence of 40-200 adenylyl residues at the 3' end of a eukaryotic mRNA primary transcript. [ISBN:0198506732]

XLOC_054628	GO:0006378	mRNA polyadenylation	The enzymatic addition of a sequence of 40-200 adenylyl residues at the 3' end of a eukaryotic mRNA primary transcript. [ISBN:0198506732]
XLOC_068645	GO:0006378	mRNA polyadenylation	The enzymatic addition of a sequence of 40-200 adenylyl residues at the 3' end of a eukaryotic mRNA primary transcript. [ISBN:0198506732]
XLOC_069348	GO:0006378	mRNA polyadenylation	The enzymatic addition of a sequence of 40-200 adenylyl residues at the 3' end of a eukaryotic mRNA primary transcript. [ISBN:0198506732]
XLOC_057842	GO:0048354	mucilage biosynthetic process involved in seed coat development	The chemical reactions and pathways resulting in the formation of mucilage that occur as part of seed coat development; mucilage is normally synthesized during seed coat development. [GOC:dph, GOC:jid, GOC:tb]
XLOC_001874	GO:0045792	negative regulation of cell size	Any process that reduces cell size. [GOC:go_curators]
XLOC_019586	GO:0045792	negative regulation of cell size	Any process that reduces cell size. [GOC:go_curators]
XLOC_034236	GO:0045792	negative regulation of cell size	Any process that reduces cell size. [GOC:go_curators]

XLOC_064796	GO:0045792	negative regulation of cell size	Any process that reduces cell size. [GOC:go_curators]
XLOC_067697	GO:0045792	negative regulation of cell size	Any process that reduces cell size. [GOC:go_curators]
XLOC_045400	GO:0080148	negative regulation of response to water deprivation	Any process that stops, prevents, or reduces the frequency, rate or extent of a response to water deprivation. Response to water deprivation is a change in state or activity of a cell or an organism (in terms of movement, secretion, enzyme production, gene expression, etc.) as a result of a water deprivation stimulus, prolonged deprivation of water. [PMID:18835996]
XLOC_075749	GO:0080148	negative regulation of response to water deprivation	Any process that stops, prevents, or reduces the frequency, rate or extent of a response to water deprivation. Response to water deprivation is a change in state or activity of a cell or an organism (in terms of movement, secretion, enzyme production, gene expression, etc.) as a result of a water deprivation stimulus, prolonged deprivation of water. [PMID:18835996]
XLOC_029671	GO:0000956	nuclear-transcribed mRNA catabolic process	The chemical reactions and pathways resulting in the breakdown of nuclear-transcribed mRNAs in eukaryotic cells. [GOC:krc]
XLOC_023258	GO:0003676	nucleic acid binding	Interacting selectively and non-covalently with any nucleic acid. [GOC:jl]
XLOC_040714	GO:0003676	nucleic acid binding	Interacting selectively and non-covalently with any nucleic acid. [GOC:jl]

XLOC_040716	GO:0003676	nucleic acid binding	Interacting selectively and non-covalently with any nucleic acid. [GOC:jl]
XLOC_060298	GO:0003676	nucleic acid binding	Interacting selectively and non-covalently with any nucleic acid. [GOC:jl]
XLOC_060300	GO:0003676	nucleic acid binding	Interacting selectively and non-covalently with any nucleic acid. [GOC:jl]
XLOC_060301	GO:0003676	nucleic acid binding	Interacting selectively and non-covalently with any nucleic acid. [GOC:jl]
XLOC_060303	GO:0003676	nucleic acid binding	Interacting selectively and non-covalently with any nucleic acid. [GOC:jl]
XLOC_060304	GO:0003676	nucleic acid binding	Interacting selectively and non-covalently with any nucleic acid. [GOC:jl]
XLOC_060305	GO:0003676	nucleic acid binding	Interacting selectively and non-covalently with any nucleic acid. [GOC:jl]

XLOC_076740	GO:0003676	nucleic acid binding	Interacting selectively and non-covalently with any nucleic acid. [GOC:jl]
XLOC_077209	GO:0003676	nucleic acid binding	Interacting selectively and non-covalently with any nucleic acid. [GOC:jl]
XLOC_037969	GO:0006656	phosphatidylcholine biosynthetic process	The chemical reactions and pathways resulting in the formation of phosphatidylcholines, any of a class of glycerophospholipids in which the phosphatidyl group is esterified to the hydroxyl group of choline. [ISBN:0198506732]
XLOC_045026	GO:0006656	phosphatidylcholine biosynthetic process	The chemical reactions and pathways resulting in the formation of phosphatidylcholines, any of a class of glycerophospholipids in which the phosphatidyl group is esterified to the hydroxyl group of choline. [ISBN:0198506732]
XLOC_056566	GO:0006656	phosphatidylcholine biosynthetic process	The chemical reactions and pathways resulting in the formation of phosphatidylcholines, any of a class of glycerophospholipids in which the phosphatidyl group is esterified to the hydroxyl group of choline. [ISBN:0198506732]
XLOC_073764	GO:0009505	plant-type cell wall	A more or less rigid stucture lying outside the cell membrane of a cell and composed of cellulose and pectin and other organic and inorganic substances. [ISBN:0471245208]
XLOC_056004	GO:0055035	plastid thylakoid membrane	The lipid bilayer membrane of any thylakoid within a plastid. [GOC:jid, GOC:rph]

XLOC_002340	GO:0016272	prefoldin complex	A multisubunit chaperone that is capable of delivering unfolded proteins to cytosolic chaperonin, which it acts as a cofactor for. In humans, the complex is a heterohexamer of two PFD-alpha and four PFD-beta type subunits. In Saccharomyces cerevisiae, it also acts in the nucleus to regulate the rate of elongation by RNA polymerase II via a direct effect on histone dynamics. [GOC:jl, PMID:17384227, PMID:24068951, PMID:9630229]
XLOC_033689	GO:0004656	procollagen-proline 4-dioxygenase activity	Catalysis of the reaction: procollagen L-proline + 2-oxoglutarate + O2 = procollagen trans-4-hydroxy-L-proline + succinate + CO2. [EC:1.14.11.2]
XLOC_019266	GO:0005515	protein binding	Interacting selectively and non-covalently with any protein or protein complex (a complex of two or more proteins that may include other nonprotein molecules). [GOC:go_curators]
XLOC_042345	GO:0005515	protein binding	Interacting selectively and non-covalently with any protein or protein complex (a complex of two or more proteins that may include other nonprotein molecules). [GOC:go_curators]
XLOC_047572	GO:0005515	protein binding	Interacting selectively and non-covalently with any protein or protein complex (a complex of two or more proteins that may include other nonprotein molecules). [GOC:go_curators]
XLOC_065652	GO:0009249	protein lipoylation	The addition of a lipoyl group to an amino acid residue in a protein. [GOC:mah]
XLOC_062487	GO:0004674	protein serine/threonine kinase activity	Catalysis of the reactions: ATP + protein serine = ADP + protein serine phosphate, and ATP + protein threonine = ADP + protein threonine phosphate. [GOC:bf]

XLOC_026644	GO:0070206	protein trimerization	The formation of a protein trimer, a macromolecular structure consisting of three noncovalently associated identical or nonidentical subunits. [GOC:hjd]
XLOC_036022	GO:0009982	pseudouridine synthase activity	Catalysis of the reaction: RNA uridine = RNA pseudouridine. Conversion of uridine in an RNA molecule to pseudouridine by rotation of the C1'-N-1 glycosidic bond of uridine in RNA to a C1'-C5. [EC:5.4.99.12, GOC:mah]
XLOC_009325	GO:0009787	regulation of abscisic acid-activated signaling pathway	Any process that modulates the frequency, rate or extent of abscisic acid (ABA) signaling. [GOC:lr]
XLOC_011019	GO:0009787	regulation of abscisic acid-activated signaling pathway	Any process that modulates the frequency, rate or extent of abscisic acid (ABA) signaling. [GOC:lr]
XLOC_027543	GO:0009787	regulation of abscisic acid-activated signaling pathway	Any process that modulates the frequency, rate or extent of abscisic acid (ABA) signaling. [GOC:lr]
XLOC_032273	GO:0009787	regulation of abscisic acid-activated signaling pathway	Any process that modulates the frequency, rate or extent of abscisic acid (ABA) signaling. [GOC:lr]
XLOC_036449	GO:0009787	regulation of abscisic acid-activated signaling pathway	Any process that modulates the frequency, rate or extent of abscisic acid (ABA) signaling. [GOC:lr]

XLOC_042926	GO:0009787	regulation of abscisic acid-activated signaling pathway	Any process that modulates the frequency, rate or extent of abscisic acid (ABA) signaling. [GOC:lr]
XLOC_043084	GO:0009787	regulation of abscisic acid-activated signaling pathway	Any process that modulates the frequency, rate or extent of abscisic acid (ABA) signaling. [GOC:lr]
XLOC_045455	GO:0009787	regulation of abscisic acid-activated signaling pathway	Any process that modulates the frequency, rate or extent of abscisic acid (ABA) signaling. [GOC:lr]
XLOC_051624	GO:0009787	regulation of abscisic acid-activated signaling pathway	Any process that modulates the frequency, rate or extent of abscisic acid (ABA) signaling. [GOC:lr]
XLOC_059443	GO:0009787	regulation of abscisic acid-activated signaling pathway	Any process that modulates the frequency, rate or extent of abscisic acid (ABA) signaling. [GOC:lr]
XLOC_070227	GO:0009787	regulation of abscisic acid-activated signaling pathway	Any process that modulates the frequency, rate or extent of abscisic acid (ABA) signaling. [GOC:lr]
XLOC_074955	GO:0009787	regulation of abscisic acid-activated signaling pathway	Any process that modulates the frequency, rate or extent of abscisic acid (ABA) signaling. [GOC:lr]

XLOC_013940	GO:0050776	regulation of immune response	Any process that modulates the frequency, rate or extent of the immune response, the immunological reaction of an organism to an immunogenic stimulus. [GOC:ai]
XLOC_030020	GO:0009991	response to extracellular stimulus	Any process that results in a change in state or activity of a cell or an organism (in terms of movement, secretion, enzyme production, gene expression, etc.) as a result of an extracellular stimulus. [GOC:go_curators]
XLOC_054224	GO:0009991	response to extracellular stimulus	Any process that results in a change in state or activity of a cell or an organism (in terms of movement, secretion, enzyme production, gene expression, etc.) as a result of an extracellular stimulus. [GOC:go_curators]
XLOC_063811	GO:0009991	response to extracellular stimulus	Any process that results in a change in state or activity of a cell or an organism (in terms of movement, secretion, enzyme production, gene expression, etc.) as a result of an extracellular stimulus. [GOC:go_curators]
XLOC_066861	GO:0009991	response to extracellular stimulus	Any process that results in a change in state or activity of a cell or an organism (in terms of movement, secretion, enzyme production, gene expression, etc.) as a result of an extracellular stimulus. [GOC:go_curators]
XLOC_073642	GO:0009991	response to extracellular stimulus	Any process that results in a change in state or activity of a cell or an organism (in terms of movement, secretion, enzyme production, gene expression, etc.) as a result of an extracellular stimulus. [GOC:go_curators]
XLOC_005426	GO:0080027	response to herbivore	Any process that results in a change in state or activity of a cell or an organism (in terms of movement, secretion, enzyme production, gene expression, etc.) as a result of a stimulus from a herbivore. [PMID:18987211]

XLOC_021897	GO:0080027	response to herbivore	Any process that results in a change in state or activity of a cell or an organism (in terms of movement, secretion, enzyme production, gene expression, etc.) as a result of a stimulus from a herbivore. [PMID:18987211]
XLOC_030038	GO:0080027	response to herbivore	Any process that results in a change in state or activity of a cell or an organism (in terms of movement, secretion, enzyme production, gene expression, etc.) as a result of a stimulus from a herbivore. [PMID:18987211]
XLOC_030886	GO:0080027	response to herbivore	Any process that results in a change in state or activity of a cell or an organism (in terms of movement, secretion, enzyme production, gene expression, etc.) as a result of a stimulus from a herbivore. [PMID:18987211]
XLOC_035144	GO:0080027	response to herbivore	Any process that results in a change in state or activity of a cell or an organism (in terms of movement, secretion, enzyme production, gene expression, etc.) as a result of a stimulus from a herbivore. [PMID:18987211]
XLOC_054355	GO:0080027	response to herbivore	Any process that results in a change in state or activity of a cell or an organism (in terms of movement, secretion, enzyme production, gene expression, etc.) as a result of a stimulus from a herbivore. [PMID:18987211]
XLOC_071313	GO:0080027	response to herbivore	Any process that results in a change in state or activity of a cell or an organism (in terms of movement, secretion, enzyme production, gene expression, etc.) as a result of a stimulus from a herbivore. [PMID:18987211]
XLOC_076779	GO:0080027	response to herbivore	Any process that results in a change in state or activity of a cell or an organism (in terms of movement, secretion, enzyme production, gene expression, etc.) as a result of a stimulus from a herbivore. [PMID:18987211]

XLOC_046758	GO:0002238	response to molecule of fungal origin	Any process that results in a change in state or activity of an organism (in terms of movement, secretion, enzyme production, gene expression, etc.) as a result of a stimulus by molecules of fungal origin such as chito-octamer oligosaccharide. [GOC:rl, GOC:sm]
XLOC_000267	GO:0000304	response to singlet oxygen	Any process that results in a change in state or activity of a cell or an organism (in terms of movement, secretion, enzyme production, gene expression, etc.) as a result of a singlet oxygen stimulus. Singlet oxygen is a dioxygen (O2) molecule in which two 2p electrons have similar spin. Singlet oxygen is more highly reactive than the form in which these electrons are of opposite spin, and it is produced in mutant chloroplasts lacking carotenoids and by leukocytes during metabolic burst. [GOC:krc, ISBN:0124325653, ISBN:0198506732]
XLOC_020442	GO:0000304	response to singlet oxygen	Any process that results in a change in state or activity of a cell or an organism (in terms of movement, secretion, enzyme production, gene expression, etc.) as a result of a singlet oxygen stimulus. Singlet oxygen is a dioxygen (O2) molecule in which two 2p electrons have similar spin. Singlet oxygen is more highly reactive than the form in which these electrons are of opposite spin, and it is produced in mutant chloroplasts lacking carotenoids and by leukocytes during metabolic burst. [GOC:krc, ISBN:0124325653, ISBN:0198506732]
XLOC_029375	GO:0000304	response to singlet oxygen	Any process that results in a change in state or activity of a cell or an organism (in terms of movement, secretion, enzyme production, gene expression, etc.) as a result of a singlet oxygen stimulus. Singlet oxygen is a dioxygen (O2) molecule in which two 2p electrons have similar spin. Singlet oxygen is more highly reactive than the form in which these electrons are of opposite spin, and it is produced in mutant chloroplasts lacking carotenoids and by leukocytes during metabolic burst. [GOC:krc, ISBN:0124325653, ISBN:0198506732]
XLOC_040000	GO:0001104	RNA polymerase II transcription cofactor activity	Interacting selectively and non-covalently with an RNA polymerase II (RNAP II) regulatory transcription factor and also with the RNAP II basal transcription machinery in order to modulate transcription. Cofactors generally do not bind DNA, but rather mediate protein-protein interactions between regulatory transcription factors and the basal RNAP II transcription machinery. [GOC:txnOH, PMID:10213677, PMID:16858867]
XLOC_070320	GO:0007264	small GTPase mediated signal transduction	Any series of molecular signals in which a small monomeric GTPase relays one or more of the signals. [GOC:mah]

XLOC_017361	GO:0010686	tetracyclic triterpenoid biosynthetic process	The chemical reactions and pathways resulting in the formation of tetracyclic triterpenoid compounds, terpenoids with six isoprene units and 4 carbon rings. [GOC:tair_curators]
XLOC_066757	GO:0017053	transcriptional repressor complex	A protein complex that possesses activity that prevents or downregulates transcription. [GOC:mah]
XLOC_075298	GO:0017053	transcriptional repressor complex	A protein complex that possesses activity that prevents or downregulates transcription. [GOC:mah]
XLOC_071610	GO:0003743	translation initiation factor activity	Functions in the initiation of ribosome-mediated translation of mRNA into a polypeptide. [ISBN:0198506732]
XLOC_070774	GO:0016429	tRNA (adenine-N1-)-methyltransferase activity	Catalysis of the reaction: S-adenosyl-L-methionine + tRNA = S-adenosyl-L-homocysteine + tRNA containing N1-methyladenine. [EC:2.1.1.36]
XLOC_000893			
XLOC_001238			
XLOC_001261			
XLOC_001450			
XLOC_001802			
XLOC_001896			
XLOC_002279			
XLOC_003301			
XLOC_003451			
XLOC_003792			
XLOC_004205			
XLOC_004444			
XLOC_004483			
XLOC_005044			

XLOC_005463
XLOC_006237
XLOC_007198
XLOC_008468
XLOC_008566
XLOC_008615
XLOC_008704
XLOC_008713
XLOC_008789
XLOC_009039
XLOC_009047
XLOC_009088
XLOC_009148
XLOC_010148
XLOC_010395
XLOC_010416
XLOC_011301
XLOC_011401
XLOC_011622
XLOC_011826
XLOC_012328
XLOC_012386
XLOC_012400
XLOC_013292
XLOC_013339
XLOC_013623
XLOC_013639
XLOC_013729
XLOC_013791
XLOC_014094
XLOC_014714
XLOC_015085
XLOC_016876
XLOC_017223
XLOC_017269
XLOC_018460
XLOC_018583
XLOC_018898
XLOC_019220
XLOC_019505
XLOC_019526
XLOC_020078
XLOC_020410
XLOC_020422

XLOC_020463
XLOC_020496
XLOC_020664
XLOC_021305
XLOC_021396
XLOC_021429
XLOC_021765
XLOC_022187
XLOC_022534
XLOC_024108
XLOC_024285
XLOC_024420
XLOC_024660
XLOC_024801
XLOC_024826
XLOC_024907
XLOC_025259
XLOC_025278
XLOC_025312
XLOC_025332
XLOC_025494
XLOC_025888
XLOC_026094
XLOC_026544
XLOC_026666
XLOC_026837
XLOC_026922
XLOC_027306
XLOC_027625
XLOC_028119
XLOC_028458
XLOC_028545
XLOC_029130
XLOC_029279
XLOC_029615
XLOC_029617
XLOC_029908
XLOC_030250
XLOC_030896
XLOC_031038
XLOC_031403
XLOC_031801
XLOC_032308
XLOC_032361

XLOC_032435
XLOC_032477
XLOC_032515
XLOC_032545
XLOC_032606
XLOC_032643
XLOC_032723
XLOC_032754
XLOC_033219
XLOC_033738
XLOC_033910
XLOC_035080
XLOC_035101
XLOC_035694
XLOC_035796
XLOC_036036
XLOC_037325
XLOC_037367
XLOC_038442
XLOC_040357
XLOC_040852
XLOC_040963
XLOC_041040
XLOC_041870
XLOC_042208
XLOC_042270
XLOC_042275
XLOC_042449
XLOC_042875
XLOC_043378
XLOC_043389
XLOC_043457
XLOC_043766
XLOC_043932
XLOC_043958
XLOC_045508
XLOC_047329
XLOC_047598
XLOC_047674
XLOC_047716
XLOC_047988
XLOC_051775
XLOC_053440
XLOC_054029

XLOC_054412
XLOC_054985
XLOC_056147
XLOC_056178
XLOC_056246
XLOC_056656
XLOC_056891
XLOC_057170
XLOC_057343
XLOC_058210
XLOC_059063
XLOC_059107
XLOC_059426
XLOC_060158
XLOC_060189
XLOC_060203
XLOC_060262
XLOC_060380
XLOC_060395
XLOC_060547
XLOC_060947
XLOC_061152
XLOC_061882
XLOC_062698
XLOC_064584
XLOC_064693
XLOC_064844
XLOC_064848
XLOC_066010
XLOC_066095
XLOC_066372
XLOC_066647
XLOC_066826
XLOC_066977
XLOC_067119
XLOC_067189
XLOC_067414
XLOC_067752
XLOC_068683
XLOC_070203
XLOC_070794
XLOC_071071
XLOC_071318
XLOC_071397

XLOC_071807
XLOC_072175
XLOC_072401
XLOC_072428
XLOC_072536
XLOC_072578
XLOC_072903
XLOC_073594
XLOC_073610
XLOC_074256
XLOC_074411
XLOC_075932
XLOC_075970
XLOC_076299
XLOC_076344
XLOC_076691
XLOC_076780
XLOC_076951
XLOC_076952
XLOC_077106
XLOC_077108
XLOC_077146
